# Insights on the SARS-CoV-2 genome variability: the lesson learned in Brazil and its impacts on the future of pandemics

**DOI:** 10.1099/mgen.0.000656

**Published:** 2021-11-03

**Authors:** Victória Riquena Grosche, Igor Andrade Santos, Giulia Magalhães Ferreira, João Victor Rodrigues Dutra, Larissa Catharina Costa, Nilson Nicolau-Junior, Artur Trancoso Lopo Queiroz, Diego Pandeló José, Ana Carolina Gomes Jardim

**Affiliations:** ^1^​ São Paulo State University, São José do Rio Preto, São Paulo, Brazil; ^2^​ Federal University of Uberlândia, Uberlândia, Minas Gerais, Brazil; ^3^​ Federal University of Triângulo Mineiro, Campus Universitário Iturama, Iturama, Minas Gerais, Brazil; ^4^​ Center of Data and Knowledge Integration for Health (CIDACS), Gonçalo Moniz Institute, Oswaldo Cruz Foundation, Salvador, Bahia, Brazil

**Keywords:** COVID-19, epidemiology, genomic sequence analysis, SARS-CoV-2, UTR

## Abstract

Since the beginning of the SARS-CoV-2 spread in Brazil, few studies have been published analysing the variability of viral genome. Herein, we described the dynamic of SARS-CoV-2 strains circulating in Brazil from May to September 2020, to better understand viral changes that may affect the ongoing pandemic. Our data demonstrate that some of the mutations identified are currently observed in variants of interest and variants of concern, and emphasize the importance of studying previous periods in order to comprehend the emergence of new variants. From 720 SARS-CoV-2 genome sequences, we found few sites under positive selection pressure, such as the D614G (98.5 %) in the spike, that has replaced the old variant; the V1167F in the spike (41 %), identified in the P.2 variant that emerged from Brazil during the period of analysis; and I292T (39 %) in the N protein. There were a few alterations in the UTRs, which was expected, however, our data suggest that the emergence of new variants was not influenced by mutations in UTR regions, since it maintained its conformational structure in most analysed sequences. In phylogenetic analysis, the spread of SARS-CoV-2 from the large urban centres to the countryside during these months could be explained by the flexibilization of social isolation measures and also could be associated with possible new waves of infection. These results allow a better understanding of SARS-CoV-2 strains that have circulated in Brazil, and thus, with relevant infomation, provide the potential viral changes that may have affected and/or contributed to the current and future scenario of the COVID-19 pandemic.

## Data Summary

All the Brazilian SARS-CoV-2 genome sequences were selected in the Global Initiative on Sharing All Influenza Data (GISAID) database. All the supporting data have been provided through supplementary data files.

**Impact Statement**

The severe acute respiratory syndrome coronavirus 2 (SARS-CoV-2) emerged in late 2019 and spread across the world. Brazil is an important centre of the COVID-19 pandemic, with significant infection and death rates. However, a limited number of works reported viral strains, genomic signatures or mutations in the circulating virus in the country, mainly reporting and characterizing variants that emerged. Herein we analysed the SARS-CoV-2 genome strains circulating in Brazil from May to September 2020 using 720 sequences deposited in the GISAID database to better understand the variability in the viral genome during this period and its impacts on the current and future scenario of the pandemic in Brazil. Our data demonstrated that some of the mutations identified here are present in variants of interest and variants of concern (VOCs), and emphasize the importance of studying previous periods in order to comprehend the emergence of new variants. The most frequent substitutions identified have been described globally, such as the G614-carrying virus and V1176F, enhancing viral transmissibility and infectivity. There were a few alterations in the UTRs, which was expected, however, our data suggest that the emergence of new variants were not influenced by mutations in UTR regions, since it maintained its conformational structure in most analysed sequences. In phylogenetic analysis, the spread of SARS-CoV-2 from the large urban centres to the countryside during these months could be explained by the flexibilization of social isolation measures and also be associated with possible new waves of infection. The results described here demonstrate possible genomic signatures, suggest a geography transmission chain, viral adaptability, and emphasize the importance of active viral genomic surveillance, since important mutations in VOCs were found in periods before its appearance.

## Introduction

The severe acute respiratory syndrome coronavirus 2 (SARS-CoV-2) is classified as a β-coronavirus. The virus particle comprises a single positive-stranded RNA genome of approximately 30 kb associated with a nucleocapsid, surrounded by a lipid envelope inserted of the spike glycoprotein [1]. The SARS-CoV-2 viral genome is translated to produce nonstructural proteins (nsps) from two ORFs, ORF1a and ORF1b. The ORF1a encodes the polyprotein pp1a that is cleaved in 11 nsps, while the ORF1b encodes the polyprotein pp1ab, which is cleaved into 15 nsps. The nsps assemble to form a replicase-transcriptase complex (RTC) responsible for RNA synthesis, replication, and transcription of nine subgenomic RNAs (sgRNAs) [2–4], that act as mRNAs for translation of structural (S, E, and M) and accessory (3 a, 6, 7 a, 7b, 8, and 10) proteins [3].

The SARS-CoV-2 shares a high nucleotide sequence homology with the SARS-CoV, the SARS-like bat coronaviruses bat-SL-CoVZC45, and the Middle East respiratory syndrome coronavirus (MERS-CoV) [5]. When compared to other CoVs, SARS-CoV-2 presents higher transmissibility, which allowed the rapid and efficient spread of the virus [6]. Consequently, mutations may raise and contribute to the appearance of variants of interest (VOI) or variants of concern (VOC), associated with reduced antibody neutralization, increased transmissibility and/or disease severity [7]. As the outcome, higher mortality rates or new outbreaks might be expected [8, 9]. Therefore, tracking the SARS-CoV-2 genome variability is essential to strategically combat COVID-19. Currently, there are several nomenclature suggestions to classify SARS-CoV-2 lineages, however, the classification used by the Centers for Disease Control and Prevention (CDC) and World Health Organization (WHO) is based on Rambaut and coworkers [10]. The proposal is labelled considering the major lineages (A and B), and the descendant lineages assigned by numerical values (B.1, B.1.1, B.1.1.28, among others) [10]. There are six lineages derived from lineage A (denoted A.1–A.6), two sublineages of A.1 (A.1.1 and A.3), and 16 lineages derived from lineage B. Furthermore, lineage B.1 is the predominant global lineage and has been subdivided into more than 70 sublineages [10].

The *Nodovirales* order to which the *Coronaviruses* genus belongs is characterized by large RNA genome, and even though the RNA-dependent RNA polymerase (RdRP – nsp12) is formed by structure complex of RdRp/nsp7/nsp8 to ensure fidelity in transcription, a high mutation rate has been observed [11, 12]. This effect might be the outcome of ineffective control measures, low vaccination rates, globalization that applies a powerful selective pressure, favouring the appearance of SARS-CoV-2 variants, as has recently been seen [13, 14]. Together, these factors favour genome variations during the SARS-CoV-2 replication cycle, and since it is a positive single-strand RNA viral genome the mutation is expected to be dramatically high, mainly in key replication and structural proteins, such as the spike (S) and the nucleocapsid (N) [15]. These regions of the SARS-CoV-2 genome are constantly under selective pressure resulting in selective mutations to guarantee viral adaptation, replication, and virulence modulation [16, 17]. The mutation process is continuously affected by physical and chemical interferences as well as recombination events, which leads to single nucleotide variants, deletions, and insertions, varying amino acid sequence and protein structure [16].

In this context, the WHO in collaboration with partners, have been monitoring and assessing the evolution of SARS-CoV-2, and defined the emergence of variants that posed an increased risk to global public health prompted the characterization of specific VOIs and VOCs [18]. For the assignment of these variants, it is mainly reported substitutions in the spike protein residues 319–541 (receptor-binding domain – RBD) and 613–705 (the S1 part of the S1/S2 junction and a small stretch on the S2 side) [18, 19]. The S1 subunit comprises an N-terminal domain and the RBD; and the S2 subunit includes the fusion peptide (FP), heptapeptide repeat sequence 1 (HR1), HR2, TM domain, and cytoplasm domain [20, 21]. These regions are responsible for receptor binding and membrane fusion, respectively [21]. To date, the WHO recognizes four VOCs and seven VOIs. According to the new labels, VOCs Alpha, Beta, Gamma, and Delta correspond to, respectively, lineages B.1.1.7 (and B.1.1.7+E484K), B.1.351, P.1, and B.1.617.2 [18]. VOIs Epsilon, Zeta, Eta, Theta, Iota, Kappa, and Lambda correspond to, respectively, lineages B.1.427/B.1.429, P.2, B.1.525, P.3, B.1.526, B.1.617.1, and C.37 [18]. Among them, the variants P.1 and P.2, first isolated in Brazil possess substitutions of interest in the S protein, including K417T, E484K, N501Y, D614G, and H655Y [19]. Although the mutation V1176F it is not described by the WHO as a substitution of interest, its presence in the P.2 variant, and possibly in other variants under monitoring, has been drawing attention, since it interferes in the HR2 region of the spike, and may favour entry of the virus into the cell [22].

Brazil is an important centre of the COVID-19 pandemic, with significant infection and death rates [23]. To date, the available works reporting viral strains, genomic signatures or mutations in the circulating virus in Brazil in correlation with epidemiological data are limited [24, 25]. Although De Souza and collaborators contextualized epidemiological, demographic, and clinical findings for COVID-19 cases during the very beginning of the pandemics in Brazil [26], there is a lack of genomic analysis in the following months. Other studies provided information about genomics and epidemiology focusing on specific Brazilian regions, as in Manaus, where the P.1 variant has emerged, or in the South of the country [27–29].

Mutations in amino acids can result in more virulent or infectious viral strains and can affect the ongoing pandemic. This effect was seen recently in the emergence of VOIs and VOCs [30]. Herein, we analysed the SARS-CoV-2 genome strains circulating in Brazil from May to September 2020 using 720 sequences deposited in the Global Initiative on Sharing All Influenza Data (GISAID) database up to 15 March 2021, to better understand the variability in the viral genome. Our data demonstrate that some of the mutations identified here are present in currently circulating VOIs and VOCs, and emphasize the importance of studying previous periods in order to better understand the emergence of new variants. The most frequent substitutions identified have been described globally, such as the G614-carrying virus and V1176F, enhancing viral transmissibility and infectivity. Data generated herein suggest potential genomic signatures and demonstrated that few SARS-CoV-2 genomic sites are under positive selection pressure. Therefore, we discussed the impact of substitutions in SARS-CoV-2 genome and how it can impact the emergence of new variants.

## Methods

### Collation and alignment of Brazilian SARS-CoV-2 genome sequences

The SARS-CoV-2 genome sequences were selected in the GISAID database [31] employing the filters for the location ‘Brazil’ and date of collection of the swab samples from 1 May to 30 September 2020. All available sequences were selected on 15 March 2021, with a higher coverage (>75%). Additional information about the samples was analysed including age, gender, patient status, and genome lineage, using GraphPad Prism v8.0.1 (www.graphpad.com), employing a confidence interval of 95%. A dataset with 720 sequences was generated and submitted to multiple sequence alignment employing the mafft [32]. The sequences available in the GISAID database on 15 March 2021, with the swab sample collection up to 30 April, were downloaded and submitted to multiple sequence alignment as cited above for comparison with the period from May to September. All sequences of the dataset were also submitted into Pangolin COVID-19 Lineage Assigner (https://pangolin.cog-uk.io/; version V2.3.8) to confirm the lineages of each sequence.

### Amino acid substitutions and selection pressure analysis

The aligned dataset was translated into amino acids using BioEdit software [33]. All variations in amino acid were catalogued into Table S1, available in the online version of this article, and the frequency of variation was calculated for each amino acid change using the equation ( N÷T×100 ), in which *N* means the number of sequences with that variation and *T* the total of sequences in the dataset. The selective pressure analysis was performed on the above reported SARS-CoV-2 protein-coding sequence sub-sets through the Datamonkey Adaptive Evolution Server [34–37]. The aim was to characterize the SARS-CoV-2 variations in Brazil, from May to September 2020, its evolutionary dynamics and to identify and localize statistically supported positive and negative selective pressure sites. In order to predict the impact of each substitution in protein selection, we employed the model Fast Unconstrained Bayesian AppRoximation (FUBAR) [38]. The method selected infers the nonsynonymous (d*N*) and synonymous (d*S*) substitution rates per-site basis in large datasets, based on the assumption that a pervasive selection pressure is constant in the entire phylogeny. The results are demonstrated in Table S2 and only the statistically supported selective pressure sites considering Bayes factor (BF) >30 were reported [39].

### Analysis of secondary structure of 5′ and 3′ UTR

Dataset with complete genomic sequences was edited for analysis of the 5′ untranslated regions (UTR), which included the 12 initials codons of NSP1 [40], and 3′ UTR, which included the ORF10 located upstream of the 3′ UTR. We used the genomic coordinates of the reference sequence (NC_045512.2). Then, 5′ and 3′ datasets were submitted to the RNA-fold platform [41] to analyse predictions of secondary structures.

### Phylogenetic analyses of SARS-CoV-2 genome sequences

Sequences used for analysis had the genome coverage >75%. Sequence alignment was performed using mafft [32, 42] and visually inspected in Aliview [43]. A phylogenetic tree was reconstructed using maximum-likelihood analysis (ML), GTR+F+R2 substitution model in IQ-Tree [44]. The best fitted substitution model was tested with ModelFinder [45] as implemented in IQ-Tree. Trees were viewed in FigTree and the temporal signal of this phylogeny was assessed in Tempest [46]. The CIPRES Science gateway platform [47] was used to run mafft and IQ-tree.

## Results

### Epidemiological features of COVID-19 in Brazil

**Fig. 1.**
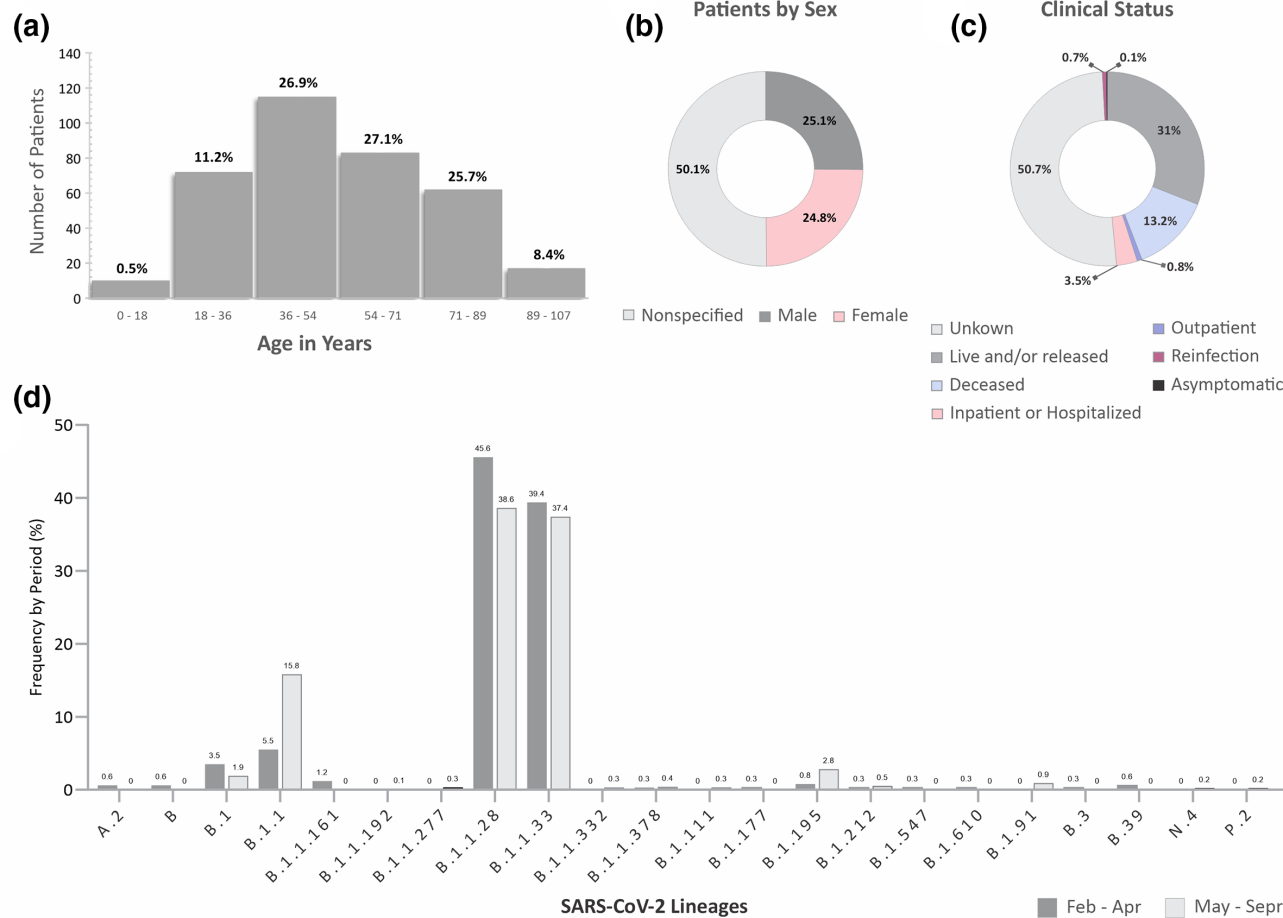
Epidemiological data of SARS-CoV-2 sequences of samples from infected patients in Brazil between May and September 2020, available on the GISAID database on 15 March 2021. (a) Frequency of distribution of individuals with SARS-CoV-2 infection according to age. (b) Gender information of individuals with SARS-CoV-2 in Brazil. (c) Clinical status of patients on the sample collection date. (d) Prevalence of lineages and sublineages among Brazilian available sequences in the periods from February to April or from May to September 2020.

Currently, there is limited information related to the genome sequence variability and epidemiology of COVID-19 in Brazil. Candido and coworkers described characteristics of the SARS-CoV-2 genome and its spread in Brazil [24] from February to April of 2020. Therefore, here we analysed the available data in the GISAID database, focusing on the sequences and information of samples from Brazilian patients collected between May and September 2020, available on 15 March 2021. From the information provided of the 356 sequences with available clinical status, almost 88.2 % of the individuals were over 36 years old (IC95 % 113.1–68.8 %), the ones between 0–18 years comprehended 0.55 % (IC95 % 096–0.32 %), and 11.2 % of them were between 20–39 years (IC95 % 16.0–7.8 %) (Fig. 1a). It was observed that 25.1% of individuals were male (IC95 % 28.4–22.1%), 24.8 % were female (IC95 % 28.0–21.7  %), and 50.1% were not specified or unknown (IC95 % 53.8–46.5 %) (Fig. 1b). From the available data, 0.1% of individuals were classified as asymptomatic (IC95 % 0.8–0.007 %), 31% as alive and/or released (IC95 % 34.4–27.7 %), 3.5% as hospitalized (IC95 % 5.1–2.4 %), 13.2% as deceased (IC95 % 15.9–10.9%), 0.8 % as outpatients (IC95 % 1.8–0.4 %), 0.7 % as reinfected (IC95 % 1.6–0.3%), and 50.7 % as unknown (IC95 % 54.3–47.0%) (Fig. 1c).

Most of the 720 sequences studied were included in lineage B, as stated by Pangolin analysis, according to the recently proposed SARS-CoV-2 lineage nomenclature [10]. The most common sublineage was B.1.1.28 with 38.6% of prevalence (IC 95 % 42.2–35.1 %), followed by sublineage B.1.1.33, with 37.4 % (IC 95 % 41–33.9 %), and 15.8 % of B.1.1 (IC 95 % 15.8–13.3 %). We also identified sequences from lineages P.2 and N.4 (Fig. 1d). In the previous period (February to April 2020), sequences from lineages A and B were identified; the most prevalent sublineages were also B.1.1.28, and B.1.1.33, with a frequency of 45.6 % and 39.4%, respectively (Fig. 1d).

### Follow-up of the amino acid substitutions and selection pressure analysis in the sequences over time

**Fig. 2.**
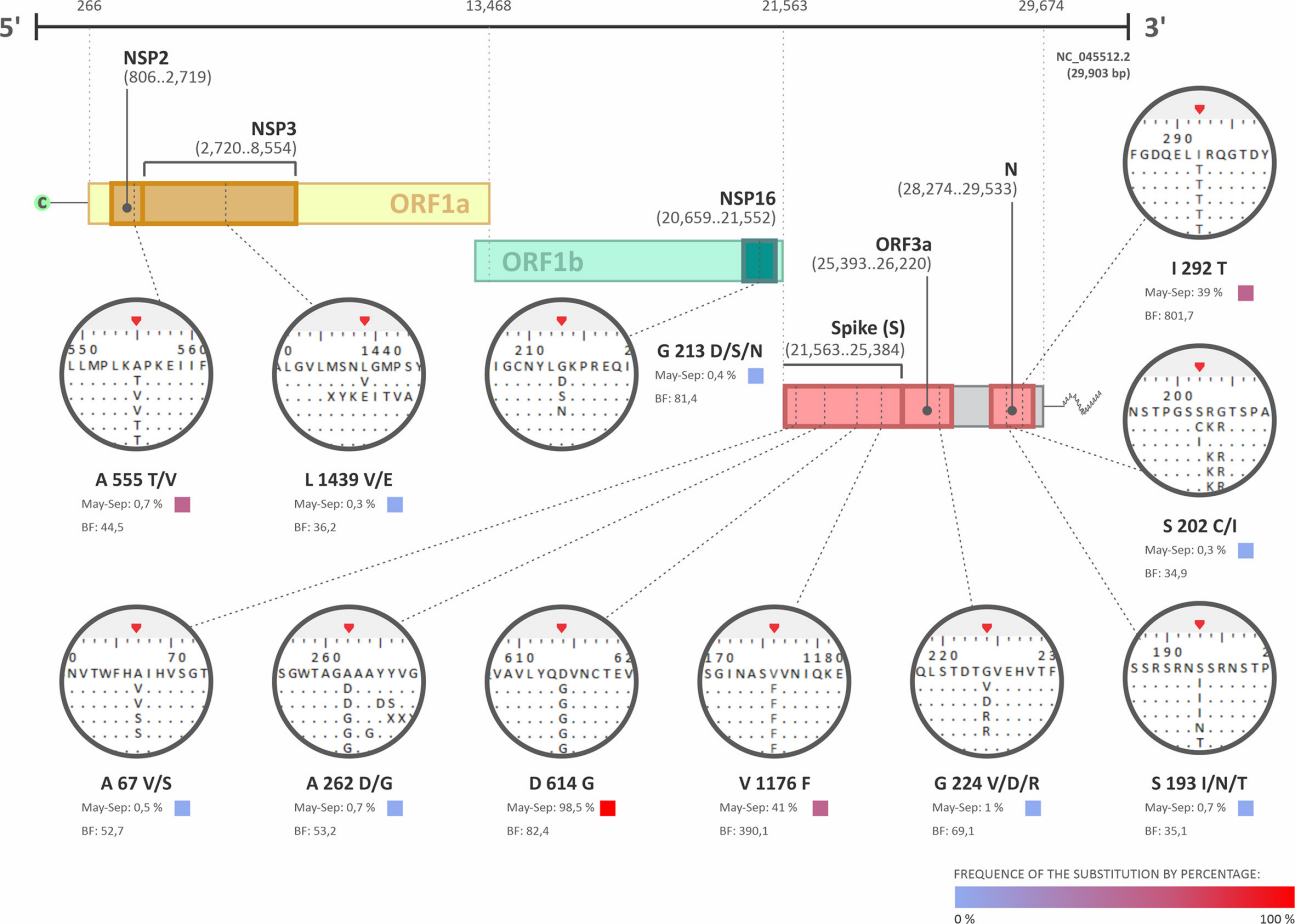
The most relevant amino acid substitutions in Brazilian SARS-CoV-2 sequences that presented positive selection pressure with a Bayes factor (BF) >30 (BF of 30–100: very strong evidence for positive selection pressure; BF >100: decisive evidence for positive selection pressure). The sequences analysed were related to samples collected between May and September 2020, and were available on the GISAID database on 15 March 2021. NC_045512.2, the first sequence isolated from Wuhan, PR China, was used as the reference sequence. The frequency of the substitution is presented as percentage, where 0 % is represented in blue, and 100 % in red. The red triangles in the expanded image of the dataset indicated the positively selected site.

Amino acid substitutions were investigated by comparing the datasets of available sequences on 15 March 2021, related to samples collected from May to September or from February to April 2020, with the genome reference NC_045512.2, the first isolated in Wuhan, PR China. The results of analysis of the period from May to September 2020 demonstrated that amino acid substitutions were randomly observed in most of the viral proteins (Table S1). The most frequent substitutions among sequences were L71F (22.6 %) in the NSP7; P303L (99 %) in the RNA-dependent RNA polymerase (RdRp); D614G (98.5 %) and V1176F (41%) in the spike; I33T (37.4 %) in the ORF6; R203K (93.5 %), G204R (93.8 %), and I292T (39 %) in the N protein (Table S1). From those, only the substitutions D614G and V1176F in the spike, and I292T in the N protein, presented positive selection pressure, according to the FUBAR analysis, with a Bayes factor (BF) of 82.4, 390.1, and 801.7, respectively (Fig. 2 and Table S2).

Overall, the selective pressure analysis varied considerably according to the genes. The analysis conducted on NSP1, NSP8, NSP9, NSP10, ORF7a, and ORF8 sub-sets indicated only negatively selected sites (Table S2). However, NSP5 presented positively selected sites. Additionally, NSP7, NSP11, E, ORF7b, and ORF10 showed neither positive nor negative sites (Table S2).

Selective pressure analysis conducted on NSP2 demonstrated one positively selected site, 555 (A; T/V), with a BF of 44.5 (BF of 30–100: very strong evidence for positive selection pressure; BF >100: decisive evidence for positive selection pressure) and three negatives. NSP3 revelled four positively selected sites, one of them, 1439 (L; V/E), with a BF of 36.2, and eight negatives. NSP16 showed one positively selected site, 213 (G; D/S/N), with a BF of 81.4 and six negatives. As expected, the spike protein revelled four positively selected sites at positions 67 (A; V/S), with a BF of 52.7, 262 (A; D/G), with a BF of 53.2, 614 (D; G), with a BF of 82.4, and at 1176 (V; F), with a BF of 390.1, and two other positively selected sites with a lower BF. Spike also presented 13 negative sites. Selective pressure analysis conducted on ORF3a found two positively selected sites, one of them at position 224 (G; V/D/R), with a BF of 69.1, and three negatives. Finally, the N protein showed three relative positively selected sites, at positions 193 (S; I/T/N), with a BF of 35.1, 202 (S; C/I), with a BF of 34.9, and at 293 (I; T), with the highest BF of 801.7, and the other three with a lower BF. The N protein also presented five negative selective pressure sites (Fig. 2 and Table S2). Further information on proteins with positive and negative selective pressures, however with a BF <10 in positive sites, are described in Table S2.

**Fig. 3.**
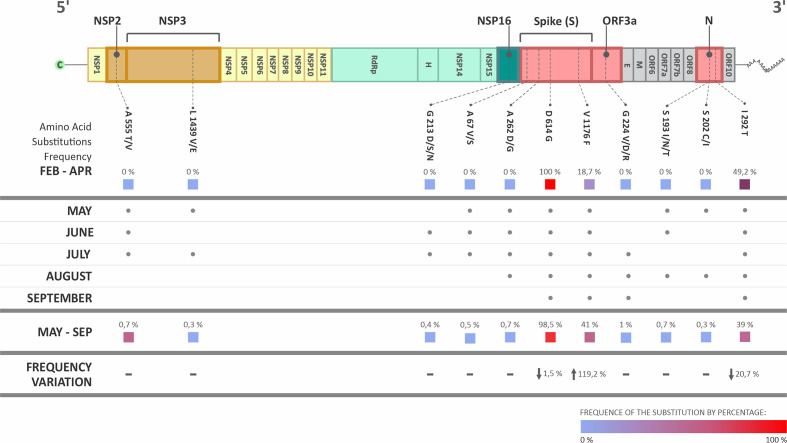
Variation of the frequency of substitutions at positively selected sites detected in Brazilian SARS-Cov-2 sequences deposited on the GISAID database, collected between May and September, 2020, available up to 15 March 2021. Data of the same sites and amino acid substitution identified in samples of the previous period, from February to April 2020 (available on the same date) is also shown.

Mutations in S and N proteins are constantly under selective pressure and characterize most of the VOCs and VOIs described to date, since they induce higher transmissibility and replication gain, resulting in adaptative fitness [16, 48]. Interestingly, only three of the amino acid substitutions with positive selective pressure identified here were present at the previous period, from February to April 2020: D614G (100 % of frequency) and V1176F (18.7 %) in the spike; and I292T (49.2 %) in the N (Fig. 3). In comparison with the sequences of the period from May to September 2020, D614G had a decrease of 1.5 % in the frequency, V1176F increased 119.2 % and I292T, although its high BF, decreased 20.7 % (Fig. 3). We were able to find these three substitutions in every month, from May to September, as shown in Fig. 3.

### Conservation of 3′ and 5′ UTR secondary structures of SARS-CoV-2 over time

**Fig. 4.**
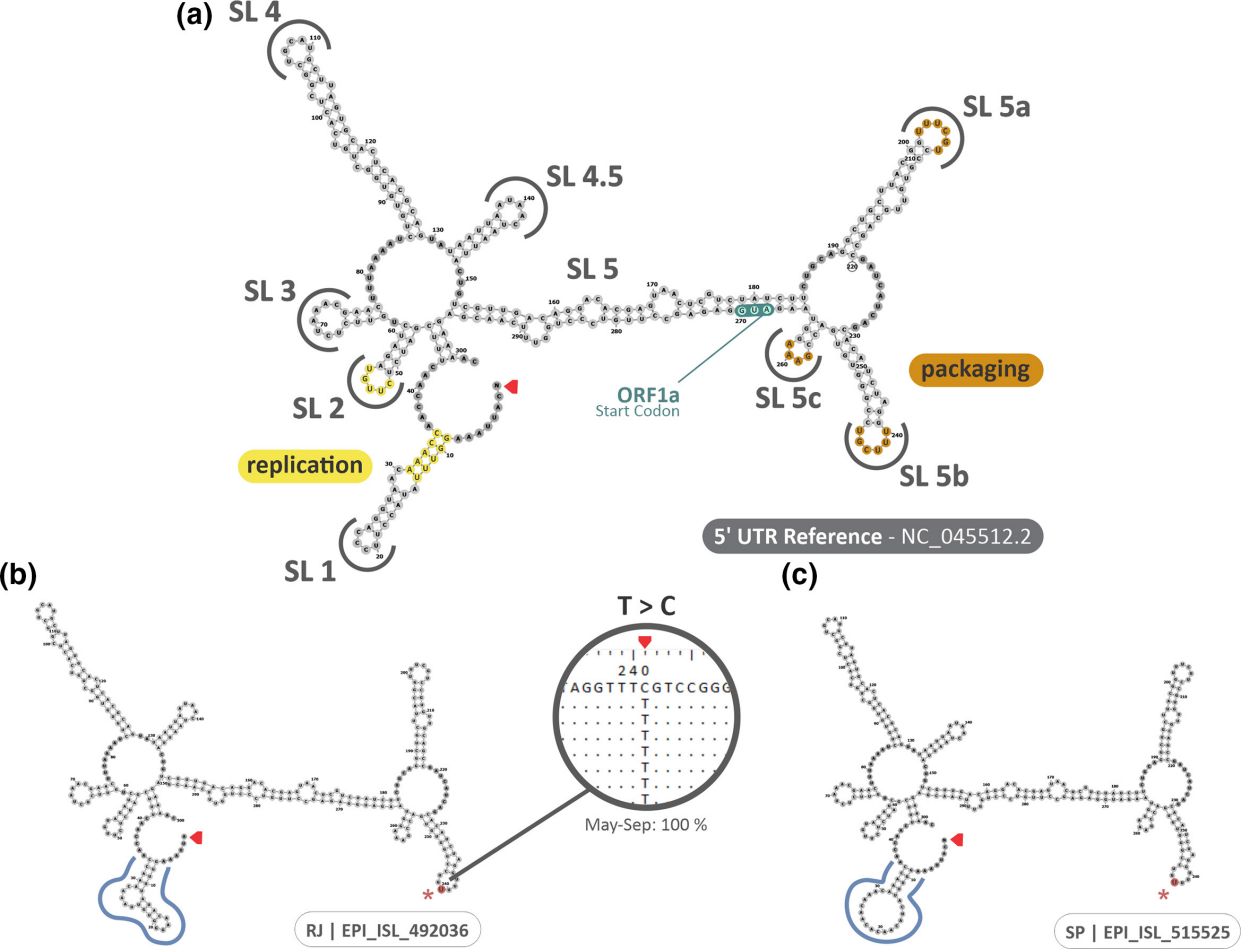
Comparison of the secondary structure of the 5′ UTR of SARS-CoV-2. (a) The reference sequence. (b, c) The two isolated sequences with substantial changes outlined in blue. The red arrows indicate the beginning of the 5′ UTR region. The nucleotides highlighted in yellow indicate regions possibly related to viral replication, and the ones in orange indicate the possible structures involved in the packaging of the virus. The ORF1a start codon is highlighted in green.

The UTR structures' influence in viral replication efficacy, and its secondary structure are essential to stabilize and guarantee the host ribosome activity in translating viral genome [49]. Nucleotide variations in these regions can interfere and/or impair viral replication and genome translation [49]. Analysis of the sequences using the RNAfold platform [41] showed a high conservation in both 3′ UTR and 5′ UTR structures, in relation to the reference sequence (NC_045512.2). In case of incomplete sequences, a notation was made to complete them using the NC_045512.2 sequence as the reference. The nucleotide substitution C>T at the position 241 of the 5′ UTR region was identified in 100 % of the sequences related to the period from May to September (Fig. 4). Between February and April, we observed the substitution C>T 241 in 73.6 % of the sequences, an increase of 35.9 % compared to the previous period. Despite the high prevalence, these specific variations did not result in any change of conformation of the secondary structures (Fig. 4).

**Fig. 5.**
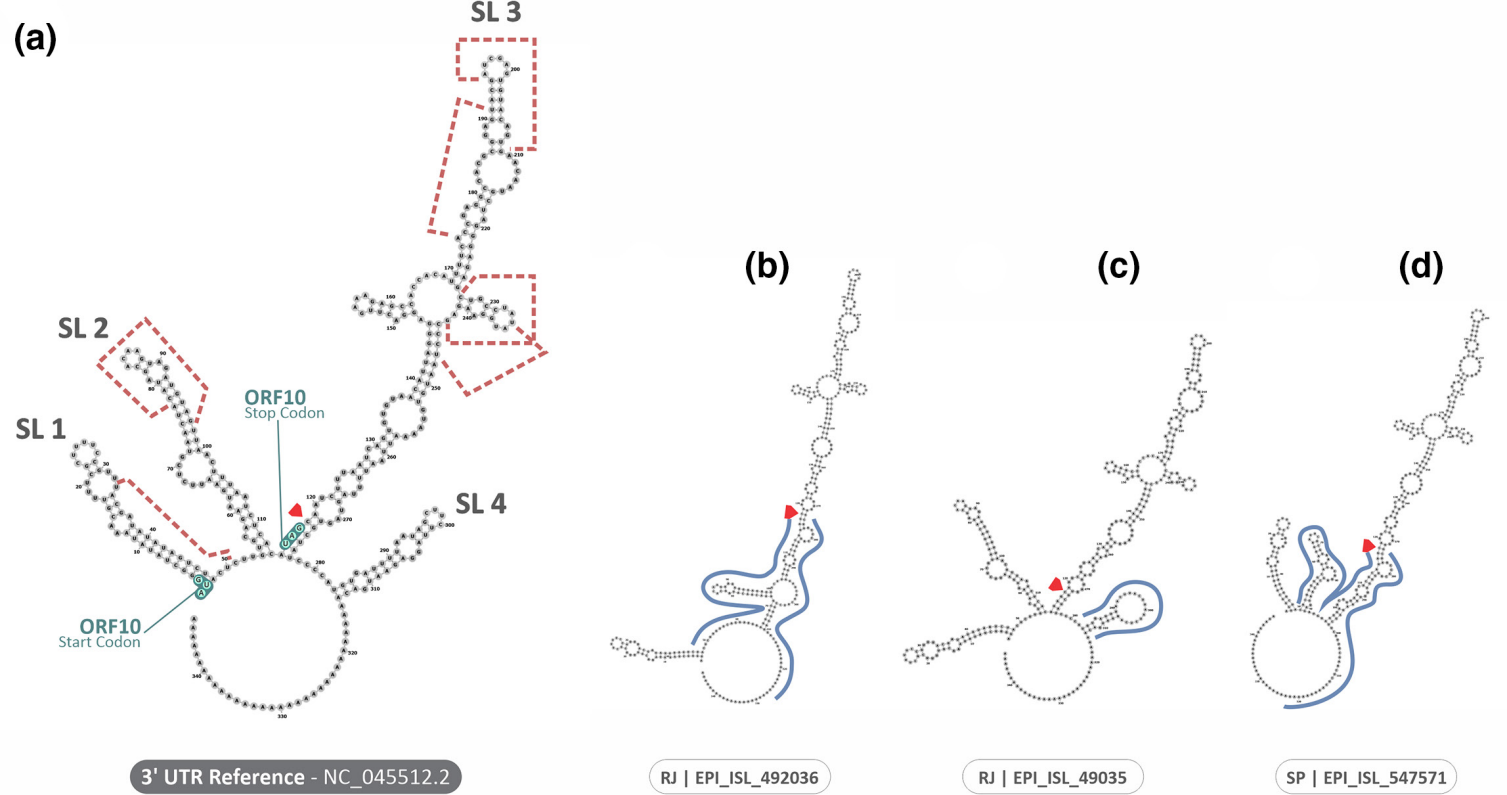
Comparison of the secondary structure of the 3′ UTR of SARS-CoV-2. (a) The reference sequence. (b–d) The three isolated sequences with substantial changes outlined in blue. The red arrows indicate the beginning of the 3′ UTR region. The ORF10 start and stop codons are highlighted in green. Red dashed lines indicate structures that possibly interact with host miRNAs.

Two sequences (RJ-EPI_ISL_492036|2020-06-01 and SP-EPI_ISL_515525|2020-06-30) presented substantial variations in the nucleotide sequence of 5′ UTR, resulting in small changes in the stem-loop 1 (SL1) structure (Fig. 4). The 3′ UTR region presented more variations, which were observed in the structures SL2, SL3, and SL4 of the sequences SP-EPI_ISL_547571|2020-06-01 and RJ-EPI_ISL_492036|2020-06-01 (Fig. 5). Furthermore, the sequence RJ-EPI_ISL_492035|2020-05-29 also presented a slight change in the structure of SL4 (Fig. 5).

### Phylogenetical reconstruction of SARS-CoV-2 genome sequences reveals geographic localization influence

**Fig. 6.**
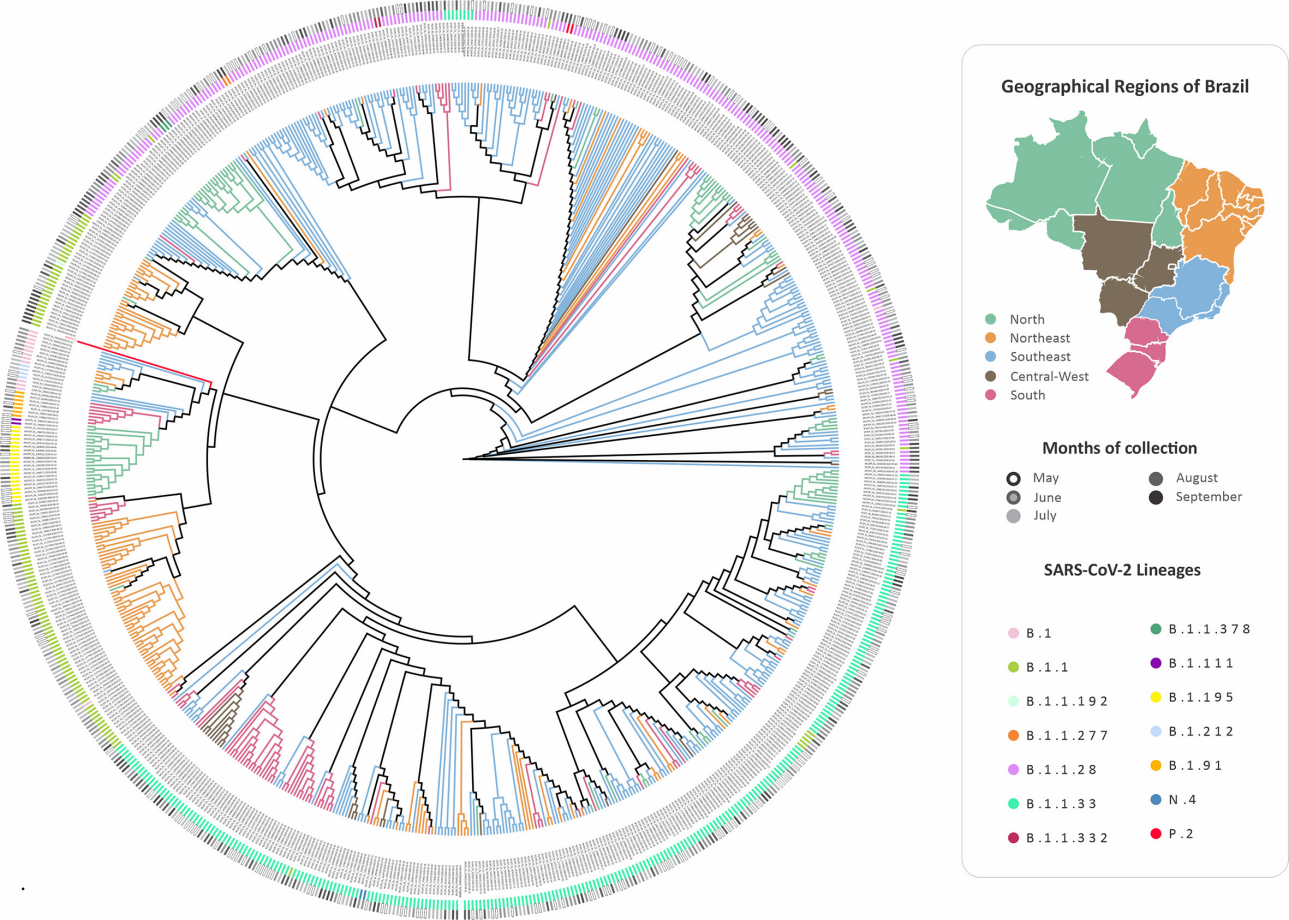
Phylogenetic tree reconstructed with 720 genome sequences with a high coverage (>75 %) from the GISAID database, related to samples collected in Brazil in the period from May to September, 2020, available on the database on 15 March 2021.The colour of the inner branches represents the geographic region of origin, also pointed to on the map. The different lineages and sublineages are marked as the full dots, and the circles indicate the month of the collection of samples. The lineages of the sequences were confirmed by Pangolin COVID-19 Lineage Assigner.

Phylogenetic tree analysis shows that sequences from the southeast are grouped to the reference sequence, isolated in Huwan, PR China. We identified that most sequences from samples of the same Brazilian state clustered and were closely related to the collection date (Fig. 6). We also observed that some sequences from the Southeast are grouped in the same cluster of sequences from the Northeast and the South. As shown in Fig. 6, sublineages B.1.1.28 and B.1.1.33 are the most prevalent.

## Discussion

Despite the prevalence of SARS-CoV-2 in Brazil [50, 51], few studies have reported general data on the genomic diversity of this virus, associating with epidemiological features. Most of them are focusing on reporting possible new VOIs or VOCs [22, 27, 52], or describing variants identified in specific regions or states of Brazil [53, 54]. The lack of epidemiological studies can affect the ongoing pandemic [55, 56], and amino acid substitutions resulted of mutations in the viral genome can result in more virulent or infectious strains of the SARS-CoV-2 [52]. According to De Souza and coworkers, details of its potential transmission, and clinical and epidemiological characteristics, remain poorly understood in the ongoing COVID-19 pandemic in Brazil [26]. Here we evaluated SARS-CoV-2 genome sequences from samples collected in Brazil between May and September of 2020 available up to 15 March 2021, and analysed relevant amino acid substitution and the selection pressure on viral genome sites, as well as potential viral genomic signatures.

Our findings described a higher SARS-CoV-2 infection prevalence in individuals between 36 and 54 years in the analysed period, which is in agreement with data from other countries [57]. However, information regarding the patient status were available for only 356 sequences, demonstrating a restricted data about infected people in Brazil. The lack of updates in databases with information on Brazilian infected patients is a strong point to be highlighted here, since over 50.1 % of the reports on gender and 50.7 % on status of infected patients is unknown among the available data. It implies difficulties in epidemiological analyses and clinical characterization of the pandemic around the world [55, 58, 59]. In this context, we suggest and emphasize the need of complete epidemiological data to produce reliable knowledge on the COVID-19 pandemic to assist the implementation of effective measures to mitigate the problem.

The B.1 lineage was associated with outbreaks in Italy and other European countries, which resulted in its spread worldwide, becoming the predominant global lineage [60]. Our results are in agreement with Rambaut and collaborators [60], since the presence of SARS-CoV-2 B lineages overlapped the sequences classified as lineage A, although its prevalence were already low in the Feb–April dataset (Fig. 1d) [24, 60]. Among the lineages B, B.1.1.28, and B.1.1.33 variants possessed the higher prevalence from May to September 2020, as in the previously period. These findings imply the prevalence and maintenance of B lineages in Brazil up to September 2020. Considering that the first introduction of the SARS-CoV-2 in Brazil was originating from Italy [61], and the number of COVID-19 cases in Brazil increased after the Italian outbreak, our findings on the prevalence of strains B.1.1.28 and B1.1.33 are in accordance with the literature, reinforcing the international introduction of SARS-CoV-2 in Brazil, causing its spread throughout the country due to the mitigation of the isolation measures [24]. We also identified sequences of P.2 lineage in 0.2% of the analysed samples (Fig. 1d), confirming the circulation of this variant in the period shortly after its identification, in April 2020 [62].

As previously described, the first case of SARS-CoV-2 in Brazil was detected in the southeast, in the state of São Paulo [61]. The similarity between the Wuhan reference and Southeast sequences supports this data [24, 61]. Additionally, taking into consideration the proximity, easy access, and the interval among collection dates, we identified clusters of sequences from Southeast and Northeast, which may suggest a possible route of transmission between these two regions. These routes of transmission were previously described between metropolitan centres as well as from capital to inland cities, triggering new strains/lineages introductions and an increase in transmissibility and mortality [63], which might suggest the easy dispersal of SARS-CoV-2 in Brazil, and the established route. Furthermore, clustering of sequences from the Southeast with the other Brazilian regions might indicate that, at some point during the pandemic in Brazil, the viral strain, which previously circulated mostly in state of São Paulo (Southeast), spread to other urban centres in the country, possibly due to the flexibilization of social isolation measures in Brazil [24]. Such flexibilization also resulted in the increase of SARS-CoV-2 transmission in other countries, mainly among patients with travel history, and recently, were associated with possible new waves of infection [64–66].

Our analysis identified amino acid substitutions related to the genomic regions that express several proteins such as Nsp7, RdRp, Spike, ORF6, and N. However, the amino acid substitution might not be sufficient to predict the impact on the proteins. We employed the FUBAR Bayesian algorithm and identified spots with positive (d*N* – nonsynonymous mutation) and negative (d*S* – synonymous mutation) pressures. Results demonstrated that the structural proteins S and N were under a stronger positive pressure when compared with other proteins such as nsP1, nsP2, and nsP16. This fact might be associated with the protein localization in the virion structure, since exposed proteins normally are known for receiving a major environment pressure than the less exposed proteins [67–69]. Additionally, the mutation on the position V1176F in the S protein was identified here in 41 % of the sequences from May to September, being also detected in the VOI P.2, identified in Brazil [62]. The total length of the SARS-CoV-2 S protein is 1273 aa with two subunits: S1 (N-terminal, 14–685 residues) and S2 (C-terminal, 686–1273 residues), which are responsible for receptor binding and membrane fusion, respectively [20]. The substitution V>F in the position 1176 alters the heptapeptide repeat sequence 2 (HR2; 1163–1213 residues), that forms a six-helical bundle (6-HB) with HR1 (912–984 residues), and this complex is essential for the viral fusion and entry function of the S2 subunit [20, 21]. Our results demonstrated that the substitution V1176F is under positive selection, suggesting under these circumstances that it is a P.2 key element of this variant that emerged from Brazil during the period of analysis. According to the function of 6-HB complex, the mutation might be related to a more efficient viral fusion and entry. Additionally, the N protein also showed positive selection pressure in the sites S202C/I and G204R, and the P.2 variant possessed the G204R mutation associated with R203K, which were observed in our data, however, without positive or negative selection. Moreover, the variants of concern B.1.351, B.1.427, and B.1.429 feature the T205I substitution, which were identified in 0.69% of our sequences from May to September (Table S1) [62]. In this context, we suggest that selection favours diversity at the sites 203 and 205 of the N protein. This is commonly seen in several viral proteins, as in Chikungunya virus, Hepatitis C virus, Lentivirus, and Foot and Mouth Disease virus, for example [70–73].

Another point to take into consideration is that the substitution D614G in the Spike protein was identified in almost 100 % of Brazilian sequences, in both periods, and, according to Zhang and coworkers, it replaced the D614-carrying virus becoming the dominant circulating strain worldwide [74]. The G614 variant was described *in vitro* and *in vivo* enhancing the SARS-CoV-2 replication on human lung epithelial cells and primary human airway tissues, and improving the infectivity of virions with the spike receptor-binding domain by a conformation upgrade for binding to ACE2 receptor [75]. There are several sites with positive selection identified in our analysis (Table S2), however, considering the analysed periods and the data regarding the current prevalent lineages of SARS-CoV-2, it is possible to hypothesize that none of these were effective spread and/or were not maintained in SARS-CoV-2 variants circulating in Brazil [76]. Recently, Castonguay and coworkers described natural upward and downward fluctuation in mutation prevalence in genome sequences from December 2019 to January 2021 in all VOCs, which is in agreement with our finding about maintenance of specific mutations [77]. The fitness costs might be a possible explanation for the lack of persistence of the mutations described here. The fitness values are environment-dependent, and the emergence of new mutations not always results in positive characteristics such as adaptability to the host, higher transmissibility and/or immune escape [78]. Some of them might cause deficient replication as well as facilitate the immune system recognition. Therefore, controlling the fitness costs will be critical to predict the persistence of the acquired mutations [79]. This can be evidenced by the emergence of the variants P.3 and P.4 in the Philippines and Mexico, respectively, which possess mutations that were identified in the P.2 variant, such as the V1176F [80, 81]. As was identified here, the presence of this mutation in late 2020 pointed out an increase in 119.2 % prevalence in V1176F, in contrast with the initial months of the pandemic in Brazil, indicating the persistence of this specific mutation possibly to guarantee viral adaptation. It is also important to highlight that with the available data it is not possible to confirm if the fitness cost is the only cause or if there are other factors operating in the mutation persistence of SARS-CoV-2 genomes. However, it demonstrates that actively epidemiological analyses on upward and downward fluctuations in mutations is critical, and if these analyses could be constantly updated with the sequences from past periods, we would be aware of the potential emergence of VOCs.

The high conservation of the 3′ and 5′ UTR structures is well established in previous studies [49, 82], which points out the importance of UTRs controlling the gene expression, both in replication and in transcription [83]. The secondary structures that constitute the SL1, SL2, SL5a, SL5b, and SL5c loopings of the 5′ UTR are directly associated with viral replication and packaging, suggesting that changes in these regions may compromise the entire replication cycle of the virus, as well as changes in the secondary structures that form the SL1, SL2, and SL3 structures of the 3′ UTR, that probably interact with other cellular factors, such as miRNA [51, 84, 85]. In this context, the investigation of these regions is needed and important to establish replication and virulence features to be associated with mutations in non-structural and structural proteins. Our results showed alteration of the secondary structures of the 5′ UTR and 3′ UTR in two and three sequences, respectively, agreeing with previous data about the conservation of these regions in order to guarantee an efficacious replication [49, 86]. Additionally, although the nucleotide substitution C>T at position 241 in the 5′ UTR was observed in all analysed sequences, its frequency is lower in the rest of the globe, and did not impact in the secondary structure of 5′ UTR [84]. Altogether, these results suggest that the emergence of new variants were not influenced by mutations in UTR regions, since it maintained its conformational structure in most analysed sequences. However, the association of these alterations with clinical status of infected patients is challenging, emphasizing the importance of studies regarding this subject, since these highly conserved regions represent strong candidates to therapeutic targets [57, 84].

Concluding, this work described the scenario of SARS-CoV-2 strains that have circulated in Brazil, and thus provide, with relevant information, the potential viral changes that may have affected and contributed to the current scenario of the COVID-19 pandemic.

## Supplementary Data

Supplementary material 1

Supplementary material 2
