# Supplementary material for: Insights on the SARS-CoV-2 genome variability: the lesson learned in Brazil and its impacts on the future of pandemics
Source: Microb Genom. 2021 Nov 3;7(11):000656. doi: 10.1099/mgen.0.000656 (PMC8743548; doi:10.1099/mgen.0.000656)
Supplement: Supplementary material 1 [file mgen-7-0656-s001.pdf]

Supplementary table 1: Selective pressure analysis across the genes of SARS-CoV-2 sequences using Fast Unconstrained Bayesian AppRoximation (FUBAR) with substitutions under episodic positive and negative selections.

Fast Unconstrained Bayesian AppRoximation (FUBAR) with substitutions under episodic positive selection

Protein	Site	α	β	Probability α > β	Probability α < β	BayesFactor
nsP2	555	0,754	11,149	0,011	0,974	44,546
nsP3	1439	1,467	20,129	0,015	0,960	36,206
	1442	1,54	10,843	0,036	0,920	17,110
	1244	1,538	10,708	0,037	0,919	16,830
	822	1,528	10,075	0,039	0,914	15,905
nsP4	384	1,996	19,07	0,039	0,930	16,316
nsP5	220	0,914	12,088	0,037	0,941	20,252
nsP6	149	1,797	12,539	0,059	0,903	11,662
	194	1,796	12,525	0,060	0,903	11,658
nsP14	285	0,874	10,751	0,042	0,933	17,765
nsP15	284	3,682	29,802	0,037	0,928	15,818
	289	1,72	13,502	0,054	0,913	12,889
	91	2,263	15,003	0,060	0,904	11,564
nsP16	213	0,918	19,519	0,005	0,984	81,388
Orf3a	224	0,764	10,534	0,003	0,984	69,130
	240	0,754	6,053	0,032	0,938	17,471
RdRp 2ª frameshift	102	1,239	21,018	0,025	0,953	28,999
	334	3,644	26,199	0,037	0,922	16,762
	256	1,799	15,93	0,052	0,912	14,625
	71	2,937	20,99	0,055	0,903	13,208
Spike	1176	1,605	47,187	0,000	0,996	390,068
	614	1,337	23,199	0,005	0,982	82,382
	262	1,303	18,012	0,008	0,972	53,170
	67	1,299	17,844	0,009	0,972	52,738
	75	1,264	12,18	0,023	0,947	27,074
	565	1,641	10,013	0,042	0,917	16,727
Helicase	505	2,052	15,52	0,055	0,909	13,347
M	94	1,171	11,155	0,053	0,918	14,340
N	292	2,754	44,212	0,000	0,999	801,651
	193	0,849	10,607	0,013	0,967	35,088
	202	0,847	10,52	0,013	0,967	34,855
	204	1,695	17,731	0,019	0,956	25,389
	12	1,816	9,762	0,044	0,917	13,083
	24	0,707	7,787	0,049	0,926	14,800

Fast Unconstrained Bayesian AppRoximation (FUBAR) with substitutions under episodic negative/purifying selection

Protein	Site	α	β	Probability α > β	Probability α < β	BayesFactor
nsP1	59	12,847	1,352	0,931	0,041	0,050
	88	19,141	1,155	0,964	0,019	0,023
	96	25,286	1,460	0,926	0,054	0,067
nsp2	301	16,442	0,663	0,974	0,016	0,019
	446	7,607	0,623	0,939	0,041	0,051
	436	7,673	0,700	0,931	0,045	0,056
nsp3	1249	44,994	1,452	0,998	0,000	0,000
	236	27,692	0,745	0,991	0,005	0,002
	1200	45,373	8,495	0,987	0,001	0,007
	205	22,520	0,828	0,983	0,008	0,008
	825	12,149	0,856	0,980	0,006	0,012
	1700	7,370	0,680	0,948	0,031	0,047
	1117	7,433	0,742	0,943	0,033	0,049
	1197	7,804	0,767	0,943	0,032	0,051
nsp4	496	35,113	0,882	0,994	0,003	0,003
	207	21,467	1,243	0,910	0,067	0,089
	214	18,575	0,836	0,909	0,071	0,093
	240	19,873	1,151	0,904	0,073	0,096
nsp6	270	32,630	1,183	0,988	0,005	0,006
nsP8	162	19,125	0,773	0,922	0,061	0,082
	111	19,129	0,876	0,917	0,064	0,086
	36	19,927	1,207	0,910	0,068	0,092
	99	18,287	0,987	0,907	0,071	0,097
nsP9	92	25,977	1,257	0,936	0,048	0,061
	14	19,588	0,811	0,917	0,065	0,083
	66	18,674	0,925	0,907	0,072	0,093
nsP10	13	23,292	0,819	0,936	0,049	0,063
	11	23,297	0,848	0,935	0,050	0,064
	93	25,520	1,190	0,935	0,048	0,062
nsp14	35	22,469	0,871	0,981	0,010	0,013
	71	11,755	1,290	0,933	0,040	0,053
	310	21,404	1,025	0,921	0,060	0,081
	5	19,002	0,858	0,915	0,065	0,089
	77	21,536	1,417	0,911	0,066	0,090
	232	19,062	1,064	0,908	0,070	0,096
nsP15	84	18,176	0,795	0,976	0,013	0,017
	145	22,385	1,126	0,923	0,058	0,076
	266	22,385	1,126	0,923	0,058	0,076
	67	18,962	0,701	0,922	0,061	0,080
	23	19,108	0,701	0,923	0,061	0,079
	268	16,423	0,716	0,907	0,073	0,097

Fast Unconstrained Bayesian AppRoximation (FUBAR) with substitutions under episodic negative/purifying selection

Protein	Site	α	β	Probability α > β	Probability α < β	BayesFactor
nsP16	199	19,530	0,719	0,983	0,010	0,013
	94	10,842	0,893	0,946	0,033	0,045
	70	17,424	0,709	0,918	0,064	0,090
	250	17,431	0,709	0,918	0,064	0,090
	99	17,457	0,794	0,913	0,067	0,095
	222	17,480	0,860	0,911	0,069	0,097
Orf3a	84	6,262	0,678	0,953	0,024	0,029
	62	6,730	0,624	0,926	0,051	0,062
	131	25,766	1,065	0,905	0,075	0,092
Orf7a	79	12,905	0,685	0,962	0,024	0,027
	85	23,237	0,971	0,924	0,058	0,068
Orf8	99	10,619	0,459	0,953	0,034	0,037
	53	21,749	0,463	0,941	0,046	0,052
RdRp 2ª frameshift	477	15,992	1,338	0,957	0,022	0,032
	92	17,375	0,787	0,922	0,059	0,089
	481	17,733	0,867	0,921	0,060	0,090
	329	18,283	1,074	0,916	0,062	0,094
	321	17,523	0,944	0,917	0,062	0,094
	452	17,533	0,944	0,917	0,062	0,094
	876	17,499	0,950	0,916	0,063	0,095
	350	19,163	1,445	0,910	0,065	0,098
	782	15,472	1,094	0,900	0,074	0,114
Spike	263	39,259	2,986	0,990	0,001	0,002
	324	32,797	1,033	0,992	0,003	0,005
	163	10,737	0,652	0,969	0,018	0,028
	606	10,579	0,822	0,958	0,024	0,037
	180	15,236	0,877	0,910	0,068	0,111
	191	15,219	0,876	0,910	0,068	0,111
	340	15,236	0,877	0,910	0,068	0,111
	386	15,216	0,898	0,909	0,069	0,112
	1211	15,251	0,901	0,909	0,069	0,112
	564	15,281	0,950	0,907	0,070	0,114
	1263	11,221	0,643	0,902	0,075	0,123
	829	11,230	0,655	0,901	0,076	0,125
	1030	11,284	0,667	0,901	0,076	0,125
Helicase	510	17,925	0,873	0,976	0,013	0,017
	356	16,658	0,867	0,973	0,015	0,020
	493	24,211	1,018	0,940	0,045	0,063
	418	23,113	1,688	0,920	0,057	0,080
	399	16,013	0,915	0,904	0,073	0,105
	306	16,288	1,054	0,901	0,076	0,109
M	41	19,416	0,839	0,978	0,012	0,016
	135	17,667	0,852	0,911	0,069	0,095

Fast Unconstrained Bayesian AppRoximation (FUBAR) with substitutions under episodic negative/purifying selection

Protein	Site	α	β	Probability α > β	Probability α < β	BayesFactor
	191	14,568	0,701	0,902	0,077	0,107
N	337	12,716	0,590	0,990	0,004	0,005
	346	7,607	0,498	0,952	0,034	0,041
	35	9,147	0,633	0,948	0,035	0,042
	16	9,105	0,719	0,940	0,039	0,048
	353	4,735	0,593	0,903	0,069	0,087

Supplementary table 3: GISAID available data from the sequences used in this study, including virus name; accession ID; collection date; location; host passage; specimen; additional host information; sequencing technology; assembly method; type lineage.

Virus name Accession ID Collection date Location Host Passage
Specimen Additional host information Sequencing technology
Assembly method Comment Comment type Lineage Clade
hCoV-19/Brazil/SP-1126/2020 EPI_ISL_1039696 2020-08-16 South America / Brazil / Sao Paulo / Presidente Prudente Human Original
Nasopharyngeal swab Ion Torrent S5 IRMA
B.1.1.28 GR
hCoV-19/Brazil/SP-1127/2020 EPI_ISL_1039697 2020-09-26 South America / Brazil / Sao Paulo / Mogi das Cruzes Human Original Nasopharyngeal swab Ion Torrent S5 IRMA B.1.1.28 GR
hCoV-19/Brazil/SP-1128/2020 EPI_ISL_1039698 2020-09-29 South America / Brazil / Sao Paulo / Taboao da Serra Human Original Nasopharyngeal swab Ion Torrent S5 IRMA B.1.1.28 GR
hCoV-19/Brazil/SP-1131/2020 EPI_ISL_1039699 2020-09-28 South America / Brazil / Sao Paulo / Pindamonhangaba Human Original Nasopharyngeal swab Ion Torrent S5 IRMA B.1.1.28 GR
hCoV-19/Brazil/SP-1133/2020 EPI_ISL_1039700 2020-09-28 South America / Brazil / Sao Paulo / Mairipora Human Original Nasopharyngeal swab
Ion Torrent S5 IRMA B.1.1.33 GR
hCoV-19/Brazil/SP-1134/2020 EPI_ISL_1039701 2020-09-25 South America / Brazil / Sao Paulo / Cajati Human Original Nasopharyngeal swab
Ion Torrent S5 IRMA B.1.1.28 GR
hCoV-19/Brazil/SP-1135/2020 EPI_ISL_1039702 2020-08-28 South America / Brazil / Sao Paulo / Andradina Human Original Nasopharyngeal swab
Ion Torrent S5 IRMA B.1.1.28 GR
hCoV-19/Brazil/MS-1259/2020 EPI_ISL_1040833 2020-09-20 South America / Brazil / Mato Grosso do Sul / Agua Clara Human Original
Nasopharyngeal swab Ion Torrent S5 IRMA
B.1.1.33 GR
hCoV-19/Brazil/MS-1260/2020 EPI_ISL_1040834 2020-09-19 South America / Brazil / Mato Grosso do Sul / Dourados Human Original Nasopharyngeal swab Ion Torrent S5 IRMA B.1.1.28 GR
hCoV-19/Brazil/MS-1261/2020 EPI_ISL_1040835 2020-09-19 South America / Brazil / Mato Grosso do Sul / Campo Grande Human Original
Nasopharyngeal swab Ion Torrent S5 IRMA
B.1.1.33 GR
hCoV-19/Brazil/MS-1262/2020 EPI_ISL_1040836 2020-09-18 South America / Brazil / Mato Grosso do Sul / Campo Grande Human Original
Nasopharyngeal swab Ion Torrent S5 IRMA
B.1.1.33 GR
hCoV-19/Brazil/MS-1263/2020 EPI_ISL_1040837 2020-09-18 South America / Brazil / Mato Grosso do Sul / Ladario Human Original Nasopharyngeal swab Ion Torrent S5 IRMA B.1.1.33 GR
hCoV-19/Brazil/MS-1264/2020 EPI_ISL_1040838 2020-09-18 South America / Brazil / Mato Grosso do Sul / Ponta Pora Human Original
Nasopharyngeal swab Ion Torrent S5 IRMA
B.1.1.28 GR
hCoV-19/Brazil/MS-1265/2020 EPI_ISL_1040839 2020-09-16 South America / Brazil / Mato Grosso do Sul / Anastacio Human Original Nasopharyngeal swab Ion Torrent S5 IRMA B.1.1.33 GR
hCoV-19/Brazil/MS-1266/2020 EPI_ISL_1040840 2020-09-16 South America / Brazil / Mato Grosso do Sul / Coxim Human Original Nasopharyngeal swab Ion Torrent S5 IRMA B.1.1.33 GR
hCoV-19/Brazil/MS-1268/2020 EPI_ISL_1040841 2020-08-28 South America / Brazil / Mato Grosso do Sul / Rondonopolis Human Original
Nasopharyngeal swab Ion Torrent S5 IRMA
B.1.1.28 GR

hCoV-19/Brazil/MS-1267/2020 EPI_ISL_1040842 2020-08-27 South America / Brazil / Mato Grosso do Sul / Sonora Human Original Nasopharyngeal swab Ion Torrent S5 IRMA B.1.1.33 GR
hCoV-19/Brazil/MS-1269/2020 EPI_ISL_1040843 2020-08-27 South America / Brazil / Mato Grosso do Sul / Figueirao Human Original Nasopharyngeal swab Ion Torrent S5 IRMA B.1.1.33 GR
hCoV-19/Brazil/MS-1274/2020 EPI_ISL_1040844 2020-08-18 South America / Brazil / Mato Grosso do Sul / Selviria Human Original Nasopharyngeal swab Ion Torrent S5 IRMA B.1.1.33 GR
hCoV-19/Brazil/MS-1275/2020 EPI_ISL_1040845 2020-08-18 South America / Brazil / Mato Grosso do Sul / Tres Lagoas Human Original Nasopharyngeal swab Ion Torrent S5 IRMA B.1.1.33 GR
hCoV-19/Brazil/MS-1273/2020 EPI_ISL_1040846 2020-08-17 South America / Brazil / Mato Grosso do Sul / Dourados Human Original Nasopharyngeal swab Ion Torrent S5 IRMA B.1.1.28 GR
hCoV-19/Brazil/MS-1271/2020 EPI_ISL_1040847 2020-07-31 South America / Brazil / Mato Grosso do Sul / Campo Grande Human Original Nasopharyngeal swab Ion Torrent S5 IRMA B.1.1.28 GR
hCoV-19/Brazil/MS-1272/2020 EPI_ISL_1040848 2020-07-31 South America / Brazil / Mato Grosso do Sul / Campo Grande Human Original Nasopharyngeal swab Ion Torrent S5 IRMA B.1.1.33 GR
hCoV-19/Brazil/MS-1279/2020 EPI_ISL_1040849 2020-07-23 South America / Brazil / Mato Grosso do Sul / Jardim Human Original Nasopharyngeal swab Ion Torrent S5 IRMA B.1.1.28 GR
hCoV-19/Brazil/MS-1277/2020 EPI_ISL_1040850 2020-07-22 South America / Brazil / Mato Grosso do Sul / Campo Grande Human Original Nasopharyngeal swab Ion Torrent S5 IRMA B.1.1.28 GR
hCoV-19/Brazil/MS-1278/2020 EPI_ISL_1040851 2020-07-22 South America / Brazil / Mato Grosso do Sul / Iguatemi Human Original Nasopharyngeal swab Ion Torrent S5 IRMA B.1.1.33 GR
hCoV-19/Brazil/AM-L87-CD2693/2020 EPI_ISL_1061031 2020-05-27 South America / Brazil / Amazonas / Manaus Human Original Nasopharyngeal and oropharyngeal swab Nanopore minION Artic-nCoV Gap of 9 nucleotides when compared to the reference sequence. info B.1.195 G
hCoV-19/Brazil/PA-IEC169757/2020 EPI_ISL_1063789 2020-06-23 South America / Brazil / Para Human Original Nasopharyngeal swab Illumina NextSeq MEGAHIT v1.2.9 / Geneious Prim B.1.1.28 GR
hCoV-19/Brazil/AM-20141355VB/2020 EPI_ISL_1068089 2020-08-18 South America / Brazil / Amazonas / Manaus Human Original Oropharyngeal swab Illumina MiSeq BBMap 37.25 embedded in Geneious 10.2.14 B.1.1.28 GR
hCoV-19/Brazil/AM-20141399MC/2020 EPI_ISL_1068090 2020-08-28 South America / Brazil / Amazonas / Manaus Human Original Oropharyngeal swab Illumina MiSeq BBMap 37.25 embedded in Geneious 10.2.15 B.1.1.28 GR
hCoV-19/Brazil/AM-20141466MD/2020 EPI_ISL_1068091 2020-08-06 South America / Brazil / Amazonas / Lábrea Human Original Oropharyngeal swab Illumina MiSeq BBMap 37.25 embedded in Geneious 10.2.16 Gap of 9 nucleotides when compared to the reference sequence. info B.1.195 G
hCoV-19/Brazil/AM-20141493LR/2020 EPI_ISL_1068092 2020-08-05 South America / Brazil / Amazonas / Lábrea Human Original Oropharyngeal

swab	Illumina MiSeq	BBMap 37.25 embedded in Geneious 10.2.17	Gap of 9 nucleotides when compared to the reference sequence. info	B.1.195	G
hCoV-19/Brazil/AM-20141562YS/2020 EPI_ISL_1068093	2020-09-01	South America / Brazil / Amazonas / Manaus	Human Original	Oropharyngeal swab	Illumina MiSeq	BBMap 37.25 embedded in Geneious 10.2.18	B.1.1.28	GR
hCoV-19/Brazil/AM-20141582LB/2020 EPI_ISL_1068094	2020-09-04	South America / Brazil / Amazonas / Manaus	Human Original	Oropharyngeal swab	Illumina MiSeq	BBMap 37.25 embedded in Geneious 10.2.19	B.1.1.33	GR
hCoV-19/Brazil/AM-20141671CB/2020 EPI_ISL_1068095	2020-09-15	South America / Brazil / Amazonas / Manaus	Human Original	Oropharyngeal swab	Illumina MiSeq	BBMap 37.25 embedded in Geneious 10.2.20	B.1.1.28	GR
hCoV-19/Brazil/AM-20141721RM/2020 EPI_ISL_1068096	2020-09-17	South America / Brazil / Amazonas / Manaus	Human Original	Oropharyngeal swab	Illumina MiSeq	BBMap 37.25 embedded in Geneious 10.2.21	B.1.1.28	GR
hCoV-19/Brazil/AM-20141735HN/2020 EPI_ISL_1068097	2020-09-18	South America / Brazil / Amazonas / Manaus	Human Original	Oropharyngeal swab	Illumina MiSeq	BBMap 37.25 embedded in Geneious 10.2.22	B.1.1.33	GR
hCoV-19/Brazil/AM-20141881JO/2020 EPI_ISL_1068098	2020-09-24	South America / Brazil / Amazonas / Manaus	Human Original	Oropharyngeal swab	Illumina MiSeq	BBMap 37.25 embedded in Geneious 10.2.23	B.1.1.33	GR
hCoV-19/Brazil/AM-20142002AL/2020 EPI_ISL_1068099	2020-09-29	South America / Brazil / Amazonas / Manaus	Human Original	Oropharyngeal swab	Illumina MiSeq	BBMap 37.25 embedded in Geneious 10.2.25	B.1.1.33	GR
hCoV-19/Brazil/AM-20142019FJ/2020 EPI_ISL_1068100	2020-09-24	South America / Brazil / Amazonas / Manaus	Human Original	Oropharyngeal swab	Illumina MiSeq	BBMap 37.25 embedded in Geneious 10.2.26	B.1.1.28	GR
hCoV-19/Brazil/AM-20841585PM/2020 EPI_ISL_1068120	2020-09-02	South America / Brazil / Amazonas / Manaus	Human Original	Oropharyngeal swab	Illumina MiSeq	BBMap 37.25 embedded in Geneious 10.2.46	B.1.1.33	GR
hCoV-19/Brazil/AM-20841592RS/2020 EPI_ISL_1068121	2020-09-02	South America / Brazil / Amazonas / Manaus	Human Original	Oropharyngeal swab	Illumina MiSeq	BBMap 37.25 embedded in Geneious 10.2.47	B.1.1.33	GR
hCoV-19/Brazil/AM-20841891TC/2020 EPI_ISL_1068122	2020-09-17	South America / Brazil / Amazonas / Manaus	Human Original	Oropharyngeal swab	Illumina MiSeq	BBMap 37.25 embedded in Geneious 10.2.48	B.1.1.33	GR
hCoV-19/Brazil/AM-20890046AA/2020 EPI_ISL_1068168	2020-05-17	South America / Brazil / Amazonas / Manicoré	Human Original	Oropharyngeal swab	Illumina MiSeq	BBMap 37.25 embedded in Geneious 10.2.95	B.1.1.28	GR
hCoV-19/Brazil/AM-20890068AA/2020 EPI_ISL_1068170	2020-05-03	South America / Brazil / Amazonas / Nova Olinda do Norte	Human Original	Oropharyngeal swab	Illumina MiSeq	BBMap 37.25 embedded in Geneious 10.2.97	Gap of 9 nucleotides when compared to the reference sequence.	info	B.1.195	G
hCoV-19/Brazil/AM-20890079MS/2020 EPI_ISL_1068171	2020-06-06	South America / Brazil / Amazonas / Manicoré	Human Original	Oropharyngeal

swab	Illumina MiSeq	BBMap 37.25 embedded in Geneious 10.2.98	Gap of 9 nucleotides when compared to the reference sequence. info	B.1.195	G
hCoV-19/Brazil/AM-20890083OP/2020	EPI_ISL_1068172	2020-06-06	South America / Brazil / Amazonas / Manicoré	Human	Original	Oropharyngeal swab	Illumina MiSeq	BBMap 37.25 embedded in Geneious 10.2.99	B.1.1.289	GR
hCoV-19/Brazil/AM-20890084SS/2020	EPI_ISL_1068173	2020-06-08	South America / Brazil / Amazonas / Manicoré	Human	Original	Oropharyngeal swab	Illumina MiSeq	BBMap 37.25 embedded in Geneious 10.2.100	Gap of 9 nucleotides when compared to the reference sequence. info	B.1.195	G
hCoV-19/Brazil/AM-20890090MA/2020	EPI_ISL_1068174	2020-05-18	South America / Brazil / Amazonas / Manaquiri	Human	Original	Oropharyngeal swab	Illumina MiSeq	BBMap 37.25 embedded in Geneious 10.2.101	Gap of 9 nucleotides when compared to the reference sequence. info	B.1.195	G
hCoV-19/Brazil/AM-20890091VS/2020	EPI_ISL_1068175	2020-05-17	South America / Brazil / Amazonas / Manaquiri	Human	Original	Oropharyngeal swab	Illumina MiSeq	BBMap 37.25 embedded in Geneious 10.2.102	B.1.1.28	GR
hCoV-19/Brazil/AM-20890125FV/2020	EPI_ISL_1068177	2020-05-18	South America / Brazil / Amazonas / Manaus	Human	Original	Oropharyngeal swab	Illumina MiSeq	BBMap 37.25 embedded in Geneious 10.2.104	Gap of 9 nucleotides when compared to the reference sequence. info	B.1.195	G
hCoV-19/Brazil/AM-20890140CD/2020	EPI_ISL_1068180	2020-06-10	South America / Brazil / Amazonas / Santa Isabel do Rio Negro	Human	Original	Oropharyngeal swab	Illumina MiSeq	BBMap 37.25 embedded in Geneious 10.2.107	Gap of 9 nucleotides when compared to the reference sequence.	info	B.1.195	G
hCoV-19/Brazil/AM-20890173FB/2020	EPI_ISL_1068182	2020-06-09	South America / Brazil / Amazonas / Manaquiri	Human	Original	Oropharyngeal swab	Illumina MiSeq	BBMap 37.25 embedded in Geneious 10.2.109	B.1.1.28	GR
hCoV-19/Brazil/AM-20890235AC/2020	EPI_ISL_1068187	2020-05-02	South America / Brazil / Amazonas / Presidente Figueiredo	Human	Original	Oropharyngeal swab	Illumina MiSeq	BBMap 37.25 embedded in Geneious 10.2.114	Gap of 9 nucleotides when compared to the reference sequence.	info	B.1.195	G
hCoV-19/Brazil/AM-20890244FP/2020	EPI_ISL_1068188	2020-05-03	South America / Brazil / Amazonas / Parintins	Human	Original	Oropharyngeal swab	Illumina MiSeq	BBMap 37.25 embedded in Geneious 10.2.115	Gap of 9 nucleotides when compared to the reference sequence. info	B.1.195	G
hCoV-19/Brazil/AM-20890295AM/2020	EPI_ISL_1068189	2020-07-27	South America / Brazil / Amazonas / Manaus	Human	Original	Oropharyngeal swab	Illumina MiSeq	BBMap 37.25 embedded in Geneious 10.2.116	B.1.1.33	GR
hCoV-19/Brazil/AM-20890297AX/2020	EPI_ISL_1068190	2020-07-25	South America / Brazil / Amazonas / Manaus	Human	Original	Oropharyngeal swab	Illumina MiSeq	BBMap 37.25 embedded in Geneious 10.2.117	Gap of 19 nucleotides when compared to the reference sequence.	info	B.1.1.28	GR
hCoV-19/Brazil/AM-20890702BS/2020	EPI_ISL_1068197	2020-05-24	South America / Brazil / Amazonas / Iranduba	Human	Original	Oropharyngeal swab	Illumina MiSeq	BBMap 37.25 embedded in Geneious 10.2.124	B.1.1.28	GR

hCoV-19/Brazil/AM-20891128MC/2020 EPI_ISL_1068201 2020-05-15 South America / Brazil / Amazonas / Tabatinga Human Original Oropharyngeal swab Illumina MiSeq BBMap 37.25 embedded in Geneious 10.2.129 Gap of 9 nucleotides when compared to the reference sequence. info B.1.111 GH
hCoV-19/Brazil/AM-20891184SB/2020 EPI_ISL_1068203 2020-05-18 South America / Brazil / Amazonas / Tabatinga Human Original Oropharyngeal swab Illumina MiSeq BBMap 37.25 embedded in Geneious 10.2.131 B.1.111 GH
hCoV-19/Brazil/AM-20891192AS/2020 EPI_ISL_1068204 2020-05-27 South America / Brazil / Amazonas / Careiro Human Original Oropharyngeal swab Illumina MiSeq BBMap 37.25 embedded in Geneious 10.2.132 Gap of 14 nucleotides when compared to the reference sequence. info B.1.1.33 GR
hCoV-19/Brazil/AM-20891452AC/2020 EPI_ISL_1068205 2020-07-12 South America / Brazil / Amazonas / Manaus Human Original Oropharyngeal swab Illumina MiSeq BBMap 37.25 embedded in Geneious 10.2.133 NS7a_Q90stop results in 26.4% truncation of the protein sequence. info B.1.1.28 GR
hCoV-19/Brazil/AM-20891505PT/2020 EPI_ISL_1068206 2020-07-23 South America / Brazil / Amazonas / Manaus Human Original Oropharyngeal swab Illumina MiSeq BBMap 37.25 embedded in Geneious 10.2.134 B.1.1.28 GR
hCoV-19/Brazil/AM-20891507CM/2020 EPI_ISL_1068207 2020-07-24 South America / Brazil / Amazonas / Manaus Human Original Oropharyngeal swab Illumina MiSeq BBMap 37.25 embedded in Geneious 10.2.135 Gap of 9 nucleotides when compared to the reference sequence. info B.1.195 G
hCoV-19/Brazil/AM-20891515LA/2020 EPI_ISL_1068208 2020-07-24 South America / Brazil / Amazonas / Manaus Human Original Oropharyngeal swab Illumina MiSeq BBMap 37.25 embedded in Geneious 10.2.136 B.1.1.28 GR
hCoV-19/Brazil/AM-20891550FA/2020 EPI_ISL_1068209 2020-07-29 South America / Brazil / Amazonas / Manaus Human Original Oropharyngeal swab Illumina MiSeq BBMap 37.25 embedded in Geneious 10.2.137 B.1.1.28 GR
hCoV-19/Brazil/AM-20891643FA/2020 EPI_ISL_1068211 2020-06-08 South America / Brazil / Amazonas / Careiro Human Original Oropharyngeal swab Illumina MiSeq BBMap 37.25 embedded in Geneious 10.2.139 B.1.1.28 GR
hCoV-19/Brazil/AM-20891666CS/2020 EPI_ISL_1068212 2020-05-23 South America / Brazil / Amazonas / Santo Antônio do Içá Human Original Oropharyngeal swab Illumina MiSeq BBMap 37.25 embedded in Geneious 10.2.140 Gap of 9 nucleotides when compared to the reference sequence. info B.1.195 G
hCoV-19/Brazil/AM-20891680JR/2020 EPI_ISL_1068213 2020-06-10 South America / Brazil / Amazonas / Manaquiri Human Original Oropharyngeal swab Illumina MiSeq BBMap 37.25 embedded in Geneious 10.2.141 B.1.1.28 GR
hCoV-19/Brazil/AM-20891688MN/2020 EPI_ISL_1068214 2020-06-09 South America / Brazil / Amazonas / Manaquiri Human Original Oropharyngeal swab Illumina MiSeq BBMap 37.25 embedded in Geneious 10.2.142 B.1.1.28 GR
hCoV-19/Brazil/AM-20891894JM/2020 EPI_ISL_1068216 2020-05-19 South America / Brazil / Amazonas / Manaus Human Original Oropharyngeal swab Illumina MiSeq BBMap 37.25 embedded in Geneious 10.2.144 B.1.1.33 GR

hCoV-19/Brazil/AM-20891898RL/2020 EPI_ISL_1068217 2020-05-19 South America / Brazil / Amazonas / Manaus Human Original Oropharyngeal swab Illumina MiSeq BBMap 37.25 embedded in Geneious 10.2.145 B.1.1.28 GR
hCoV-19/Brazil/AM-20891904JB/2020 EPI_ISL_1068218 2020-05-19 South America / Brazil / Amazonas / Autazes Human Original Oropharyngeal swab Illumina MiSeq BBMap 37.25 embedded in Geneious 10.2.146 B.1.1.28 GR
hCoV-19/Brazil/AM-20892241JS/2020 EPI_ISL_1068224 2020-06-23 South America / Brazil / Amazonas / Manaus Human Original Oropharyngeal swab Illumina MiSeq BBMap 37.25 embedded in Geneious 10.2.152 Gap of 9 nucleotides when compared to the reference sequence. info B.1.195 G
hCoV-19/Brazil/AM-20892300TV/2020 EPI_ISL_1068227 2020-06-29 South America / Brazil / Amazonas / Barreirinha Human Original Oropharyngeal swab Illumina MiSeq BBMap 37.25 embedded in Geneious 10.2.155 B.1.212 G
hCoV-19/Brazil/AM-20892477WL/2020 EPI_ISL_1068228 2020-07-27 South America / Brazil / Amazonas / Manaus Human Original Oropharyngeal swab Illumina MiSeq BBMap 37.25 embedded in Geneious 10.2.156 Gap of 29 nucleotides when compared to the reference sequence. info B.1.1.33 GR
hCoV-19/Brazil/AM-20892484AS/2020 EPI_ISL_1068229 2020-07-20 South America / Brazil / Amazonas / Manaus Human Original Oropharyngeal swab Illumina MiSeq BBMap 37.25 embedded in Geneious 10.2.157 B.1.1.33 GR
hCoV-19/Brazil/AM-20892494MS/2020 EPI_ISL_1068230 2020-07-20 South America / Brazil / Amazonas / Manaus Human Original Oropharyngeal swab Illumina MiSeq BBMap 37.25 embedded in Geneious 10.2.158 B.1.1.33 GR
hCoV-19/Brazil/AM-20892520WS/2020 EPI_ISL_1068231 2020-07-24 South America / Brazil / Amazonas / Manaus Human Original Oropharyngeal swab Illumina MiSeq BBMap 37.25 embedded in Geneious 10.2.159 Gap of 9 nucleotides when compared to the reference sequence. info B.1.195 G
hCoV-19/Brazil/AM-20892521AG/2020 EPI_ISL_1068232 2020-07-24 South America / Brazil / Amazonas / Manaus Human Original Oropharyngeal swab Illumina MiSeq BBMap 37.25 embedded in Geneious 10.2.160 Gap of 9 nucleotides when compared to the reference sequence. info B.1.195 G
hCoV-19/Brazil/AM-20892523EN/2020 EPI_ISL_1068233 2020-07-26 South America / Brazil / Amazonas / Iranduba Human Original Oropharyngeal swab Illumina MiSeq BBMap 37.25 embedded in Geneious 10.2.161 Gap of 9 nucleotides when compared to the reference sequence. info B.1.195 G
hCoV-19/Brazil/AM-20892533JB/2020 EPI_ISL_1068234 2020-07-28 South America / Brazil / Amazonas / Manaus Human Original Oropharyngeal swab Illumina MiSeq BBMap 37.25 embedded in Geneious 10.2.162 B.1.1.33 GR
hCoV-19/Brazil/AM-20892944LC/2020 EPI_ISL_1068235 2020-09-14 South America / Brazil / Amazonas / Manaus Human Original Oropharyngeal swab Illumina MiSeq BBMap 37.25 embedded in Geneious 10.2.163 B.1.1.31 GR
hCoV-19/Brazil/AM-20892962AF/2020 EPI_ISL_1068236 2020-09-15 South America / Brazil / Amazonas / Manaus Human Original Oropharyngeal swab Illumina MiSeq BBMap 37.25 embedded in Geneious 10.2.164 B.1.1.28 GR

hCoV-19/Brazil/AM-20892974ES/2020 EPI_ISL_1068237 2020-09-15 South America / Brazil / Amazonas / Manaus Human Original Oropharyngeal swab Illumina MiSeq BBMap 37.25 embedded in Geneious 10.2.165 Gap of 9 nucleotides when compared to the reference sequence. info B.1.195 G
hCoV-19/Brazil/AM-20892974MN/2020 EPI_ISL_1068238 2020-05-12 South America / Brazil / Amazonas / Manacapuru Human Original Oropharyngeal swab Illumina MiSeq BBMap 37.25 embedded in Geneious 10.2.166 Gap of 9 nucleotides when compared to the reference sequence. info B.1.195 G
hCoV-19/Brazil/AM-20893356IS/2020 EPI_ISL_1068239 2020-05-11 South America / Brazil / Amazonas / Manaus Human Original Oropharyngeal swab Illumina MiSeq BBMap 37.25 embedded in Geneious 10.2.167 Gap of 9 nucleotides when compared to the reference sequence. info B.1.195 G
hCoV-19/Brazil/AM-20893481LS/2020 EPI_ISL_1068240 2020-05-12 South America / Brazil / Amazonas / Manaus Human Original Oropharyngeal swab Illumina MiSeq BBMap 37.25 embedded in Geneious 10.2.168 B.1.1.33 GR
hCoV-19/Brazil/AM-20893844ED/2020 EPI_ISL_1068241 2020-05-21 South America / Brazil / Amazonas / Manacapuru Human Original Oropharyngeal swab Illumina MiSeq BBMap 37.25 embedded in Geneious 10.2.169 B.1.1.33 GR
hCoV-19/Brazil/AM-20893858ML/2020 EPI_ISL_1068242 2020-05-21 South America / Brazil / Amazonas / Manacapuru Human Original Oropharyngeal swab Illumina MiSeq BBMap 37.25 embedded in Geneious 10.2.170 B.1.1.28 GR
hCoV-19/Brazil/AM-20894176SC/2020 EPI_ISL_1068244 2020-05-26 South America / Brazil / Amazonas / Manacapuru Human Original Oropharyngeal swab Illumina MiSeq BBMap 37.25 embedded in Geneious 10.2.172 B.1.1.143 GR
hCoV-19/Brazil/AM-20894648MM/2020 EPI_ISL_1068245 2020-06-10 South America / Brazil / Amazonas / Manacapuru Human Original Oropharyngeal swab Illumina MiSeq BBMap 37.25 embedded in Geneious 10.2.173 B.1.1.28 GR
hCoV-19/Brazil/AM-20894654WF/2020 EPI_ISL_1068246 2020-06-11 South America / Brazil / Amazonas / Manacapuru Human Original Oropharyngeal swab Illumina MiSeq BBMap 37.25 embedded in Geneious 10.2.174 B.1.1.28 GR
hCoV-19/Brazil/AM-20894660LA/2020 EPI_ISL_1068247 2020-06-11 South America / Brazil / Amazonas / Manacapuru Human Original Oropharyngeal swab Illumina MiSeq BBMap 37.25 embedded in Geneious 10.2.175 B.1.1.28 GR
hCoV-19/Brazil/AM-20895258MP/2020 EPI_ISL_1068250 2020-06-22 South America / Brazil / Amazonas / Manaus Human Original Oropharyngeal swab Illumina MiSeq BBMap 37.25 embedded in Geneious 10.2.178 Gap of 3 nucleotides when compared to the reference sequence. info B.1.1.28 GR
hCoV-19/Brazil/AM-20895548CN/2020 EPI_ISL_1068251 2020-07-06 South America / Brazil / Amazonas / Manaus Human Original Oropharyngeal swab Illumina MiSeq BBMap 37.25 embedded in Geneious 10.2.179 B.1.1.29 GR
hCoV-19/Brazil/AM-20895554JT/2020 EPI_ISL_1068252 2020-09-16 South America / Brazil / Amazonas / Manaus Human Original Oropharyngeal swab Illumina MiSeq BBMap 37.25 embedded in Geneious 10.2.180 B.1.1.28 GR
hCoV-19/Brazil/AM-20895570KS/2020 EPI_ISL_1068253 2020-09-16 South America / Brazil / Amazonas / Manaus Human Original Oropharyngeal

swab	Illumina MiSeq	BBMap 37.25 embedded in Geneious 10.2.181
B.1.1.28	GR
hCoV-19/Brazil/AM-20895654ID/2020 EPI_ISL_1068254	2020-07-21	South America / Brazil / Amazonas / Manaus	Human	Original	Oropharyngeal swab	Illumina MiSeq	BBMap 37.25 embedded in Geneious 10.2.182
B.1.1.289	GR
hCoV-19/Brazil/BA-03/2020	EPI_ISL_1068317	2020-06-28	South America / Brazil / Bahia	Human	Original		Ion Torrent	Genome Detective
B.1.1.33	GR
hCoV-19/Brazil/BA-05/2020	EPI_ISL_1068319	2020-05-21	South America / Brazil / Bahia	Human	Original		Ion Torrent	Genome Detective
B.1.1.94	GR
hCoV-19/Brazil/BA-08/2020	EPI_ISL_1068322	2020-05-30	South America / Brazil / Bahia	Human	Original		Ion Torrent	Genome Detective
B.1.1.162	GR
hCoV-19/Brazil/BA-10.1/2020 EPI_ISL_1068324	2020-08-19	South America / Brazil / Bahia	Human	Original		Ion Torrent	Genome Detective
B.1.1.33	GR
hCoV-19/Brazil/BA-11.1/2020 EPI_ISL_1068325	2020-06-28	South America / Brazil / Bahia	Human	Original		Ion Torrent	Genome Detective
B.1	G
hCoV-19/Brazil/BA-12.1/2020 EPI_ISL_1068326	2020-07-30	South America / Brazil / Bahia	Human	Original		Ion Torrent	Genome Detective
B.1.1.33	GR
hCoV-19/Brazil/BA-13.1/2020 EPI_ISL_1068327	2020-07-23	South America / Brazil / Bahia	Human	Original		Ion Torrent	Genome Detective
B.1.1.28	GR
hCoV-19/Brazil/BA-14.1/2020 EPI_ISL_1068328	2020-08-03	South America / Brazil / Bahia	Human	Original		Ion Torrent	Genome Detective
B.1.1.33	GR
hCoV-19/Brazil/BA-15.1/2020 EPI_ISL_1068329	2020-08-18	South America / Brazil / Bahia	Human	Original		Ion Torrent	Genome Detective
N.4	GR
hCoV-19/Brazil/BA-80/2020	EPI_ISL_1068363	2020-05-05	South America / Brazil / Bahia	Human	Original		Ion Torrent	Genome Detective
B.1.1.28	GR
hCoV-19/Brazil/BA-81/2020	EPI_ISL_1068364	2020-05-17	South America / Brazil / Bahia	Human	Original		Ion Torrent	Genome Detective
B.1.1.28	GR
hCoV-19/Brazil/BA-83/2020	EPI_ISL_1068366	2020-05-04	South America / Brazil / Bahia	Human	Original		Ion Torrent	Genome Detective
B.1.1.33	GR
hCoV-19/Brazil/BA-84/2020	EPI_ISL_1068367	2020-05-27	South America / Brazil / Bahia	Human	Original		Ion Torrent	Genome Detective
B.1.1.33	GR
hCoV-19/Brazil/BA-86/2020	EPI_ISL_1068369	2020-05-19	South America / Brazil / Bahia	Human	Original		Ion Torrent	Genome Detective
B.1.1.28	GR
hCoV-19/Brazil/BA-87/2020	EPI_ISL_1068370	2020-05-07	South America / Brazil / Bahia	Human	Original		Ion Torrent	Genome Detective
B.1	G
hCoV-19/Brazil/BA-88/2020	EPI_ISL_1068371	2020-05-11	South America / Brazil / Bahia	Human	Original		Ion Torrent	Genome Detective
B.1.1.28	GR
hCoV-19/Brazil/BA-89/2020	EPI_ISL_1068372	2020-05-17	South America / Brazil / Bahia	Human	Original		Ion Torrent	Genome Detective
B.1.1.33	GR

hCoV-19/Brazil/BA-90/2020 EPI_ISL_1068373 2020-05-25 South America / Brazil / Bahia Human Original Ion Torrent Genome Detective B.1.1.143 GR
hCoV-19/Brazil/BA-91/2020 EPI_ISL_1068374 2020-05-09 South America / Brazil / Bahia Human Original Ion Torrent Genome Detective B.1.1.33 GR
hCoV-19/Brazil/BA-92/2020 EPI_ISL_1068375 2020-05-02 South America / Brazil / Bahia Human Original Ion Torrent Genome Detective B.1.1.33 GR
hCoV-19/Brazil/BA-93/2020 EPI_ISL_1068376 2020-05-27 South America / Brazil / Bahia Human Original Ion Torrent Genome Detective B.1.1.28 GR
hCoV-19/Brazil/BA-94/2020 EPI_ISL_1068377 2020-05-24 South America / Brazil / Bahia Human Original Ion Torrent Genome Detective B.1.1.28 GR
hCoV-19/Brazil/BA-95/2020 EPI_ISL_1068378 2020-05-03 South America / Brazil / Bahia Human Original Ion Torrent Genome Detective B.1.1.28 GR
hCoV-19/Brazil/PE-UFPE043/2020 EPI_ISL_1117379 2020-07-03 South America / Brazil / Pernambuco / Gravata Human Original Nasopharyngeal swab Illumina MiSeq Samtools, BWA NS8_Q18stop results in 86.0% truncation of the protein sequence. info B.1.1.29 GR
hCoV-19/Brazil/PE-UFPE044/2020 EPI_ISL_1117380 2020-07-03 South America / Brazil / Pernambuco / Gravata Human Original Nasopharyngeal swab Illumina MiSeq Samtools, BWA NS8_Q18stop results in 86.0% truncation of the protein sequence. info B.1.1.29 GR
hCoV-19/Brazil/PE-UFPE045/2020 EPI_ISL_1117381 2020-07-03 South America / Brazil / Pernambuco / Gravata Human Original Nasopharyngeal swab Illumina MiSeq Samtools, BWA NS8_Q18stop results in 86.0% truncation of the protein sequence. info B.1.1.29 GR
hCoV-19/Brazil/PE-UFPE046/2020 EPI_ISL_1117382 2020-07-03 South America / Brazil / Pernambuco / Gravata Human Original Nasopharyngeal swab Illumina MiSeq Samtools, BWA NS8_Q18stop results in 86.0% truncation of the protein sequence. info B.1.1.29 GR
hCoV-19/Brazil/PE-UFPE053/2020 EPI_ISL_1117383 2020-06-02 South America / Brazil / Pernambuco / Recife Human Original Nasopharyngeal swab Illumina MiSeq Samtools, BWA NS7a_Q62stop results in 49.6% truncation of the protein sequence. info B.1.1.29 GR
hCoV-19/Brazil/PE-UFPE032/2020 EPI_ISL_1117384 2020-07-02 South America / Brazil / Pernambuco / Araripina Human Original Nasopharyngeal swab Illumina MiSeq Samtools, BWA B.1.1.28 GR
hCoV-19/Brazil/PE-UFPE049/2020 EPI_ISL_1117385 2020-07-03 South America / Brazil / Pernambuco / Barra de Guabiraba Human Original Nasopharyngeal swab Illumina MiSeq Samtools, BWA B.1.1.74 GR
hCoV-19/Brazil/PE-UFPE033/2020 EPI_ISL_1117386 2020-07-02 South America / Brazil / Pernambuco / Bezerros Human Original Nasopharyngeal swab Illumina MiSeq Samtools, BWA B.1.1.29 GR
hCoV-19/Brazil/PE-UFPE001/2020 EPI_ISL_1117387 2020-06-24 South America / Brazil / Pernambuco / Caruaru Human Original Nasopharyngeal swab Illumina MiSeq Samtools, BWA B.1.1.29 GR
hCoV-19/Brazil/PE-UFPE002/2020 EPI_ISL_1117388 2020-06-24 South America / Brazil / Pernambuco / Caruaru Human Original Nasopharyngeal swab Illumina MiSeq Samtools, BWA B.1.1.28 GR

hCoV-19/Brazil/PE-UFPE003/2020 EPI_ISL_1117389 2020-06-24 South America / Brazil / Pernambuco / Caruaru Human Original Nasopharyngeal swab Illumina MiSeq Samtools, BWA B.1.1.29 GR
hCoV-19/Brazil/PE-UFPE004/2020 EPI_ISL_1117390 2020-06-24 South America / Brazil / Pernambuco / Caruaru Human Original Nasopharyngeal swab Illumina MiSeq Samtools, BWA B.1.1.33 GR
hCoV-19/Brazil/PE-UFPE010/2020 EPI_ISL_1117391 2020-06-25 South America / Brazil / Pernambuco / Caruaru Human Original Nasopharyngeal swab Illumina MiSeq Samtools, BWA B.1.1.192 GR
hCoV-19/Brazil/PE-UFPE011/2020 EPI_ISL_1117392 2020-06-25 South America / Brazil / Pernambuco / Caruaru Human Original Nasopharyngeal swab Illumina MiSeq Samtools, BWA B.1.1.29 GR
hCoV-19/Brazil/PE-UFPE016/2020 EPI_ISL_1117393 2020-06-26 South America / Brazil / Pernambuco / Caruaru Human Original Nasopharyngeal swab Illumina MiSeq Samtools, BWA B.1.1.314 GR
hCoV-19/Brazil/PE-UFPE017/2020 EPI_ISL_1117394 2020-06-26 South America / Brazil / Pernambuco / Caruaru Human Original Nasopharyngeal swab Illumina MiSeq Samtools, BWA B.1.1.29 GR
hCoV-19/Brazil/PE-UFPE018/2020 EPI_ISL_1117395 2020-06-26 South America / Brazil / Pernambuco / Caruaru Human Original Nasopharyngeal swab Illumina MiSeq Samtools, BWA B.1.1.33 GR
hCoV-19/Brazil/PE-UFPE019/2020 EPI_ISL_1117396 2020-06-26 South America / Brazil / Pernambuco / Caruaru Human Original Nasopharyngeal swab Illumina MiSeq Samtools, BWA B.1.1.29 GR
hCoV-19/Brazil/PE-UFPE012/2020 EPI_ISL_1117397 2020-06-26 South America / Brazil / Pernambuco / Cha Grande Human Original Nasopharyngeal swab Illumina MiSeq Samtools, BWA B.1.1.29 GR
hCoV-19/Brazil/PE-UFPE013/2020 EPI_ISL_1117398 2020-06-26 South America / Brazil / Pernambuco / Cha Grande Human Original Nasopharyngeal swab Illumina MiSeq Samtools, BWA B.1.1.29 GR
hCoV-19/Brazil/PE-UFPE014/2020 EPI_ISL_1117399 2020-06-26 South America / Brazil / Pernambuco / Cha Grande Human Original Nasopharyngeal swab Illumina MiSeq Samtools, BWA B.1.1.29 GR
hCoV-19/Brazil/PE-UFPE015/2020 EPI_ISL_1117400 2020-06-26 South America / Brazil / Pernambuco / Cha Grande Human Original Nasopharyngeal swab Illumina MiSeq Samtools, BWA B.1.1.29 GR
hCoV-19/Brazil/PE-UFPE041/2020 EPI_ISL_1117401 2020-07-03 South America / Brazil / Pernambuco / Gravata Human Original Nasopharyngeal swab Illumina MiSeq Samtools, BWA B.1.1.29 GR
hCoV-19/Brazil/PE-UFPE042/2020 EPI_ISL_1117402 2020-07-03 South America / Brazil / Pernambuco / Gravata Human Original Nasopharyngeal swab Illumina MiSeq Samtools, BWA B.1.1.33 GR
hCoV-19/Brazil/PE-UFPE047/2020 EPI_ISL_1117403 2020-07-03 South America / Brazil / Pernambuco / Gravata Human Original Nasopharyngeal swab Illumina MiSeq Samtools, BWA B.1.1.29 GR
hCoV-19/Brazil/PE-UFPE039/2020 EPI_ISL_1117404 2020-07-03 South America / Brazil / Pernambuco / Ibirajuba Human Original Nasopharyngeal swab Illumina MiSeq Samtools, BWA B.1.1.33 GR
hCoV-19/Brazil/PE-UFPE031/2020 EPI_ISL_1117405 2020-07-02 South America / Brazil / Pernambuco / Pesqueira Human Original Nasopharyngeal swab Illumina MiSeq Samtools, BWA B.1.1.33 GR

hCoV-19/Brazil/PE-UFPE040/2020 EPI_ISL_1117406 2020-07-03 South America / Brazil / Pernambuco / Pesqueira Human Original Nasopharyngeal swab Illumina MiSeq Samtools, BWA B.1.1.29 GR
hCoV-19/Brazil/PE-UFPE051/2020 EPI_ISL_1117407 2020-06-02 South America / Brazil / Pernambuco / Recife Human Original Nasopharyngeal swab Illumina MiSeq Samtools, BWA B.1.1.155 GR
hCoV-19/Brazil/PE-UFPE052/2020 EPI_ISL_1117408 2020-06-02 South America / Brazil / Pernambuco / Recife Human Original Nasopharyngeal swab Illumina MiSeq Samtools, BWA B.1.1.28 GR
hCoV-19/Brazil/PE-UFPE054/2020 EPI_ISL_1117409 2020-06-02 South America / Brazil / Pernambuco / Recife Human Original Nasopharyngeal swab Illumina MiSeq Samtools, BWA B.1.1.29 GR
hCoV-19/Brazil/PE-UFPE055/2020 EPI_ISL_1117410 2020-06-02 South America / Brazil / Pernambuco / Recife Human Original Nasopharyngeal swab Illumina MiSeq Samtools, BWA B.1.1.67 GR
hCoV-19/Brazil/PE-UFPE056/2020 EPI_ISL_1117411 2020-06-02 South America / Brazil / Pernambuco / Recife Human Original Nasopharyngeal swab Illumina MiSeq Samtools, BWA B.1.1.29 GR
hCoV-19/Brazil/PE-UFPE057/2020 EPI_ISL_1117412 2020-06-03 South America / Brazil / Pernambuco / Recife Human Original Nasopharyngeal swab Illumina MiSeq Samtools, BWA B.1.1.29 GR
hCoV-19/Brazil/PE-UFPE058/2020 EPI_ISL_1117413 2020-06-03 South America / Brazil / Pernambuco / Recife Human Original Nasopharyngeal swab Illumina MiSeq Samtools, BWA B.1.1.29 GR
hCoV-19/Brazil/PE-UFPE059/2020 EPI_ISL_1117414 2020-06-03 South America / Brazil / Pernambuco / Recife Human Original Nasopharyngeal swab Illumina MiSeq Samtools, BWA B.1.1.29 GR
hCoV-19/Brazil/PE-UFPE060/2020 EPI_ISL_1117415 2020-06-03 South America / Brazil / Pernambuco / Recife Human Original Nasopharyngeal swab Illumina MiSeq Samtools, BWA B.1.1.29 GR
hCoV-19/Brazil/PE-UFPE061/2020 EPI_ISL_1117416 2020-06-03 South America / Brazil / Pernambuco / Recife Human Original Nasopharyngeal swab Illumina MiSeq Samtools, BWA B.1.1.29 GR
hCoV-19/Brazil/PE-UFPE062/2020 EPI_ISL_1117417 2020-06-03 South America / Brazil / Pernambuco / Recife Human Original Nasopharyngeal swab Illumina MiSeq Samtools, BWA B.1.1.33 GR
hCoV-19/Brazil/PE-UFPE063/2020 EPI_ISL_1117418 2020-06-03 South America / Brazil / Pernambuco / Recife Human Original Nasopharyngeal swab Illumina MiSeq Samtools, BWA B.1.1.29 GR
hCoV-19/Brazil/PE-UFPE064/2020 EPI_ISL_1117419 2020-06-03 South America / Brazil / Pernambuco / Recife Human Original Nasopharyngeal swab Illumina MiSeq Samtools, BWA B.1.1.29 GR
hCoV-19/Brazil/PE-UFPE065/2020 EPI_ISL_1117420 2020-06-03 South America / Brazil / Pernambuco / Recife Human Original Nasopharyngeal swab Illumina MiSeq Samtools, BWA B.1.1.147 GR
hCoV-19/Brazil/PE-UFPE066/2020 EPI_ISL_1117421 2020-06-03 South America / Brazil / Pernambuco / Recife Human Original Nasopharyngeal swab Illumina MiSeq Samtools, BWA B.1.1.214 GR
hCoV-19/Brazil/PE-UFPE067/2020 EPI_ISL_1117422 2020-06-03 South America / Brazil / Pernambuco / Recife Human Original Nasopharyngeal swab Illumina MiSeq Samtools, BWA B.1.1.29 GR
hCoV-19/Brazil/PE-UFPE068/2020 EPI_ISL_1117423 2020-06-03 South America / Brazil / Pernambuco / Recife Human Original Nasopharyngeal swab Illumina MiSeq Samtools, BWA B.1.1.29 GR
hCoV-19/Brazil/PE-UFPE069/2020 EPI_ISL_1117424 2020-06-03 South America / Brazil / Pernambuco / Recife Human Original Nasopharyngeal swab Illumina MiSeq Samtools, BWA B.1.1.29 GR

hCoV-19/Brazil/PE-UFPE070/2020 EPI_ISL_1117425 2020-06-03 South America / Brazil / Pernambuco / Recife Human Original Nasopharyngeal swab Illumina MiSeq Samtools, BWA B.1.1.29 GR
hCoV-19/Brazil/PE-UFPE071/2020 EPI_ISL_1117426 2020-06-03 South America / Brazil / Pernambuco / Recife Human Original Nasopharyngeal swab Illumina MiSeq Samtools, BWA B.1.1.29 GR
hCoV-19/Brazil/PE-UFPE029/2020 EPI_ISL_1117427 2020-07-02 South America / Brazil / Pernambuco / Santa Cruz do Capibaribe Human Original Nasopharyngeal swab Illumina MiSeq Samtools, BWA B.1.1.29 GR
hCoV-19/Brazil/PE-UFPE030/2020 EPI_ISL_1117428 2020-07-02 South America / Brazil / Pernambuco / Santa Cruz do Capibaribe Human Original Nasopharyngeal swab Illumina MiSeq Samtools, BWA B.1.1.29 GR
hCoV-19/Brazil/PE-UFPE050/2020 EPI_ISL_1117429 2020-07-03 South America / Brazil / Pernambuco / Sao Bento do Una Human Original Nasopharyngeal swab Illumina MiSeq Samtools, BWA B.1.1.28 GR
hCoV-19/Brazil/PE-UFPE008/2020 EPI_ISL_1117430 2020-06-25 South America / Brazil / Pernambuco / Sao Caetano Human Original Nasopharyngeal swab Illumina MiSeq Samtools, BWA B.1.1.29 GR
hCoV-19/Brazil/PE-UFPE009/2020 EPI_ISL_1117431 2020-06-25 South America / Brazil / Pernambuco / Sao Caetano Human Original Nasopharyngeal swab Illumina MiSeq Samtools, BWA B.1.1.29 GR
hCoV-19/Brazil/PE-UFPE034/2020 EPI_ISL_1117432 2020-07-02 South America / Brazil / Pernambuco / Sao Caetano Human Original Nasopharyngeal swab Illumina MiSeq Samtools, BWA B.1.1.29 GR
hCoV-19/Brazil/PE-UFPE035/2020 EPI_ISL_1117433 2020-07-02 South America / Brazil / Pernambuco / Sao Caetano Human Original Nasopharyngeal swab Illumina MiSeq Samtools, BWA B.1.1.29 GR
hCoV-19/Brazil/PE-UFPE036/2020 EPI_ISL_1117434 2020-07-02 South America / Brazil / Pernambuco / Sao Caetano Human Original Nasopharyngeal swab Illumina MiSeq Samtools, BWA B.1.1.29 GR
hCoV-19/Brazil/PE-UFPE028/2020 EPI_ISL_1117435 2020-07-02 South America / Brazil / Pernambuco / Sao Joaquim do Monte Human Original Nasopharyngeal swab Illumina MiSeq Samtools, BWA B.1.1.29 GR
hCoV-19/Brazil/PE-UFPE037/2020 EPI_ISL_1117436 2020-07-03 South America / Brazil / Pernambuco / Sao Jose do Egito Human Original Nasopharyngeal swab Illumina MiSeq Samtools, BWA B.1.1.29 GR
hCoV-19/Brazil/PE-UFPE020/2020 EPI_ISL_1117437 2020-06-26 South America / Brazil / Pernambuco / Sao Lourenco da Mata Human Original Nasopharyngeal swab Illumina MiSeq Samtools, BWA B.1.1.29 GR
hCoV-19/Brazil/PE-UFPE021/2020 EPI_ISL_1117438 2020-06-26 South America / Brazil / Pernambuco / Sao Lourenco da Mata Human Original Nasopharyngeal swab Illumina MiSeq Samtools, BWA B.1.1.29 GR
hCoV-19/Brazil/PE-UFPE005/2020 EPI_ISL_1117439 2020-06-25 South America / Brazil / Pernambuco / Serra Talhada Human Original Nasopharyngeal swab Illumina MiSeq Samtools, BWA B.1.1.127 GR

hCoV-19/Brazil/PE-UFPE006/2020 EPI_ISL_1117440 2020-06-25 South America / Brazil / Pernambuco / Serra Talhada Human Original Nasopharyngeal swab Illumina MiSeq Samtools, BWA B.1.1.33 GR
hCoV-19/Brazil/PE-UFPE007/2020 EPI_ISL_1117441 2020-06-25 South America / Brazil / Pernambuco / Serra Talhada Human Original Nasopharyngeal swab Illumina MiSeq Samtools, BWA B.1.1.29 GR
hCoV-19/Brazil/PE-UFPE038/2020 EPI_ISL_1117442 2020-07-03 South America / Brazil / Pernambuco / Serra Talhada Human Original Nasopharyngeal swab Illumina MiSeq Samtools, BWA B.1.1.33 GR
hCoV-19/Brazil/PE-UFPE022/2020 EPI_ISL_1117443 2020-06-29 South America / Brazil / Pernambuco / Toritama Human Original Nasopharyngeal swab Illumina MiSeq Samtools, BWA B.1.212 G
hCoV-19/Brazil/PE-UFPE023/2020 EPI_ISL_1117444 2020-06-29 South America / Brazil / Pernambuco / Toritama Human Original Nasopharyngeal swab Illumina MiSeq Samtools, BWA B.1.1.29 GR
hCoV-19/Brazil/PE-UFPE024/2020 EPI_ISL_1117445 2020-06-29 South America / Brazil / Pernambuco / Toritama Human Original Nasopharyngeal swab Illumina MiSeq Samtools, BWA B.1.1.29 GR
hCoV-19/Brazil/PE-UFPE025/2020 EPI_ISL_1117446 2020-07-02 South America / Brazil / Pernambuco / Toritama Human Original Nasopharyngeal swab Illumina MiSeq Samtools, BWA B.1.1.29 GR
hCoV-19/Brazil/PE-UFPE026/2020 EPI_ISL_1117447 2020-07-02 South America / Brazil / Pernambuco / Toritama Human Original Nasopharyngeal swab Illumina MiSeq Samtools, BWA B.1.1.29 GR
hCoV-19/Brazil/PE-UFPE027/2020 EPI_ISL_1117448 2020-07-02 South America / Brazil / Pernambuco / Toritama Human Original Nasopharyngeal swab Illumina MiSeq Samtools, BWA B.1.1.29 GR
hCoV-19/Brazil/PE-UFPE048/2020 EPI_ISL_1117449 2020-07-03 South America / Brazil / Pernambuco / Toritama Human Original Nasopharyngeal swab Illumina MiSeq Samtools, BWA B.1.1.29 GR
hCoV-19/Brazil/SP-1456R1/2020 EPI_ISL_1121326 2020-08-17 South America / Brazil / Sao Paulo / Uru Human Original Nasopharyngeal swab Ion Torrent S5 IRMA B.1.1.28 GR
hCoV-19/Brazil/MS-1270/2020 EPI_ISL_1121327 2020-07-31 South America / Brazil / Mato Grosso do Sul / Campo Grande Human Original Nasopharyngeal swab Ion Torrent S5 IRMA B.1.1.33 GR
hCoV-19/Brazil/MS-1280/2020 EPI_ISL_1121328 2020-07-22 South America / Brazil / Mato Grosso do Sul / Sidrolandia Human Original Nasopharyngeal swab Ion Torrent S5 IRMA B.1.1.33 GR
hCoV-19/Brazil/MS-1285/2020 EPI_ISL_1121329 2020-07-03 South America / Brazil / Mato Grosso do Sul / Campo Grande Human Original Nasopharyngeal swab Ion Torrent S5 IRMA B.1.1.28 GR
hCoV-19/Brazil/MS-1286/2020 EPI_ISL_1121330 2020-07-03 South America / Brazil / Mato Grosso do Sul / Douradinho Human Original

Nasopharyngeal swab Ion Torrent S5 IRMA
B.1.1.33 GR
hCoV-19/Brazil/MS-1294/2020 EPI_ISL_1139052 2020-06-27 South America / Brazil / Mato Grosso do Sul / Itaquirai Human Original Nasopharyngeal swab Ion Torrent S5 IRMA B.1.1.28 GR
hCoV-19/Brazil/MS-1295/2020 EPI_ISL_1139053 2020-06-27 South America / Brazil / Mato Grosso do Sul / Dourados Human Original Nasopharyngeal swab Ion Torrent S5 IRMA B.1.1.33 GR
hCoV-19/Brazil/MS-1298/2020 EPI_ISL_1139054 2020-05-17 South America / Brazil / Mato Grosso do Sul / Campo Grande Human Original
Nasopharyngeal swab Ion Torrent S5 IRMA
B.1.1.28 GR
hCoV-19/Brazil/MS-1299/2020 EPI_ISL_1139055 2020-05-17 South America / Brazil / Mato Grosso do Sul / Vicentina Human Original Nasopharyngeal swab Ion Torrent S5 IRMA B.1.1.33 GR
hCoV-19/Brazil/MS-1303/2020 EPI_ISL_1139056 2020-05-14 South America / Brazil / Mato Grosso do Sul / Guia Lopes da Laguna Human Original
Nasopharyngeal swab Ion Torrent S5 IRMA
B.1.1.28 GR
hCoV-19/Brazil/MS-1304/2020 EPI_ISL_1139057 2020-05-14 South America / Brazil / Mato Grosso do Sul / Bonito Human Original Nasopharyngeal swab Ion Torrent S5 IRMA B.1.1.28 GR
hCoV-19/Brazil/RS-19RMNNHCPA/2020 EPI_ISL_1163701 2020-06 South America / Brazil / Rio Grande do Sul Human Original
Illumina MiSeq CLC Genomics Workbench 12 NS7a_Q62stop results in 49.6% truncation of the protein sequence. info B.1.1.161 O
hCoV-19/Brazil/RS-20IFHCPA/2020 EPI_ISL_1163702 2020-06 South America / Brazil / Rio Grande do Sul Human Original
Illumina MiSeq CLC Genomics Workbench 12 B.1.1.161
O
hCoV-19/Brazil/RS-22MSFHCPA/2020 EPI_ISL_1163703 2020-07 South America / Brazil / Rio Grande do Sul Human Original
Illumina MiSeq CLC Genomics Workbench 12 B.1.1.161
O
hCoV-19/Brazil/RS-23MBSHCPA/2020 EPI_ISL_1163704 2020-07 South America / Brazil / Rio Grande do Sul Human Original
Illumina MiSeq CLC Genomics Workbench 12 B.1.1.28
O
hCoV-19/Brazil/RS-24RPSHCPA/2020 EPI_ISL_1163705 2020-07 South America / Brazil / Rio Grande do Sul Human Original
Illumina MiSeq CLC Genomics Workbench 12 B.1.1.33
GR
hCoV-19/Brazil/RS-25RCFHCPA/2020 EPI_ISL_1163706 2020-08 South America / Brazil / Rio Grande do Sul Human Original
Illumina MiSeq CLC Genomics Workbench 12 B.1.1.161
O
hCoV-19/Brazil/RS-26AVGFHCPA/2020 EPI_ISL_1163707 2020-08 South America / Brazil / Rio Grande do Sul Human Original
Illumina MiSeq CLC Genomics Workbench 12 NS7a_Q62stop results in 49.6% truncation of the protein sequence. info B.1.1.161 O
hCoV-19/Brazil/RS-27IMCHCPA/2020 EPI_ISL_1163708 2020-08 South America / Brazil / Rio Grande do Sul Human Original
Illumina MiSeq CLC Genomics Workbench 12 B.1 O
hCoV-19/Brazil/RS-28DBCHCPA/2020 EPI_ISL_1163709 2020-09 South America / Brazil / Rio Grande do Sul Human Original
Illumina MiSeq CLC Genomics Workbench 12 B.1.1.28
O

hCoV-19/Brazil/RS-29VDCHCPA/2020 EPI_ISL_1163710 2020-09 South America / Brazil / Rio Grande do Sul Human Original Illumina MiSeq CLC Genomics Workbench 12 B.1.1.28 O
hCoV-19/Brazil/RS-30HGGHCPA/2020 EPI_ISL_1163711 2020-09 South America / Brazil / Rio Grande do Sul Human Original Illumina MiSeq CLC Genomics Workbench 12 B.1.1.28 O
hCoV-19/Brazil/SP-1104/2020 EPI_ISL_1171620 2020-08-14 South America / Brazil / Sao Paulo / Sao Paulo Human Original Nasopharyngeal swab Ion Torrent S5 IRMA B.1.1.28 GR
hCoV-19/Brazil/MG-FUNED-09-210/2020 EPI_ISL_1182550 2020-05-21 South America / Brazil / Minas Gerais / Sabara Human Original Re-infection sample first sample "FUNED_09.21-RI-MG" second sample "FUNED_10.21-RI-MG" Ion Torrent B.1.1.28 GR
hCoV-19/Brazil/RJ-UFRJ-2517/2020 EPI_ISL_492035 2020-05-29 South America / Brazil / Rio de Janeiro / Rio de Janeiro Human Original Oropharyngeal swab Ion Torrent S5 IRMA v1.2.1.0 B.1.1.33 GR
hCoV-19/Brazil/RJ-UFRJ-9331/2020 EPI_ISL_492036 2020-06-01 South America / Brazil / Rio de Janeiro / Resende Human Original Oropharyngeal swab Ion Torrent S5 IRMA v1.2.1.0 B.1.1.28 GR
hCoV-19/Brazil/RJ-UFRJ-57437/2020 EPI_ISL_492043 2020-05-25 South America / Brazil / Rio de Janeiro / Rio de Janeiro Human Original Oropharyngeal swab Ion Torrent S5 IRMA v1.2.1.0 B.1.1.33 GR
hCoV-19/Brazil/RJ-UFRJ-57721/2020 EPI_ISL_492044 2020-05-27 South America / Brazil / Rio de Janeiro / Rio de Janeiro Human Original Oropharyngeal swab Ion Torrent S5 IRMA v1.2.1.0 B.1.1.33 GR
hCoV-19/Brazil/RJ-UFRJ-57722/2020 EPI_ISL_492045 2020-05-27 South America / Brazil / Rio de Janeiro / Rio de Janeiro Human Original Oropharyngeal swab Ion Torrent S5 IRMA v1.2.1.0 B.1.1.33 GR
hCoV-19/Brazil/RJ-UFRJ-58090/2020 EPI_ISL_492046 2020-05-29 South America / Brazil / São Paulo / São Paulo Human Original Oropharyngeal swab Ion Torrent S5 IRMA v1.2.1.0 B.1.1.33 GR
hCoV-19/Brazil/RJ-UFRJ-58110/2020 EPI_ISL_492047 2020-05-29 South America / Brazil / São Paulo / São Paulo Human Original Oropharyngeal swab Ion Torrent S5 IRMA v1.2.1.0 B.1.1.33 GR
hCoV-19/Brazil/RJ-UFRJ-58271/2020 EPI_ISL_492048 2020-06-01 South America / Brazil / Rio de Janeiro / Rio de Janeiro Human Original Oropharyngeal swab Ion Torrent S5 IRMA v1.2.1.0 B.1.1.33 GR
hCoV-19/Brazil/PE-AMU0036/2020 EPI_ISL_502779 2020-06-24 South America / Brazil / Pernambuco / Caruaru Human Original Nasopharyngeal swab Illumina MiSeq BWA, Samtools B.1 G
hCoV-19/Brazil/PE-COV0260/2020 EPI_ISL_502875 2020-06-24 South America / Brazil / Pernambuco / Recife Human Original Nasopharyngeal swab Illumina MiSeq BWA, Samtools B.1.1.28 G
hCoV-19/Brazil/RJ-DCV05/2020EPI_ISL_509430 2020-07-20 South America / Brazil / Rio de Janeiro Human Vero E6 MGI SPAdes v. JULY-2020 B.1 G

hCoV-19/Brazil/RJ-DCV1/2020 EPI_ISL_509431 2020-07-20 South America / Brazil / Rio de Janeiro Human Vero E6 MGI SPAdes v. JULY-2020 B.1 G
hCoV-19/Brazil/RJ-DCV3/2020 EPI_ISL_509432 2020-07-20 South America / Brazil / Rio de Janeiro Human Vero E6 MGI SPAdes v. JULY-2020 B.1 G
hCoV-19/Brazil/RJ-DCV5/2020 EPI_ISL_509433 2020-07-20 South America / Brazil / Rio de Janeiro Human Vero E6 MGI SPAdes v. JULY-2020 B.1 G
hCoV-19/Brazil/RJ-DCVTV507Q/2020 EPI_ISL_509435 2020-07-20 South America / Brazil / Rio de Janeiro Human Vero E6 MGI SPAdes v. JULY-2020 B.1 G
hCoV-19/Brazil/PR-LRV-01/2020 EPI_ISL_510535 2020-05-08 South America / Brazil / Paraná / Curitiba Human Original Nasopharyngeal swab Illumina CLC Genomics Workbench v. 20.0.4 B.1.1.33 GR
hCoV-19/Brazil/RJ-DCVN5/2020EPI_ISL_510536 2020-07-20 South America / Brazil / Rio de Janeiro Human Vero E6 MGI SPAdes v. JULY-2020 B.1 G
hCoV-19/Brazil/RJ-DCVN4/2020EPI_ISL_510541 2020-07-20 South America / Brazil / Rio de Janeiro Human Vero E6 MGI SPAdes v. JULY-2020 B.1 G
hCoV-19/Brazil/RJ-INCA-I29/2020 EPI_ISL_513573 2020-05-04 South America / Brazil / Rio de Janeiro Human Original Nasopharyngeal and oropharyngeal swab Illumina Miseq / Sanger Geneious 11.1.5 B.1.1.33 GR
hCoV-19/Brazil/RJ-INCA-I30/2020 EPI_ISL_513574 2020-05-04 South America / Brazil / Rio de Janeiro Human Original Nasopharyngeal and oropharyngeal swab Illumina Miseq / Sanger Geneious 11.1.5 B.1.1.33 GR
hCoV-19/Brazil/RJ-INCA-I34/2020 EPI_ISL_513575 2020-05-04 South America / Brazil / Rio de Janeiro Human Original Nasopharyngeal and oropharyngeal swab Illumina Miseq / Sanger Geneious 11.1.5 B.1.1.33 GR
hCoV-19/Brazil/RJ-INCA-I35/2020 EPI_ISL_513576 2020-05-04 South America / Brazil / Rio de Janeiro Human Original Nasopharyngeal and oropharyngeal swab Illumina Miseq / Sanger Geneious 11.1.5 B.1.1.33 GR
hCoV-19/Brazil/RJ-INCA-I37/2020 EPI_ISL_513577 2020-05-04 South America / Brazil / Rio de Janeiro Human Original Nasopharyngeal and oropharyngeal swab Illumina Miseq / Sanger Geneious 11.1.5 B.1.1.33 GR
hCoV-19/Brazil/RJ-INCA-I39/2020 EPI_ISL_513578 2020-05-04 South America / Brazil / Rio de Janeiro Human Original Nasopharyngeal and oropharyngeal swab Illumina Miseq / Sanger Geneious 11.1.5 B.1.1.28 GR
hCoV-19/Brazil/RJ-INCA-I43/2020 EPI_ISL_513579 2020-05-04 South America / Brazil / Rio de Janeiro Human Original Nasopharyngeal and oropharyngeal swab Illumina Miseq / Sanger Geneious 11.1.5 B.1.1.33 GR
hCoV-19/Brazil/RJ-INCA-I44/2020 EPI_ISL_513580 2020-05-04 South America / Brazil / Rio de Janeiro Human Original Nasopharyngeal and oropharyngeal swab Illumina Miseq / Sanger Geneious 11.1.5 B.1.1.33 GR
hCoV-19/Brazil/RJ-INCA-I51/2020 EPI_ISL_513581 2020-05-05 South America / Brazil / Rio de Janeiro Human Original Nasopharyngeal and oropharyngeal swab Illumina Miseq / Sanger Geneious 11.1.5 B.1.1.33 GR

hCoV-19/Brazil/RJ-INCA-I54/2020 EPI_ISL_513582 2020-05-05 South America / Brazil / Rio de Janeiro Human Original Nasopharyngeal and oropharyngeal swab Illumina Miseq / Sanger Geneious 11.1.5 B.1.1.33 GR
hCoV-19/Brazil/RJ-INCA-I57/2020 EPI_ISL_513583 2020-05-05 South America / Brazil / Rio de Janeiro Human Original Nasopharyngeal and oropharyngeal swab Illumina Miseq / Sanger Geneious 11.1.5 B.1.1.33 GR
hCoV-19/Brazil/RO-01/2020 EPI_ISL_514131 2020-05-11 South America / Brazil / Rondônia / Jaru Human Original Illumina MiSeq B.1.1.33 GR
hCoV-19/Brazil/RO-02/2020 EPI_ISL_514132 2020-05-11 South America / Brazil / Rondônia / Porto Velho Human Original Illumina MiSeq B.1.1.33 GR
hCoV-19/Brazil/RO-03/2020 EPI_ISL_514133 2020-05-11 South America / Brazil / Rondônia / Porto Velho Human Original Illumina MiSeq B.1.1.33 GR
hCoV-19/Brazil/RO-04/2020 EPI_ISL_514134 2020-05-11 South America / Brazil / Rondônia / Porto Velho Human Original Illumina MiSeq B.1.212 G
hCoV-19/Brazil/RO-05/2020 EPI_ISL_514135 2020-05-05 South America / Brazil / Rondônia / Porto Velho Human Original Illumina MiSeq B.1.1.33 GR
hCoV-19/Brazil/RO-06/2020 EPI_ISL_514136 2020-05-05 South America / Brazil / Rondônia / Porto Velho Human Original Illumina MiSeq B.1.212 G
hCoV-19/Brazil/RO-07/2020 EPI_ISL_514137 2020-05-05 South America / Brazil / Rondônia / Porto Velho Human Original Illumina MiSeq B.1.1.33 GR
hCoV-19/Brazil/RO-08/2020 EPI_ISL_514138 2020-06-05 South America / Brazil / Rondônia / Porto Velho Human Original Illumina MiSeq B.1.1.33 GR
hCoV-19/Brazil/SP-600/2020 EPI_ISL_515525 2020-06-30 South America / Brazil / São Paulo / São Paulo Human Original Nasopharyngeal swab Ion Torrent S5 IRMA B.1.1.33 GR
hCoV-19/Brazil/SP-163/2020 EPI_ISL_523959 2020-08-15 South America / Brazil / São Paulo / São Paulo Human Original Nasopharyngeal swab Ion Torrent S5 IRMA B.1.1.33 GR
hCoV-19/Brazil/SP-240/2020 EPI_ISL_524468 2020-05-01 South America / Brazil / São Paulo / São Paulo Human Original Nasopharyngeal swab Ion Torrent S5 IRMA B.1.1.28 GR
hCoV-19/Brazil/SP-254/2020 EPI_ISL_524469 2020-05-05 South America / Brazil / São Paulo / São Paulo Human Original Nasopharyngeal swab Ion Torrent S5 IRMA B.1.1.28 GR
hCoV-19/Brazil/SP-259/2020 EPI_ISL_524470 2020-05-06 South America / Brazil / São Paulo / São Paulo Human Original Nasopharyngeal swab Ion Torrent S5 IRMA B.1.1.33 GR
hCoV-19/Brazil/RJ-DCVN2/2020EPI_ISL_529139 2020-07-20 South America / Brazil / Rio de Janeiro Human Vero E6 MGI SPAdes v. JULY-2020 B.1 G
hCoV-19/Brazil/RJ-DCVN3/2020EPI_ISL_529140 2020-07-20 South America / Brazil / Rio de Janeiro Human Vero E6 MGI SPAdes v. JULY-2020 B.1 G
hCoV-19/Brazil/SP-315/2020 EPI_ISL_534312 2020-05-01 South America / Brazil / São Paulo / Campinas Human Original Nasopharyngeal swab Ion Torrent S5 IRMA B.1.1.33 GR

hCoV-19/Brazil/SP-317/2020 EPI_ISL_534314 2020-05-04 South America / Brazil / São Paulo / São Paulo Human Original Nasopharyngeal swab Ion Torrent S5 IRMA B.1.1.28 GR
hCoV-19/Brazil/SP-318/2020 EPI_ISL_534315 2020-05-02 South America / Brazil / São Paulo / Guarulhos Human Original Nasopharyngeal swab Ion Torrent S5 IRMA B.1.1.33 GR
hCoV-19/Brazil/SP-321/2020 EPI_ISL_534316 2020-05-02 South America / Brazil / São Paulo Human Original Nasopharyngeal swab Ion Torrent S5 IRMA B.1.1.28 GR
hCoV-19/Brazil/SP-324/2020 EPI_ISL_534317 2020-05-05 South America / Brazil / São Paulo / JandiraHuman Original Nasopharyngeal swab Ion Torrent S5 IRMA B.1.1.28 GR
hCoV-19/Brazil/SP-325/2020 EPI_ISL_534318 2020-05-03 South America / Brazil / São Paulo / Osasco Human Original Nasopharyngeal swab Ion Torrent S5 IRMA B.1.1.28 GR
hCoV-19/Brazil/SP-255/2020 EPI_ISL_534319 2020-05-06 South America / Brazil / São Paulo / São Paulo Human Original Nasopharyngeal swab Ion Torrent S5 IRMA B.1.1.28 GR
hCoV-19/Brazil/SP-256/2020 EPI_ISL_534320 2020-05-05 South America / Brazil / São Paulo / São Paulo Human Original Nasopharyngeal swab Ion Torrent S5 IRMA B.1.1.28 GR
hCoV-19/Brazil/SP-329/2020 EPI_ISL_534321 2020-05-07 South America / Brazil / São Paulo / BarueriHuman Original Nasopharyngeal swab Ion Torrent S5 IRMA B.1.1.28 GR
hCoV-19/Brazil/SP-340/2020 EPI_ISL_534326 2020-05-01 South America / Brazil / São Paulo / Cotia Human Original Nasopharyngeal swab Ion Torrent S5 IRMA B.1.1.28 GR
hCoV-19/Brazil/SP-341/2020 EPI_ISL_547571 2020-06-01 South America / Brazil / São Paulo / Maua Human Original Nasopharyngeal swab Ion Torrent S5 IRMA B.1.1.314 GR
hCoV-19/Brazil/SP-342/2020 EPI_ISL_547573 2020-06-01 South America / Brazil / São Paulo / CajamarHuman Original Nasopharyngeal swab Ion Torrent S5 IRMA B.1.1.28 GR
hCoV-19/Brazil/SP-354/2020 EPI_ISL_547574 2020-06-19 South America / Brazil / São Paulo / Taboao da Serra Human Original Nasopharyngeal swab Ion Torrent S5 IRMA B.1.1.33 GR
hCoV-19/Brazil/SP-356/2020 EPI_ISL_547575 2020-06-16 South America / Brazil / São Paulo / JundiaiHuman Original Nasopharyngeal swab Ion Torrent S5 IRMA B.1.1.28 GR
hCoV-19/Brazil/SP-358/2020 EPI_ISL_547576 2020-06-23 South America / Brazil / São Paulo / BiriguiHuman Original Nasopharyngeal swab Ion Torrent S5 IRMA B.1.1.143 GR
hCoV-19/Brazil/SP-405/2020 EPI_ISL_547577 2020-07-06 South America / Brazil / São Paulo / Presidente Prudente Human Original Nasopharyngeal swab ion Torrent S5 IRMA N.4 GR
hCoV-19/Brazil/SP-406/2020 EPI_ISL_547578 2020-07-05 South America / Brazil / São Paulo / Presidente Prudente Human Original Nasopharyngeal swab Ion Torrent S5 IRMA B.1.1.33 GR
hCoV-19/Brazil/SP-417/2020 EPI_ISL_547579 2020-07-07 South America / Brazil / São Paulo / Araçatuba Human Original Nasopharyngeal swab Ion Torrent S5 IRMA B.1.1.28 GR
hCoV-19/Brazil/SP-418/2020 EPI_ISL_547580 2020-07-09 South America / Brazil / São Paulo / Euclides da Cunha Paulista Human Original Nasopharyngeal swab Ion Torrent S5 IRMA B.1.1.33 GR

hCoV-19/Brazil/PE-IAM961/2020 EPI_ISL_572366 2020-05-02 South America / Brazil / Pernambuco / Carpina Human Original unknown Illumina MiSeq Bowtie2 2.2.6/bedtools v2.25.0 B.1.1.29 GR
hCoV-19/Brazil/PE-IAM991/2020 EPI_ISL_572371 2020-05-06 South America / Brazil / Pernambuco / Recife Human Original unknown Illumina MiSeq Bowtie2 2.2.6/bedtools v2.25.0 B.1.1.28 GR
hCoV-19/Brazil/PE-IAM1309/2020 EPI_ISL_572386 2020-05-10 South America / Brazil / Pernambuco / Ipojuca Human Original unknown Illumina MiSeq Bowtie2 2.2.6/bedtools v2.25.0 B.1.1.186 GR
hCoV-19/Brazil/SP-425/2020 EPI_ISL_574594 2020-07-10 South America / Brazil / São Paulo / Taubate Human Original Nasopharyngeal swab Ion Torrent S5 IRMA B.1.1.94 GR
hCoV-19/Brazil/SP-388/2020 EPI_ISL_574596 2020-06-19 South America / Brazil / São Paulo / Sumare Human Original Nasopharyngeal swab Ion Torrent S5 IRMA B.1.1.33 GR
hCoV-19/Brazil/SP-389/2020 EPI_ISL_574597 2020-06-18 South America / Brazil / São Paulo / Jarinu Human Original Nasopharyngeal swab Ion Torrent S5 IRMA B.1.1.28 GR
hCoV-19/Brazil/SP-391/2020 EPI_ISL_574598 2020-06-20 South America / Brazil / São Paulo / Jundiai Human Original Nasopharyngeal swab Ion Torrent S5 IRMA B.1.1.28 GR
hCoV-19/Brazil/SP-345/2020 EPI_ISL_583495 2020-06-13 South America / Brazil / São Paulo / Guarulhos Human Original Nasopharyngeal swab Ion Torrent S5 IRMA B.1.1.33 GR
hCoV-19/Brazil/SP-353/2020 EPI_ISL_583496 2020-06-20 South America / Brazil / São Paulo / Jandira Human Original Nasopharyngeal swab Ion Torrent S5 IRMA B.1.1.28 GR
hCoV-19/Brazil/SP-359/2020 EPI_ISL_583497 2020-06-17 South America / Brazil / São Paulo / Campinas Human Original Nasopharyngeal swab Ion Torrent S5 IRMA B.1.1.28 GR
hCoV-19/Brazil/SP-360/2020 EPI_ISL_583498 2020-06-22 South America / Brazil / São Paulo / Americana Human Original Nasopharyngeal swab Ion Torrent S5 IRMA B.1.1.28 GR
hCoV-19/Brazil/SP-368/2020 EPI_ISL_583499 2020-06-24 South America / Brazil / São Paulo / Campinas Human Original Nasopharyngeal swab Ion Torrent S5 IRMA B.1.1.143 GR
hCoV-19/Brazil/SP-370/2020 EPI_ISL_583500 2020-06-29 South America / Brazil / São Paulo / Presidente Venceslau Human Original Nasopharyngeal swab Ion Torrent S5 IRMA B.1.1.28 GR
hCoV-19/Brazil/SP-407/2020 EPI_ISL_583501 2020-07-08 South America / Brazil / São Paulo / São Paulo Human Original Nasopharyngeal swab Ion Torrent S5 IRMA B.1.1.143 GR
hCoV-19/Brazil/SP-393/2020 EPI_ISL_583502 2020-06-21 South America / Brazil / São Paulo / Guaruja Human Original Ion Torrent S5 IRMA B.1.1.28 GR
hCoV-19/Brazil/SP-394/2020 EPI_ISL_583503 2020-06-22 South America / Brazil / São Paulo / Caieiras Human Original Ion Torrent S5 IRMA B.1.1.28 GR
hCoV-19/Brazil/SP-395/2020 EPI_ISL_583504 2020-06-20 South America / Brazil / São Paulo / Caraguatatuba Human Original Nasopharyngeal swab Ion Torrent S5 IRMA B.1.1.28 GR
hCoV-19/Brazil/SP-397/2020 EPI_ISL_583505 2020-06-26 South America / Brazil / São Paulo / Caraguatatuba Human Original Nasopharyngeal swab Ion Torrent S5 IRMA B.1.1.28 GR

hCoV-19/Brazil/SP-427/2020 EPI_ISL_603021 2020-07-15 South America / Brazil / São Paulo / Osasco Human Original Nasopharyngeal swab Ion Torrent S5 IRMA B.1.1.91 GR
hCoV-19/Brazil/SP-429/2020 EPI_ISL_603022 2020-07-16 South America / Brazil / São Paulo / Diadema Human Original Nasopharyngeal swab Ion Torrent S5 IRMA B.1.1.28 GR
hCoV-19/Brazil/SP-376/2020 EPI_ISL_603023 2020-06-07 South America / Brazil / São Paulo / Campinas Human Original Nasopharyngeal swab Ion Torrent S5 IRMA B.1.1.28 GR
hCoV-19/Brazil/SP-383/2020 EPI_ISL_603024 2020-06-20 South America / Brazil / São Paulo / Aracatuba Human Original Nasopharyngeal swab Ion Torrent S5 IRMA B.1.1.28 GR
hCoV-19/Brazil/SP-384/2020 EPI_ISL_603025 2020-06-11 South America / Brazil / São Paulo / Caraguatatuba Human Original Nasopharyngeal swab Ion Torrent S5 IRMA B.1.1.33 GR
hCoV-19/Brazil/SP-385/2020 EPI_ISL_603026 2020-06-21 South America / Brazil / São Paulo / Emilianopolis Human Original Nasopharyngeal swab Ion Torrent S5 IRMA B.1.1.33 GR
hCoV-19/Brazil/SP-387/2020 EPI_ISL_603027 2020-06-20 South America / Brazil / São Paulo / Aracatuba Human Original Nasopharyngeal swab Ion Torrent S5 IRMA B.1.1.143 GR
hCoV-19/Brazil/SP-398/2020 EPI_ISL_603028 2020-06-30 South America / Brazil / Sao Paulo / Santos Human Original Nasopharyngeal swab Ion Torrent S5 IRMA B.1.1.28 GR
hCoV-19/Brazil/SP-399/2020 EPI_ISL_603029 2020-06-22 South America / Brazil / São Paulo / Campinas Human Original Nasopharyngeal swab Ion Torrent S5 IRMA B.1.1.33 GR
hCoV-19/Brazil/SP-433/2020 EPI_ISL_603030 2020-07-11 South America / Brazil / São Paulo / Presidente Epitacio Human Original Nasopharyngeal swab Ion Torrent S5 IRMA B.1.1.28 GR
hCoV-19/Brazil/SP-434/2020 EPI_ISL_603031 2020-07-12 South America / Brazil / São Paulo / Presidente Epitacio Human Original Nasopharyngeal swab Ion Torrent S5 IRMA B.1.1.33 GR
hCoV-19/Brazil/SP-435/2020 EPI_ISL_603032 2020-07-13 South America / Brazil / São Paulo / Santo Anastacio Human Original Nasopharyngeal swab Ion Torrent S5 IRMA B.1.1.33 GR
hCoV-19/Brazil/SP-436/2020 EPI_ISL_603033 2020-07-13 South America / Brazil / São Paulo / Sao Bernardo do Campo Human Original Nasopharyngeal swab Ion Torrent S5 IRMA B.1.1.28 GR
hCoV-19/Brazil/SP-437/2020 EPI_ISL_603034 2020-07-11 South America / Brazil / São Paulo / Diadema Human Original Nasopharyngeal swab Ion Torrent S5 IRMA B.1.1.28 GR
hCoV-19/Brazil/SP-438/2020 EPI_ISL_603035 2020-07-10 South America / Brazil / São Paulo / Birigui Human Original Nasopharyngeal swab Ion Torrent S5 IRMA B.1.1.143 GR
hCoV-19/Brazil/SP-439/2020 EPI_ISL_603036 2020-07-10 South America / Brazil / São Paulo / Santo Andre Human Original Nasopharyngeal swab Ion Torrent S5 IRMA B.1.1.28 GR
hCoV-19/Brazil/SP-440/2020 EPI_ISL_603037 2020-07-20 South America / Brazil / São Paulo / São Paulo Human Original Nasopharyngeal swab Ion Torrent S5 IRMA B.1.1.28 GR
hCoV-19/Brazil/SP-441/2020 EPI_ISL_603038 2020-07-20 South America / Brazil / São Paulo / Aracatuba Human Original Nasopharyngeal swab Ion Torrent S5 IRMA B.1.1.28 GR

hCoV-19/Brazil/SP-442/2020 EPI_ISL_603039 2020-07-17 South America / Brazil / São Paulo / Campinas Human Original Nasopharyngeal swab
Ion Torrent S5 IRMA B.1.1.33 GR
hCoV-19/Brazil/RJ-00316/2020EPI_ISL_623119 2020-05-04 South America / Brazil / Rio de Janeiro Human Original Oropharyngeal swab
Illumina MiSeq BWA, Gatk, samtools, bcftools
B.1.1.33 GR
hCoV-19/Brazil/RJ-00340/2020EPI_ISL_623143 2020-05-11 South America / Brazil / Rio de Janeiro Human Original Oropharyngeal swab
Illumina MiSeq BWA, Gatk, samtools, bcftools
B.1.1.33 GR
hCoV-19/Brazil/RJ-00341/2020EPI_ISL_623144 2020-05-04 South America / Brazil / Rio de Janeiro Human Original Oropharyngeal swab
Illumina MiSeq BWA, Gatk, samtools, bcftools
B.1.1.33 GR
hCoV-19/Brazil/RJ-00342/2020EPI_ISL_623145 2020-05-18 South America / Brazil / Rio de Janeiro Human Original Oropharyngeal swab
Illumina MiSeq BWA, Gatk, samtools, bcftools
B.1.1.33 GR
hCoV-19/Brazil/RJ-00344/2020EPI_ISL_623147 2020-05-18 South America / Brazil / Rio de Janeiro Human Original Oropharyngeal swab
Illumina MiSeq BWA, Gatk, samtools, bcftools
B.1.1.33 GR
hCoV-19/Brazil/RJ-00346/2020EPI_ISL_623149 2020-05-08 South America / Brazil / Rio de Janeiro Human Original Oropharyngeal swab
Illumina MiSeq BWA, Gatk, samtools, bcftools
B.1.1.33 GR
hCoV-19/Brazil/RJ-00355/2020EPI_ISL_623158 2020-05-07 South America / Brazil / Rio de Janeiro Human Original Oropharyngeal swab
Illumina MiSeq BWA, Gatk, samtools, bcftools
B.1.1.33 GR
hCoV-19/Brazil/RJ-00358/2020EPI_ISL_623161 2020-05-04 South America / Brazil / Rio de Janeiro Human Original Oropharyngeal swab
Illumina MiSeq BWA, Gatk, samtools, bcftools
B.1.1.33 GR
hCoV-19/Brazil/RJ-00362/2020EPI_ISL_623165 2020-05-05 South America / Brazil / Rio de Janeiro Human Original Oropharyngeal swab
Illumina MiSeq BWA, Gatk, samtools, bcftools
B.1.1.314 GR
hCoV-19/Brazil/RJ-06020/2020EPI_ISL_636737 2020-05-29 South America / Brazil / Rio de Janeiro Human Original Oropharyngeal swab
MGI B.1.1.33 GR
hCoV-19/Brazil/RJ-01020R2/2020 EPI_ISL_636835 2020-05-29 South America / Brazil / Rio de Janeiro Human Original Reported case of reinfection (1st infection EPI_ISL_636834 , 2nd infection EPI_ISL_636835) MGI B.1.1.33 GR
hCoV-19/Brazil/RJ-0720R2/2020 EPI_ISL_636837 2020-05-29 South America / Brazil / Rio de Janeiro Human Original Reported case of reinfection (1st infection EPI_ISL_636836 , 2nd infection EPI_ISL_636837) MGI B.1.1.33 GR
hCoV-19/Brazil/SP-243/2020 EPI_ISL_693196 2020-05-02 South America / Brazil / São Paulo / São Paulo Human Original Nasopharyngeal swab
Ion Torrent S5 IRMA B.1.1.28 GR
hCoV-19/Brazil/SP-249/2020 EPI_ISL_693198 2020-05-04 South America / Brazil / São Paulo / São Paulo Human Original Nasopharyngeal swab
Ion Torrent S5 IRMA B.1.1.28 GR

hCoV-19/Brazil/SP-253/2020 EPI_ISL_693199 2020-05-20 South America / Brazil / São Paulo / São Paulo Human Original Nasopharyngeal swab Ion Torrent S5 IRMA B.1.1.28 GR
hCoV-19/Brazil/SP-352/2020 EPI_ISL_693204 2020-06-22 South America / Brazil / São Paulo / Osasco Human Original Nasopharyngeal swab Ion Torrent S5 IRMA B.1.1.28 GR
hCoV-19/Brazil/SP-577/2020 EPI_ISL_693205 2020-06-22 South America / Brazil / São Paulo / Taruma Human Original Nasopharyngeal swab Ion Torrent S5 IRMA B.1.1.28 GR
hCoV-19/Brazil/SP-361/2020 EPI_ISL_693206 2020-06-21 South America / Brazil / São Paulo / Campinas Human Original Nasopharyngeal swab Ion Torrent S5 IRMA B.1.1.28 GR
hCoV-19/Brazil/SP-366/2020 EPI_ISL_693207 2020-06-24 South America / Brazil / São Paulo / Sumare Human Original Nasopharyngeal swab Ion Torrent S5 IRMA B.1.1.28 GR
hCoV-19/Brazil/SP-400/2020 EPI_ISL_693208 2020-07-01 South America / Brazil / São Paulo / Osasco Human Original Nasopharyngeal swab Ion Torrent S5 IRMA B.1.1.28 GR
hCoV-19/Brazil/SP-401/2020 EPI_ISL_693209 2020-07-01 South America / Brazil / São Paulo / Osasco Human Original Nasopharyngeal swab Ion Torrent S5 IRMA B.1.1.28 GR
hCoV-19/Brazil/SP-415/2020 EPI_ISL_693210 2020-07-06 South America / Brazil / São Paulo / Osasco Human Original Nasopharyngeal swab Ion Torrent S5 IRMA B.1.1.28 GR
hCoV-19/Brazil/SP-416/2020 EPI_ISL_693211 2020-07-06 South America / Brazil / São Paulo / Cunha Human Original Nasopharyngeal swab Ion Torrent S5 IRMA B.1.1.28 GR
hCoV-19/Brazil/SP-423/2020 EPI_ISL_693212 2020-07-06 South America / Brazil / São Paulo / Braganca Paulista Human Original Nasopharyngeal swab Ion Torrent S5 IRMA B.1.1.28 GR
hCoV-19/Brazil/SP-390/2020 EPI_ISL_693213 2020-06-18 South America / Brazil / São Paulo / Hortolandia Human Original Nasopharyngeal swab Ion Torrent S5 IRMA B.1.1.33 GR
hCoV-19/Brazil/SP-579/2020 EPI_ISL_693214 2020-07-19 South America / Brazil / São Paulo / Sao Sebastiao Human Original Nasopharyngeal swab Ion Torrent S5 IRMA B.1.1.28 GR
hCoV-19/Brazil/SP-589/2020 EPI_ISL_693215 2020-07-06 South America / Brazil / São Paulo / LimeiraHuman Original Nasopharyngeal swab Ion Torrent S5 IRMA B.1.1.28 GR
hCoV-19/Brazil/SP-590/2020 EPI_ISL_693216 2020-07-05 South America / Brazil / São Paulo / Araras Human Original Nasopharyngeal swab Ion Torrent S5 IRMA B.1.1.32 GR
hCoV-19/Brazil/SP-591/2020 EPI_ISL_693217 2020-07-05 South America / Brazil / São Paulo / Araras Human Original Nasopharyngeal swab Ion Torrent S5 IRMA B.1.1.28 GR
hCoV-19/Brazil/SP-594/2020 EPI_ISL_693218 2020-06-16 South America / Brazil / São Paulo / Presidente Prudente Human Original Nasopharyngeal swab Ion Torrent S5 IRMA B.1.1.33 GR
hCoV-19/Brazil/SP-595/2020 EPI_ISL_693219 2020-06-16 South America / Brazil / São Paulo / Taciba Human Original Nasopharyngeal swab Ion Torrent S5 IRMA B.1.1.33 GR
hCoV-19/Brazil/SP-596/2020 EPI_ISL_693220 2020-07-01 South America / Brazil / São Paulo / Piracicaba Human Original Nasopharyngeal swab Ion Torrent S5 IRMA B.1.1.94 GR
hCoV-19/Brazil/SP-597/2020 EPI_ISL_693221 2020-06-16 South America / Brazil / São Paulo / BiriguiHuman Original Nasopharyngeal swab Ion Torrent S5 IRMA B.1.1.143 GR

hCoV-19/Brazil/SP-598/2020 EPI_ISL_693222 2020-06-16 South America / Brazil / São Paulo / Birigui Human Original Nasopharyngeal swab Ion Torrent S5 IRMA B.1.1.33 GR
hCoV-19/Brazil/SP-643/2020 EPI_ISL_693223 2020-07-02 South America / Brazil / São Paulo / Piracicaba Human Original Nasopharyngeal swab Ion Torrent S5 IRMA NS8_Q18stop results in 86.0% truncation of the protein sequence. info B.1.1.28 GR
hCoV-19/Brazil/SP-645/2020 EPI_ISL_693224 2020-07-02 South America / Brazil / São Paulo / Piracicaba Human Original Nasopharyngeal swab Ion Torrent S5 IRMA NS8_Q18stop results in 86.0% truncation of the protein sequence. info B.1.1.28 GR
hCoV-19/Brazil/SP-652/2020 EPI_ISL_693225 2020-07-06 South America / Brazil / São Paulo / Diadema Human Original Nasopharyngeal swab Ion Torrent S5 IRMA B.1.1.28 GR
hCoV-19/Brazil/SP-653/2020 EPI_ISL_693226 2020-06-16 South America / Brazil / São Paulo / Campinas Human Original Nasopharyngeal swab Ion Torrent S5 IRMA B.1.1.28 GR
hCoV-19/Brazil/SP-658/2020 EPI_ISL_693227 2020-07-06 South America / Brazil / São Paulo / Sao Bernardo do Campo Human Original Nasopharyngeal swab Ion Torrent S5 IRMA B.1.1.33 GR
hCoV-19/Brazil/SP-661/2020 EPI_ISL_693228 2020-06-22 South America / Brazil / São Paulo / Sorocaba Human Original Nasopharyngeal swab Ion Torrent S5 IRMA B.1.1.28 GR
hCoV-19/Brazil/SP-666/2020 EPI_ISL_693229 2020-06-16 South America / Brazil / São Paulo / Guarulhos Human Original Nasopharyngeal swab Ion Torrent S5 IRMA B.1.1.28 GR
hCoV-19/Brazil/SP-667/2020 EPI_ISL_693230 2020-06-13 South America / Brazil / São Paulo / Cotia Human Original Nasopharyngeal swab Ion Torrent S5 IRMA B.1.1.28 GR
hCoV-19/Brazil/SP-668/2020 EPI_ISL_693231 2020-06-27 South America / Brazil / São Paulo / Jacareí Human Original Nasopharyngeal swab Ion Torrent S5 IRMA B.1.1.28 GR
hCoV-19/Brazil/SP-673/2020 EPI_ISL_693232 2020-05-30 South America / Brazil / São Paulo / São Paulo Human Original Nasopharyngeal swab Ion Torrent S5 IRMA B.1.1.28 GR
hCoV-19/Brazil/SP-678/2020 EPI_ISL_693233 2020-06-12 South America / Brazil / São Paulo / Itapecerica da Serra Human Original Nasopharyngeal swab Ion Torrent S5 IRMA B.1.1.28 GR
hCoV-19/Brazil/SP-688/2020 EPI_ISL_693234 2020-06-30 South America / Brazil / São Paulo / Rio Grande da Serra Human Original Nasopharyngeal swab Ion Torrent S5 IRMA B.1.1.28 GR
hCoV-19/Brazil/SP-689/2020 EPI_ISL_693235 2020-06-30 South America / Brazil / São Paulo / Rio Grande da Serra Human Original Nasopharyngeal swab Ion Torrent S5 IRMA B.1.1.28 GR
hCoV-19/Brazil/SP-692/2020 EPI_ISL_693236 2020-07-02 South America / Brazil / São Paulo / São Paulo Human Original Nasopharyngeal swab Ion Torrent S5 IRMA B.1.1.28 GR
hCoV-19/Brazil/SP-694/2020 EPI_ISL_693237 2020-07-01 South America / Brazil / São Paulo / Mogi das Cruzes Human Original Nasopharyngeal swab Ion Torrent S5 IRMA B.1.1.28 GR
hCoV-19/Brazil/SP-696/2020 EPI_ISL_693238 2020-06-30 South America / Brazil / São Paulo / Santos Human Original Nasopharyngeal swab Ion Torrent S5 IRMA B.1.1.28 GR

hCoV-19/Brazil/SP-697/2020 EPI_ISL_693239 2020-06-30 South America / Brazil / São Paulo / Santos Human Original Nasopharyngeal swab Ion Torrent S5 IRMA B.1.1.28 GR
hCoV-19/Brazil/SP-698/2020 EPI_ISL_693240 2020-06-30 South America / Brazil / São Paulo / Diadema Human Original Nasopharyngeal swab Ion Torrent S5 IRMA B.1.1.28 GR
hCoV-19/Brazil/SP-699/2020 EPI_ISL_693241 2020-06-30 South America / Brazil / São Paulo / Ribeirao Pires Human Original Nasopharyngeal swab Ion Torrent S5 IRMA B.1.1.28 GR
hCoV-19/Brazil/SP-702/2020 EPI_ISL_693242 2020-06-30 South America / Brazil / São Paulo / Diadema Human Original Nasopharyngeal swab Ion Torrent S5 IRMA B.1.1.28 GR
hCoV-19/Brazil/SP-710/2020 EPI_ISL_693243 2020-06-27 South America / Brazil / São Paulo / Piracicaba Human Original Nasopharyngeal swab Ion Torrent S5 IRMA B.1.1.28 GR
hCoV-19/Brazil/SP-716/2020 EPI_ISL_693244 2020-07-02 South America / Brazil / São Paulo / Sorocaba Human Original Nasopharyngeal swab Ion Torrent S5 IRMA B.1.1.28 GR
hCoV-19/Brazil/SP-719/2020 EPI_ISL_693245 2020-07-02 South America / Brazil / São Paulo / Mogi das Cruzes Human Original Nasopharyngeal swab Ion Torrent S5 IRMA B.1.1.28 GR
hCoV-19/Brazil/SP-721/2020 EPI_ISL_693246 2020-06-30 South America / Brazil / São Paulo / Rio Grande da Serra Human Original Nasopharyngeal swab Ion Torrent S5 IRMA B.1.1.33 GR
hCoV-19/Brazil/SP-705/2020 EPI_ISL_693247 2020-06-30 South America / Brazil / São Paulo / Santos Human Original Nasopharyngeal swab Ion Torrent S5 IRMA NS8_E106stop results in 13.2% truncation of the protein sequence. info B.1.1.33 GR
hCoV-19/Brazil/SP-735/2020 EPI_ISL_693248 2020-07-06 South America / Brazil / São Paulo / Limeira Human Original Nasopharyngeal swab Ion Torrent S5 IRMA B.1.1.33 GR
hCoV-19/Brazil/SP-844R1/2020 EPI_ISL_708529 2020-06-29 South America / Brazil / São Paulo / Fernandopolis Human Original Nasopharyngeal swab Reported case of reinfection (1st infection EPI_ISL_708529, 2nd infection EPI_ISL_708530) Ion Torrent S5 IRMA B.1.1.33 GR
hCoV-19/Brazil/RJ-00402/2020 EPI_ISL_717806 2020-09-03 South America / Brazil / Rio de Janeiro / Rio de Janeiro Human Original Oropharyngeal swab Illumina MiSeq BWA, Gatk, samtools, bcftools B.1.1.109 GR
hCoV-19/Brazil/RJ-00407/2020 EPI_ISL_717809 2020-09-28 South America / Brazil / Rio de Janeiro / Maricá Human Original Oropharyngeal swab Illumina MiSeq BWA, Gatk, samtools, bcftools B.1.1.31 GR
hCoV-19/Brazil/RJ-00410/2020 EPI_ISL_717812 2020-06-01 South America / Brazil / Rio de Janeiro / Rio de Janeiro Human Original Oropharyngeal swab Illumina MiSeq BWA, Gatk, samtools, bcftools B.1.1.28 GR
hCoV-19/Brazil/RJ-00411/2020 EPI_ISL_717813 2020-06-01 South America / Brazil / Rio de Janeiro / Rio de Janeiro Human Original Oropharyngeal swab Illumina MiSeq BWA, Gatk, samtools, bcftools B.1.1.28 GR
hCoV-19/Brazil/RJ-00412/2020 EPI_ISL_717814 2020-09-03 South America / Brazil / Rio de Janeiro / Nilópolis Human Original Oropharyngeal swab Illumina MiSeq BWA, Gatk, samtools, bcftools B.1.1.28 GR

hCoV-19/Brazil/RJ-00413/2020EPI_ISL_717815 2020-09-03 South America / Brazil / Rio de Janeiro / Rio de Janeiro Human Original Oropharyngeal swab Illumina MiSeq BWA, Gatk, samtools, bcftools B.1.1.28 GR
hCoV-19/Brazil/RJ-00414/2020EPI_ISL_717816 2020-09-03 South America / Brazil / Rio de Janeiro / Rio de Janeiro Human Original Oropharyngeal swab Illumina MiSeq BWA, Gatk, samtools, bcftools B.1.1.28 GR
hCoV-19/Brazil/RJ-00415/2020EPI_ISL_717817 2020-09-04 South America / Brazil / Rio de Janeiro / Rio de Janeiro Human Original Oropharyngeal swab Illumina MiSeq BWA, Gatk, samtools, bcftools B.1.1.28 GR
hCoV-19/Brazil/RJ-00437/2020EPI_ISL_717832 2020-06-01 South America / Brazil / Rio de Janeiro / Maricá Human Original Oropharyngeal swab Illumina MiSeq BWA, Gatk, samtools, bcftools B.1.1.33 GR
hCoV-19/Brazil/RJ-00438/2020EPI_ISL_717833 2020-06-03 South America / Brazil / Rio de Janeiro / Maricá Human Original Oropharyngeal swab Illumina MiSeq BWA, Gatk, samtools, bcftools B.1.1.33 GR
hCoV-19/Brazil/RJ-00439/2020EPI_ISL_717834 2020-06-02 South America / Brazil / Rio de Janeiro / Maricá Human Original Oropharyngeal swab Illumina MiSeq BWA, Gatk, samtools, bcftools B.1.1.33 GR
hCoV-19/Brazil/RJ-00440/2020EPI_ISL_717835 2020-06-03 South America / Brazil / Rio de Janeiro / Maricá Human Original Oropharyngeal swab Illumina MiSeq BWA, Gatk, samtools, bcftools B.1.1.33 GR
hCoV-19/Brazil/RJ-00441/2020EPI_ISL_717836 2020-06-03 South America / Brazil / Rio de Janeiro / Maricá Human Original Oropharyngeal swab Illumina MiSeq BWA, Gatk, samtools, bcftools B.1.1.33 GR
hCoV-19/Brazil/RJ-00446/2020EPI_ISL_717841 2020-07-28 South America / Brazil / Rio de Janeiro / Maricá Human Original Oropharyngeal swab Illumina MiSeq BWA, Gatk, samtools, bcftools B.1.1.33 GR
hCoV-19/Brazil/RJ-00450/2020EPI_ISL_717845 2020-06-01 South America / Brazil / Rio de Janeiro / Nova Iguaçu Human Original Oropharyngeal swab Illumina MiSeq BWA, Gatk, samtools, bcftools B.1.1.33 GR
hCoV-19/Brazil/RJ-00451/2020EPI_ISL_717846 2020-06-01 South America / Brazil / Rio de Janeiro / Rio de Janeiro Human Original Oropharyngeal swab Illumina MiSeq BWA, Gatk, samtools, bcftools B.1.1.33 GR
hCoV-19/Brazil/RJ-00452/2020EPI_ISL_717847 2020-06-01 South America / Brazil / Rio de Janeiro / Belford Roxo Human Original Oropharyngeal swab Illumina MiSeq BWA, Gatk, samtools, bcftools B.1.1.33 GR
hCoV-19/Brazil/RJ-00453/2020EPI_ISL_717848 2020-06-01 South America / Brazil / Rio de Janeiro / Seropédica Human Original Oropharyngeal swab Illumina MiSeq BWA, Gatk, samtools, bcftools B.1.1.33 GR
hCoV-19/Brazil/RJ-00454/2020EPI_ISL_717849 2020-06-01 South America / Brazil / Rio de Janeiro / Nilópolis Human Original Oropharyngeal swab Illumina MiSeq BWA, Gatk, samtools, bcftools B.1.1.33 GR
hCoV-19/Brazil/RJ-00455/2020EPI_ISL_717850 2020-06-01 South America / Brazil / Rio de Janeiro / Itaguaí Human Original Oropharyngeal swab

Illumina MiSeq BWA, Gatk, samtools, bcftools
B.1.1.33 GR
hCoV-19/Brazil/RJ-00456/2020EPI_ISL_717851 2020-06-01 South America / Brazil / Rio de Janeiro / Rio de Janeiro Human Original
Oropharyngeal swab Illumina MiSeq BWA, Gatk, samtools, bcftools B.1.1.33 GR
hCoV-19/Brazil/RJ-00457/2020EPI_ISL_717852 2020-06-01 South America / Brazil / Rio de Janeiro / Rio de Janeiro Human Original
Oropharyngeal swab Illumina MiSeq BWA, Gatk, samtools, bcftools B.1.1.33 GR
hCoV-19/Brazil/RJ-00458/2020EPI_ISL_717853 2020-07-01 South America / Brazil / Rio de Janeiro / Rio de Janeiro Human Original
Oropharyngeal swab Illumina MiSeq BWA, Gatk, samtools, bcftools B.1.1.33 GR
hCoV-19/Brazil/RJ-00459/2020EPI_ISL_717854 2020-07-01 South America / Brazil / Rio de Janeiro / Rio de Janeiro Human Original
Oropharyngeal swab Illumina MiSeq BWA, Gatk, samtools, bcftools B.1.1.33 GR
hCoV-19/Brazil/RJ-00460/2020EPI_ISL_717855 2020-07-01 South America / Brazil / Rio de Janeiro / Rio de Janeiro Human Original
Oropharyngeal swab Illumina MiSeq BWA, Gatk, samtools, bcftools B.1.1.33 GR
hCoV-19/Brazil/RJ-00461/2020EPI_ISL_717856 2020-07-02 South America / Brazil / Rio de Janeiro / Rio de Janeiro Human Original
Oropharyngeal swab Illumina MiSeq BWA, Gatk, samtools, bcftools B.1.1.33 GR
hCoV-19/Brazil/RJ-00462/2020EPI_ISL_717857 2020-07-02 South America / Brazil / Rio de Janeiro / Rio de Janeiro Human Original
Oropharyngeal swab Illumina MiSeq BWA, Gatk, samtools, bcftools B.1.1.33 GR
hCoV-19/Brazil/RJ-00463/2020EPI_ISL_717858 2020-07-02 South America / Brazil / Rio de Janeiro / Rio de Janeiro Human Original
Oropharyngeal swab Illumina MiSeq BWA, Gatk, samtools, bcftools B.1.1.33 GR
hCoV-19/Brazil/RJ-00464/2020EPI_ISL_717859 2020-07-03 South America / Brazil / Rio de Janeiro / Rio de Janeiro Human Original
Oropharyngeal swab Illumina MiSeq BWA, Gatk, samtools, bcftools B.1.1.33 GR
hCoV-19/Brazil/RJ-00465/2020EPI_ISL_717860 2020-07-06 South America / Brazil / Rio de Janeiro / Rio de Janeiro Human Original
Oropharyngeal swab Illumina MiSeq BWA, Gatk, samtools, bcftools B.1.1.33 GR
hCoV-19/Brazil/RJ-00466/2020EPI_ISL_717861 2020-07-08 South America / Brazil / Rio de Janeiro / Rio de Janeiro Human Original
Oropharyngeal swab Illumina MiSeq BWA, Gatk, samtools, bcftools B.1.1.33 GR
hCoV-19/Brazil/RJ-00467/2020EPI_ISL_717862 2020-07-08 South America / Brazil / Rio de Janeiro / Rio de Janeiro Human Original
Oropharyngeal swab Illumina MiSeq BWA, Gatk, samtools, bcftools B.1.1.33 GR
hCoV-19/Brazil/RJ-00468/2020EPI_ISL_717863 2020-08-03 South America / Brazil / Rio de Janeiro / São João de Meriti Human Original
Oropharyngeal swab Illumina MiSeq BWA, Gatk, samtools, bcftools B.1.1.33 GR
hCoV-19/Brazil/RJ-00469/2020EPI_ISL_717864 2020-08-03 South America / Brazil / Rio de Janeiro / Rio de Janeiro Human Original
Oropharyngeal swab Illumina MiSeq BWA, Gatk, samtools, bcftools B.1.1.33 GR

hCoV-19/Brazil/RJ-00470/2020EPI_ISL_717865 2020-08-03 South America / Brazil / Rio de Janeiro / Guapimirim Human Original Oropharyngeal swab Illumina MiSeq BWA, Gatk, samtools, bcftools
B.1.1.33 GR
hCoV-19/Brazil/RJ-00471/2020EPI_ISL_717866 2020-08-03 South America / Brazil / Rio de Janeiro / Guapimirim Human Original Oropharyngeal swab Illumina MiSeq BWA, Gatk, samtools, bcftools
B.1.1.33 GR
hCoV-19/Brazil/RJ-00472/2020EPI_ISL_717867 2020-08-04 South America / Brazil / Rio de Janeiro / Rio de Janeiro Human Original
Oropharyngeal swab Illumina MiSeq BWA, Gatk, samtools, bcftools B.1.1.33 GR
hCoV-19/Brazil/RJ-00473/2020EPI_ISL_717868 2020-08-05 South America / Brazil / Rio de Janeiro / São João de Meriti Human Original
Oropharyngeal swab Illumina MiSeq BWA, Gatk, samtools, bcftools B.1.1.33 GR
hCoV-19/Brazil/RJ-00474/2020EPI_ISL_717869 2020-09-03 South America / Brazil / Rio de Janeiro / Rio de Janeiro Human Original
Oropharyngeal swab Illumina MiSeq BWA, Gatk, samtools, bcftools B.1.1.33 GR
hCoV-19/Brazil/RJ-00475/2020EPI_ISL_717870 2020-09-04 South America / Brazil / Rio de Janeiro / Rio de Janeiro Human Original
Oropharyngeal swab Illumina MiSeq BWA, Gatk, samtools, bcftools B.1.1.33 GR
hCoV-19/Brazil/RJ-00476/2020EPI_ISL_717871 2020-09-04 South America / Brazil / Rio de Janeiro / Rio de Janeiro Human Original
Oropharyngeal swab Illumina MiSeq BWA, Gatk, samtools, bcftools B.1.1.33 GR
hCoV-19/Brazil/RJ-00477/2020EPI_ISL_717872 2020-09-04 South America / Brazil / Rio de Janeiro / Rio de Janeiro Human Original
Oropharyngeal swab Illumina MiSeq BWA, Gatk, samtools, bcftools B.1.1.33 GR
hCoV-19/Brazil/RJ-00478/2020EPI_ISL_717873 2020-09-08 South America / Brazil / Rio de Janeiro / Rio de Janeiro Human Original
Oropharyngeal swab Illumina MiSeq BWA, Gatk, samtools, bcftools B.1.1.33 GR
hCoV-19/Brazil/RJ-00505/2020EPI_ISL_717899 2020-07-27 South America / Brazil / Rio de Janeiro / Rio de Janeiro Human Original
Oropharyngeal swab Illumina MiSeq BWA, Gatk, samtools, bcftools B.1.1.314 GR
hCoV-19/Brazil/RJ-00507/2020EPI_ISL_717900 2020-07-27 South America / Brazil / Rio de Janeiro / Rio de Janeiro Human Original
Oropharyngeal swab Illumina MiSeq BWA, Gatk, samtools, bcftools B.1.1.33 GR
hCoV-19/Brazil/RJ-00508/2020EPI_ISL_717901 2020-07-30 South America / Brazil / Rio de Janeiro / Rio de Janeiro Human Original
Oropharyngeal swab Illumina MiSeq BWA, Gatk, samtools, bcftools B.1.1.33 GR
hCoV-19/Brazil/RJ-00511/2020EPI_ISL_717902 2020-07-27 South America / Brazil / Rio de Janeiro / São Gonçalo Human Original Oropharyngeal swab Illumina MiSeq BWA, Gatk, samtools, bcftools
B.1.1.33 GR
hCoV-19/Brazil/RJ-00512/2020EPI_ISL_717903 2020-07-28 South America / Brazil / Rio de Janeiro / Macaé Human Original Oropharyngeal swab
Illumina MiSeq BWA, Gatk, samtools, bcftools
B.1.1.33 GR
hCoV-19/Brazil/RJ-00515/2020EPI_ISL_717904 2020-07-28 South America / Brazil / Rio de Janeiro / Nova Friburgo Human Original Oropharyngeal

swab Illumina MiSeq BWA, Gatk, samtools, bcftools
B.1.1.33 GR
hCoV-19/Brazil/RJ-00516/2020EPI_ISL_717905 2020-07-28 South America / Brazil / Rio de Janeiro / Petrópolis Human Original Oropharyngeal swab Illumina MiSeq BWA, Gatk, samtools, bcftools
B.1.1.33 GR
hCoV-19/Brazil/RJ-00517/2020EPI_ISL_717906 2020-07-29 South America / Brazil / Rio de Janeiro / Petrópolis Human Original Oropharyngeal swab Illumina MiSeq BWA, Gatk, samtools, bcftools
B.1.1.33 GR
hCoV-19/Brazil/RJ-00518/2020EPI_ISL_717907 2020-07-18 South America / Brazil / Rio de Janeiro / Três Rios Human Original Oropharyngeal swab Illumina MiSeq BWA, Gatk, samtools, bcftools
B.1.1.33 GR
hCoV-19/Brazil/RJ-00520/2020EPI_ISL_717908 2020-08-01 South America / Brazil / Rio de Janeiro / Três Rios Human Original Oropharyngeal swab Illumina MiSeq BWA, Gatk, samtools, bcftools
B.1.1.33 GR
hCoV-19/Brazil/RJ-00522/2020EPI_ISL_717909 2020-07-29 South America / Brazil / Rio de Janeiro / Nova Iguaçu Human Original Oropharyngeal swab Illumina MiSeq BWA, Gatk, samtools, bcftools
B.1.1.33 GR
hCoV-19/Brazil/RJ-00524/2020EPI_ISL_717910 2020-05-31 South America / Brazil / Rio de Janeiro / Maricá Human Original Oropharyngeal swab
Illumina MiSeq BWA, Gatk, samtools, bcftools
B.1.1.33 GR
hCoV-19/Brazil/RJ-00525/2020EPI_ISL_717911 2020-06-01 South America / Brazil / Rio de Janeiro / Maricá Human Original Oropharyngeal swab
Illumina MiSeq BWA, Gatk, samtools, bcftools
B.1.1.33 GR
hCoV-19/Brazil/RJ-00526/2020EPI_ISL_717912 2020-06-02 South America / Brazil / Rio de Janeiro / Maricá Human Original Oropharyngeal swab
Illumina MiSeq BWA, Gatk, samtools, bcftools
B.1.1.33 GR
hCoV-19/Brazil/RJ-00527/2020EPI_ISL_717913 2020-07-29 South America / Brazil / Rio de Janeiro / Maricá Human Original Oropharyngeal swab
Illumina MiSeq BWA, Gatk, samtools, bcftools
B.1.1.33 GR
hCoV-19/Brazil/RJ-00528/2020EPI_ISL_717914 2020-07-29 South America / Brazil / Rio de Janeiro / Maricá Human Original Oropharyngeal swab
Illumina MiSeq BWA, Gatk, samtools, bcftools
B.1.1.33 GR
hCoV-19/Brazil/RJ-00529/2020EPI_ISL_717915 2020-07-29 South America / Brazil / Rio de Janeiro / Maricá Human Original Oropharyngeal swab
Illumina MiSeq BWA, Gatk, samtools, bcftools
B.1.1.33 GR
hCoV-19/Brazil/RJ-00530/2020EPI_ISL_717916 2020-07-29 South America / Brazil / Rio de Janeiro / Maricá Human Original Oropharyngeal swab
Illumina MiSeq BWA, Gatk, samtools, bcftools
B.1.1.33 GR
hCoV-19/Brazil/RJ-00531/2020EPI_ISL_717917 2020-07-29 South America / Brazil / Rio de Janeiro / Maricá Human Original Oropharyngeal swab
Illumina MiSeq BWA, Gatk, samtools, bcftools
B.1.1.33 GR
hCoV-19/Brazil/RJ-00532/2020EPI_ISL_717918 2020-06-02 South America / Brazil / Rio de Janeiro / Maricá Human Original Oropharyngeal swab
Illumina MiSeq BWA, Gatk, samtools, bcftools
B.1.1.33 GR

hCoV-19/Brazil/RJ-00533/2020EPI_ISL_717919 2020-06-02 South America / Brazil / Rio de Janeiro / Maricá Human Original Oropharyngeal swab Illumina MiSeq BWA, Gatk, samtools, bcftools B.1.1.33 GR
hCoV-19/Brazil/RJ-00574/2020EPI_ISL_717958 2020-07-28 South America / Brazil / Rio de Janeiro / Maricá Human Original Oropharyngeal swab Illumina MiSeq BWA, Gatk, samtools, bcftools B.1.1.33 GR
hCoV-19/Brazil/RJ-00575/2020EPI_ISL_717959 2020-08-03 South America / Brazil / Rio de Janeiro / Rio de Janeiro Human Original Oropharyngeal swab Illumina MiSeq BWA, Gatk, samtools, bcftools B.1.1.33 GR
hCoV-19/Brazil/RJ-00578/2020EPI_ISL_717962 2020-07-28 South America / Brazil / Rio de Janeiro / Macaé Human Original Oropharyngeal swab Illumina MiSeq BWA, Gatk, samtools, bcftools B.1.1.33 GR
hCoV-19/Brazil/RJ-00579/2020EPI_ISL_717963 2020-07-29 South America / Brazil / Rio de Janeiro / Maricá Human Original Oropharyngeal swab Illumina MiSeq BWA, Gatk, samtools, bcftools B.1.1.33 GR
hCoV-19/Brazil/RJ-00580/2020EPI_ISL_717964 2020-07-29 South America / Brazil / Rio de Janeiro / Maricá Human Original Oropharyngeal swab Illumina MiSeq BWA, Gatk, samtools, bcftools B.1.1.33 GR
hCoV-19/Brazil/RS-FIOCRUZ-6195/2020 EPI_ISL_729794 2020-05-11 South America / Brazil / Rio Grande do Sul / Nova Araça Human Original Nasopharyngeal swab Illumina MiSeq CLC Genomics Workbench NS8_K68stop results in 44.6% truncation of the protein sequence. info B.1.1.33 GR
hCoV-19/Brazil/RS-FIOCRUZ-6228/2020 EPI_ISL_729800 2020-05-28 South America / Brazil / Rio Grande do Sul / Lajeado Human Original Nasopharyngeal swab Illumina MiSeq CLC Genomics Workbench B.1.1.33 GR
hCoV-19/Brazil/RS-FIOCRUZ-15270/2020 EPI_ISL_729801 2020-07-13 South America / Brazil / Rio Grande do Sul / Passo Fundo Human Original Nasopharyngeal swab Illumina MiSeq CLC Genomics Workbench B.1.1.28 GR
hCoV-19/Brazil/RS-FIOCRUZ-15283/2020 EPI_ISL_729802 2020-08-09 South America / Brazil / Rio Grande do Sul / Porto Alegre Human Original Nasopharyngeal swab Illumina MiSeq CLC Genomics Workbench B.1.1.33 GR
hCoV-19/Brazil/RS-FIOCRUZ-15286/2020 EPI_ISL_729803 2020-08-14 South America / Brazil / Rio Grande do Sul / Sao Borja Human Original Nasopharyngeal swab Illumina MiSeq CLC Genomics Workbench B.1.1.28 GR
hCoV-19/Brazil/RS-FIOCRUZ-6177/2020 EPI_ISL_729820 2020-05-06 South America / Brazil / Rio Grande do Sul / Carlos Barbosa Human Original Nasopharyngeal swab Illumina MiSeq CLC Genomics Workbench B.1.1.33 GR
hCoV-19/Brazil/RS-FIOCRUZ-6179/2020 EPI_ISL_729821 2020-05-06 South America / Brazil / Rio Grande do Sul / Santa Rosa Human Original Nasopharyngeal swab Illumina MiSeq CLC Genomics Workbench B.1.1.33 GR
hCoV-19/Brazil/RS-FIOCRUZ-6183/2020 EPI_ISL_729822 2020-05-06 South America / Brazil / Rio Grande do Sul / Saldanha Marinho Human Original Nasopharyngeal swab Illumina MiSeq CLC Genomics Workbench B.1.1.29 GR

hCoV-19/Brazil/RS-FIOCRUZ-6184/2020 EPI_ISL_729823 2020-05-08 South America / Brazil / Rio Grande do Sul / Itaqui Human Original Nasopharyngeal swab Illumina MiSeq CLC Genomics Workbench B.1.1.33 GR
hCoV-19/Brazil/RS-FIOCRUZ-6188/2020 EPI_ISL_729824 2020-05-10 South America / Brazil / Rio Grande do Sul / Caxias Do Sul Human Original Nasopharyngeal swab Illumina MiSeq CLC Genomics Workbench B.1.1.10 GR
hCoV-19/Brazil/RS-FIOCRUZ-6189/2020 EPI_ISL_729825 2020-05-10 South America / Brazil / Rio Grande do Sul / Caxias Do Sul Human Original Nasopharyngeal swab Illumina MiSeq CLC Genomics Workbench B.1.1.33 GR
hCoV-19/Brazil/RS-FIOCRUZ-6190/2020 EPI_ISL_729826 2020-05-11 South America / Brazil / Rio Grande do Sul / Arroio Do Meio Human Original Nasopharyngeal swab Illumina MiSeq CLC Genomics Workbench B.1.1.33 GR
hCoV-19/Brazil/RS-FIOCRUZ-6192/2020 EPI_ISL_729827 2020-05-12 South America / Brazil / Rio Grande do Sul / Serafina Correa Human Original Nasopharyngeal swab Illumina MiSeq CLC Genomics Workbench B.1.1.33 GR
hCoV-19/Brazil/RS-FIOCRUZ-6196/2020 EPI_ISL_729828 2020-05-12 South America / Brazil / Rio Grande do Sul / Passo Fundo Human Original Nasopharyngeal swab Illumina MiSeq CLC Genomics Workbench B.1.1.33 GR
hCoV-19/Brazil/RS-FIOCRUZ-6197/2020 EPI_ISL_729829 2020-05-13 South America / Brazil / Rio Grande do Sul / Nao-Me-Toque Human Original Nasopharyngeal swab Illumina MiSeq CLC Genomics Workbench B.1.1.33 GR
hCoV-19/Brazil/RS-FIOCRUZ-6198/2020 EPI_ISL_729830 2020-05-14 South America / Brazil / Rio Grande do Sul / Serafina Correa Human Original Nasopharyngeal swab Illumina MiSeq CLC Genomics Workbench B.1.1.33 GR
hCoV-19/Brazil/RS-FIOCRUZ-6203/2020 EPI_ISL_729831 2020-05-14 South America / Brazil / Rio Grande do Sul / Quarai Human Original Nasopharyngeal swab Illumina MiSeq CLC Genomics Workbench B.1.1.33 GR
hCoV-19/Brazil/RS-FIOCRUZ-6205/2020 EPI_ISL_729832 2020-05-18 South America / Brazil / Rio Grande do Sul / Santana Do Livramento Human Original Nasopharyngeal swab Illumina MiSeq CLC Genomics Workbench B.1.1.33 GR
hCoV-19/Brazil/RS-FIOCRUZ-6208/2020 EPI_ISL_729833 2020-05-19 South America / Brazil / Rio Grande do Sul / Frederico Westphalen Human Original Nasopharyngeal swab Illumina MiSeq CLC Genomics Workbench B.1.1.33 GR
hCoV-19/Brazil/RS-FIOCRUZ-6215/2020 EPI_ISL_729835 2020-05-20 South America / Brazil / Rio Grande do Sul / Passo Fundo Human Original Nasopharyngeal swab Illumina MiSeq CLC Genomics Workbench B.1.1.29 GR
hCoV-19/Brazil/RS-FIOCRUZ-6218/2020 EPI_ISL_729836 2020-05-27 South America / Brazil / Rio Grande do Sul / Porto Alegre Human Original Nasopharyngeal swab Illumina MiSeq CLC Genomics Workbench B.1.91 G
hCoV-19/Brazil/RS-FIOCRUZ-6222/2020 EPI_ISL_729837 2020-05-28 South America / Brazil / Rio Grande do Sul / Flores Da Cunha Human Original Nasopharyngeal swab Illumina MiSeq CLC Genomics Workbench B.1.1.33 GR
hCoV-19/Brazil/RS-FIOCRUZ-6227/2020 EPI_ISL_729838 2020-05-29 South America / Brazil / Rio Grande do Sul / Venancio Aires Human Original

Nasopharyngeal swab Illumina MiSeq CLC Genomics Workbench
B.1.1.33 GR
hCoV-19/Brazil/RS-FIOCRUZ-6231/2020 EPI_ISL_729839 2020-05-29 South America / Brazil / Rio Grande do Sul / Cacapava Do Sul Human Original
Nasopharyngeal swab Illumina MiSeq CLC Genomics Workbench
B.1.1.33 GR
hCoV-19/Brazil/RS-FIOCRUZ-6232/2020 EPI_ISL_729840 2020-05-28 South America / Brazil / Rio Grande do Sul / Lavras Do Sul Human Original
Nasopharyngeal swab Illumina MiSeq CLC Genomics Workbench
B.1.1.28 GR
hCoV-19/Brazil/RS-FIOCRUZ-6240/2020 EPI_ISL_729841 2020-06-01 South America / Brazil / Rio Grande do Sul / Santo Angelo Human Original
Nasopharyngeal swab Illumina MiSeq CLC Genomics Workbench
B.1.1.33 GR
hCoV-19/Brazil/RS-FIOCRUZ-6241/2020 EPI_ISL_729842 2020-06-01 South America / Brazil / Rio Grande do Sul / Girua Human Original
Nasopharyngeal swab Illumina MiSeq CLC Genomics Workbench
B.1.1.33 GR
hCoV-19/Brazil/RS-FIOCRUZ-6242/2020 EPI_ISL_729843 2020-05-31 South America / Brazil / Rio Grande do Sul / Cruz Alta Human Original
Nasopharyngeal swab Illumina MiSeq CLC Genomics Workbench
B.1.1.33 GR
hCoV-19/Brazil/RS-FIOCRUZ-15273/2020 EPI_ISL_729844 2020-07-17 South America / Brazil / Rio Grande do Sul / Cachoeira Do Sul Human Original
Nasopharyngeal swab Illumina MiSeq CLC Genomics Workbench
B.1.1.33 GR
hCoV-19/Brazil/RS-FIOCRUZ-15279/2020 EPI_ISL_729845 2020-07-28 South America / Brazil / Rio Grande do Sul / Nova Brescia Human Original
Nasopharyngeal swab Illumina MiSeq CLC Genomics Workbench
B.1.1.279 GR
hCoV-19/Brazil/RS-FIOCRUZ-6226/2020 EPI_ISL_729846 2020-05-29 South America / Brazil / Rio Grande do Sul / Porto Alegre Human Original
Nasopharyngeal swab IonTorrent CLC Genomics Workbench
B.1.91 G
hCoV-19/Brazil/RS-FIOCRUZ-6243/2020 EPI_ISL_729847 2020-06-02 South America / Brazil / Rio Grande do Sul / Porto Alegre Human Original
Nasopharyngeal swab IonTorrent CLC Genomics Workbench
B.1.91 G
hCoV-19/Brazil/RS-FIOCRUZ-15278/2020 EPI_ISL_729848 2020-07-27 South America / Brazil / Rio Grande do Sul / Tapejara Human Original
Nasopharyngeal swab Nanopore MinION CLC Genomics Workbench
B.1.1.29 GR
hCoV-19/Brazil/RS-FIOCRUZ-15288/2020 EPI_ISL_729849 2020-08-12 South America / Brazil / Rio Grande do Sul / Carazinho Human Original
Nasopharyngeal swab Nanopore MinION CLC Genomics Workbench
B.1.1.33 GR
hCoV-19/Brazil/RS-FIOCRUZ-15274/2020 EPI_ISL_729850 2020-07-17 South America / Brazil / Rio Grande do Sul / Porto Alegre Human Original
Nasopharyngeal swab Nanopore MinION CLC Genomics Workbench
B.1.91 G
hCoV-19/Brazil/RS-FIOCRUZ-15280/2020 EPI_ISL_729851 2020-07-28 South America / Brazil / Rio Grande do Sul / Porto Alegre Human Original
Nasopharyngeal swab Nanopore MinION CLC Genomics Workbench
B.1.1.33 GR
hCoV-19/Brazil/RS-FIOCRUZ-15281/2020 EPI_ISL_729852 2020-08-07 South America / Brazil / Rio Grande do Sul / Farroupilha Human Original
Nasopharyngeal swab Nanopore MinION CLC Genomics Workbench
B.1.1.28 GR

hCoV-19/Brazil/RS-FIOCRUZ-15284/2020 EPI_ISL_729853 2020-08-12 South America / Brazil / Rio Grande do Sul / Ijui Human Original Nasopharyngeal swab Nanopore MinION CLC Genomics Workbench B.1.1.28 GR
hCoV-19/Brazil/RS-FIOCRUZ-15289/2020 EPI_ISL_729854 2020-08-14 South America / Brazil / Rio Grande do Sul / Porto Alegre Human Original Nasopharyngeal swab Nanopore MinION CLC Genomics Workbench B.1.1.28 GR
hCoV-19/Brazil/RS-FIOCRUZ-15291/2020 EPI_ISL_729855 2020-08-15 South America / Brazil / Rio Grande do Sul / Tres Passos Human Original Nasopharyngeal swab Nanopore MinION CLC Genomics Workbench B.1.1.29 GR
hCoV-19/Brazil/RS-FIOCRUZ-15275/2020 EPI_ISL_729856 2020-07-18 South America / Brazil / Rio Grande do Sul / Pelotas Human Original Nasopharyngeal swab Nanopore MinION CLC Genomics Workbench B.1.1.28 GR
hCoV-19/Brazil/RS-FIOCRUZ-15276/2020 EPI_ISL_729857 2020-07-20 South America / Brazil / Rio Grande do Sul / Bom Retiro Do Sul Human Original Nasopharyngeal swab Nanopore MinION CLC Genomics Workbench B.1.1.33 GR
hCoV-19/Brazil/RS-FIOCRUZ-15282/2020 EPI_ISL_729858 2020-08-06 South America / Brazil / Rio Grande do Sul / Caxias Do Sul Human Original Nasopharyngeal swab Nanopore MinION CLC Genomics Workbench B.1.1.33 GR
hCoV-19/Brazil/RS-FIOCRUZ-15285/2020 EPI_ISL_729859 2020-08-14 South America / Brazil / Rio Grande do Sul / Venancio Aires Human Original Nasopharyngeal swab Nanopore MinION CLC Genomics Workbench B.1.1.29 GR
hCoV-19/Brazil/RS-FIOCRUZ-15287/2020 EPI_ISL_729860 2020-08-13 South America / Brazil / Rio Grande do Sul / Santa Rosa Human Original Nasopharyngeal swab Nanopore MinION CLC Genomics Workbench B.1.1.33 GR
hCoV-19/Brazil/RS-FIOCRUZ-15292/2020 EPI_ISL_729861 2020-08-16 South America / Brazil / Rio Grande do Sul / Porto Alegre Human Original Nasopharyngeal swab Nanopore MinION CLC Genomics Workbench B.1.1.28 GR
hCoV-19/Brazil/SP-585/2020 EPI_ISL_735396 2020-07-05 South America / Brazil / São Paulo / Taboao da Serra Human Original Nasopharyngeal swab Ion Torrent S5 IRMA B.1.1.28 GR
hCoV-19/Brazil/SP-586/2020 EPI_ISL_735397 2020-07-09 South America / Brazil / São Paulo / Hortolandia Human Original Nasopharyngeal swab Ion Torrent S5 IRMA B.1.1.28 GR
hCoV-19/Brazil/SP-737/2020 EPI_ISL_735398 2020-07-09 South America / Brazil / São Paulo / São Paulo Human Original Nasopharyngeal swab Ion Torrent S5 IRMA B.1.1.28 GR
hCoV-19/Brazil/SP-739/2020 EPI_ISL_735400 2020-07-09 South America / Brazil / São Paulo / Santos Human Original Nasopharyngeal swab Ion Torrent S5 IRMA B.1.1.28 GR
hCoV-19/Brazil/SP-740/2020 EPI_ISL_735401 2020-07-04 South America / Brazil / São Paulo / Rio Claro Human Original Nasopharyngeal swab Ion Torrent S5 IRMA B.1.1.28 GR
hCoV-19/Brazil/SP-742/2020 EPI_ISL_735402 2020-07-04 South America / Brazil / São Paulo / Rio Claro Human Original Nasopharyngeal swab Ion Torrent S5 IRMA B.1.1.28 GR
hCoV-19/Brazil/SP-743/2020 EPI_ISL_735403 2020-07-04 South America / Brazil / São Paulo / Rio Claro Human Original Nasopharyngeal swab Ion Torrent S5 IRMA B.1.1.28 GR

hCoV-19/Brazil/SP-744/2020 EPI_ISL_735404 2020-07-04 South America / Brazil / São Paulo / Rio Claro Human Original Nasopharyngeal swab Ion Torrent S5 IRMA B.1.1.28 GR
hCoV-19/Brazil/SP-745/2020 EPI_ISL_735405 2020-06-16 South America / Brazil / São Paulo / Birigui Human Original Nasopharyngeal swab Ion Torrent S5 IRMA B.1.1.143 GR
hCoV-19/Brazil/SP-748/2020 EPI_ISL_735406 2020-07-08 South America / Brazil / São Paulo / Santa Isabel Human Original Nasopharyngeal swab Ion Torrent S5 IRMA B.1.1.28 GR
hCoV-19/Brazil/SP-749/2020 EPI_ISL_735407 2020-06-26 South America / Brazil / São Paulo / Marilia Human Original Nasopharyngeal swab Ion Torrent S5 IRMA B.1.1.33 GR
hCoV-19/Brazil/SP-750/2020 EPI_ISL_735408 2020-06-25 South America / Brazil / São Paulo / Jandira Human Original Nasopharyngeal swab Ion Torrent S5 IRMA B.1.1.28 GR
hCoV-19/Brazil/SP-751/2020 EPI_ISL_735409 2020-06-17 South America / Brazil / São Paulo / Campinas Human Original Nasopharyngeal swab Ion Torrent S5 IRMA B.1.1.28 GR
hCoV-19/Brazil/SP-754/2020 EPI_ISL_735410 2020-07-03 South America / Brazil / São Paulo / Rio Claro Human Original Nasopharyngeal swab Ion Torrent S5 IRMA B.1.1.33 GR
hCoV-19/Brazil/SP-756/2020 EPI_ISL_735411 2020-07-06 South America / Brazil / São Paulo / Diadema Human Original Nasopharyngeal swab Ion Torrent S5 IRMA B.1.1.28 GR
hCoV-19/Brazil/SP-758/2020 EPI_ISL_735412 2020-06-15 South America / Brazil / São Paulo / São Paulo Human Original Nasopharyngeal swab Ion Torrent S5 IRMA B.1.1.28 GR
hCoV-19/Brazil/SP-759/2020 EPI_ISL_735413 2020-06-17 South America / Brazil / São Paulo / São Paulo Human Original Nasopharyngeal swab Ion Torrent S5 IRMA B.1.1.28 GR
hCoV-19/Brazil/SP-761/2020 EPI_ISL_735414 2020-06-26 South America / Brazil / São Paulo / Mongagua Human Original Nasopharyngeal swab Ion Torrent S5 IRMA B.1.1.33 GR
hCoV-19/Brazil/SP-762/2020 EPI_ISL_735415 2020-06-25 South America / Brazil / São Paulo / Mongagua Human Original Nasopharyngeal swab Ion Torrent S5 IRMA B.1.1.33 GR
hCoV-19/Brazil/SP-764/2020 EPI_ISL_735416 2020-06-26 South America / Brazil / São Paulo / Mococa Human Original Nasopharyngeal swab Ion Torrent S5 IRMA B.1.1.33 GR
hCoV-19/Brazil/SP-765/2020 EPI_ISL_735417 2020-06-26 South America / Brazil / São Paulo / Mongagua Human Original Nasopharyngeal swab Ion Torrent S5 IRMA B.1.1.28 GR
hCoV-19/Brazil/SP-766/2020 EPI_ISL_735418 2020-06-27 South America / Brazil / São Paulo / Taubate Human Original Nasopharyngeal swab Ion Torrent S5 IRMA B.1.1.94 GR
hCoV-19/Brazil/SP-770/2020 EPI_ISL_735419 2020-07-01 South America / Brazil / São Paulo / Juquia Human Original Nasopharyngeal swab Ion Torrent S5 IRMA B.1.1.28 GR
hCoV-19/Brazil/SP-772/2020 EPI_ISL_735420 2020-07-01 South America / Brazil / São Paulo / Sao Bernardo do Campo Human Original Nasopharyngeal swab Ion Torrent S5 IRMA B.1.1.29 GR
hCoV-19/Brazil/SP-773/2020 EPI_ISL_735421 2020-07-01 South America / Brazil / São Paulo / Sao Bernardo do Campo Human Original Nasopharyngeal swab Ion Torrent S5 IRMA B.1.1.28 GR
hCoV-19/Brazil/SP-774/2020 EPI_ISL_735422 2020-06-20 South America / Brazil / São Paulo / Sao Bernardo do Campo Human Original

Nasopharyngeal swab Ion Torrent S5 IRMA
B.1.1.28 GR
hCoV-19/Brazil/SP-783/2020 EPI_ISL_735423 2020-07-01 South America / Brazil / São Paulo / Diadema Human Original Nasopharyngeal swab
Ion Torrent S5 IRMA B.1.1.28 GR
hCoV-19/Brazil/SP-784/2020 EPI_ISL_735424 2020-07-01 South America / Brazil / São Paulo / Diadema Human Original Nasopharyngeal swab
Ion Torrent S5 IRMA B.1.1.28 GR
hCoV-19/Brazil/SP-785/2020 EPI_ISL_735425 2020-06-20 South America / Brazil / São Paulo / Ribeirao Pires Human Original Nasopharyngeal swab Ion Torrent S5 IRMA NS7a_Q94stop results in 23.1% truncation of the protein sequence. info B.1.1.33 GR
hCoV-19/Brazil/SP-786/2020 EPI_ISL_735426 2020-07-01 South America / Brazil / São Paulo / Diadema Human Original Nasopharyngeal swab
Ion Torrent S5 IRMA B.1.1.28 GR
hCoV-19/Brazil/SP-789/2020 EPI_ISL_735427 2020-07-01 South America / Brazil / São Paulo / Santos Human Original Nasopharyngeal swab
Ion Torrent S5 IRMA B.1.1.33 GR
hCoV-19/Brazil/SP-791/2020 EPI_ISL_735428 2020-07-02 South America / Brazil / São Paulo / São Paulo Human Original Nasopharyngeal swab
Ion Torrent S5 IRMA B.1.1.28 GR
hCoV-19/Brazil/SP-792/2020 EPI_ISL_735429 2020-07-01 South America / Brazil / São Paulo / São Paulo Human Original Nasopharyngeal swab
Ion Torrent S5 IRMA B.1.1.28 GR
hCoV-19/Brazil/SP-793/2020 EPI_ISL_735430 2020-07-01 South America / Brazil / São Paulo / Santos Human Original Nasopharyngeal swab
Ion Torrent S5 IRMA B.1.1.33 GR
hCoV-19/Brazil/SP-794/2020 EPI_ISL_735431 2020-07-02 South America / Brazil / São Paulo / São Paulo Human Original Nasopharyngeal swab
Ion Torrent S5 IRMA B.1.1.28 GR
hCoV-19/Brazil/SP-795/2020 EPI_ISL_735432 2020-07-02 South America / Brazil / São Paulo / São Paulo Human Original Nasopharyngeal swab
Ion Torrent S5 IRMA B.1.1.307 GR
hCoV-19/Brazil/SP-796/2020 EPI_ISL_735433 2020-07-01 South America / Brazil / São Paulo / Cajati Human Original Nasopharyngeal swab
Ion Torrent S5 IRMA B.1.1.28 GR
hCoV-19/Brazil/BA-1/2020 EPI_ISL_756293 2020-06-01 South America / Brazil / Bahia Human Original Reported case of reinfection (1st infection EPI_ISL_756293, 2nd infection EPI_ISL_756294) Ion Torrent
Genome Detective B.1.1.33 GR
hCoV-19/Brazil/SP-776/2020 EPI_ISL_776764 2020-07-01 South America / Brazil / São Paulo / Rio Grande da Serra Human Original
Nasopharyngeal swab Ion Torrent S5 IRMA
B.1.1.28 GR
hCoV-19/Brazil/SP-777/2020 EPI_ISL_776765 2020-07-01 South America / Brazil / São Paulo / Rio Grande da Serra Human Original
Nasopharyngeal swab Ion Torrent S5 IRMA
B.1.1.28 GR
hCoV-19/Brazil/SP-841/2020 EPI_ISL_792114 2020-05-03 South America / Brazil / Sao Paulo / Sao Paulo Human Original Nasopharyngeal swab
Ion Torrent S5 IRMA B.1.1.28 GR
hCoV-19/Brazil/SP-778/2020 EPI_ISL_792115 2020-06-29 South America / Brazil / Sao Paulo / Caraguatatuba Human Original Nasopharyngeal swab Ion Torrent S5 IRMA B.1.1.28 GR
hCoV-19/Brazil/SP-779/2020 EPI_ISL_792116 2020-07-02 South America / Brazil / Sao Paulo / Santa Isabel Human Original Nasopharyngeal swab
Ion Torrent S5 IRMA B.1.1.28 GR

hCoV-19/Brazil/PB-FIOCRUZ-23854-R1/2020 EPI_ISL_792561 2020-06-23 South America / Brazil / Paraiba / Joao Pessoa Human Original
Reported case of reinfection (1st infection EPI_ISL_792561, 2nd infection EPI_ISL_792562) Illumina Miseq CLC Genomics Workbench 12
B.1.1.33 GR
hCoV-19/Brazil/PB-FIOCRUZ-16521/2020 EPI_ISL_792563 2020-05-25 South America / Brazil / Paraiba / Cabedelo Human Original
Illumina Miseq CLC Genomics Workbench 12 B.1.1.29
GR
hCoV-19/Brazil/PB-FIOCRUZ-16522/2020 EPI_ISL_792564 2020-05-25 South America / Brazil / Paraiba / Santa Rita Human Original
Illumina Miseq CLC Genomics Workbench 12 B.1.1.29
GR
hCoV-19/Brazil/PB-FIOCRUZ-16523/2020 EPI_ISL_792565 2020-05-27 South America / Brazil / Paraiba / Joao Pessoa Human Original
Illumina Miseq CLC Genomics Workbench 12 B.1.1.29
GR
hCoV-19/Brazil/PB-FIOCRUZ-16524/2020 EPI_ISL_792566 2020-06-16 South America / Brazil / Paraiba / Joao Pessoa Human Original
Illumina Miseq CLC Genomics Workbench 12 B.1.1.29
GR
hCoV-19/Brazil/PB-FIOCRUZ-16525/2020 EPI_ISL_792567 2020-06-10 South America / Brazil / Paraiba / Itambe Human Original
Illumina Miseq CLC Genomics Workbench 12 B.1.1.29
GR
hCoV-19/Brazil/PB-FIOCRUZ-16526/2020 EPI_ISL_792568 2020-06-26 South America / Brazil / Paraiba / Joao Pessoa Human Original
Illumina Miseq CLC Genomics Workbench 12 B.1.1.29
GR
hCoV-19/Brazil/PB-FIOCRUZ-16527/2020 EPI_ISL_792569 2020-06-26 South America / Brazil / Paraiba / Joao Pessoa Human Original
Illumina Miseq CLC Genomics Workbench 12 B.1.1.29
GR
hCoV-19/Brazil/PB-FIOCRUZ-16606/2020 EPI_ISL_792593 2020-05-21 South America / Brazil / Paraiba / Joao Pessoa Human Original
Nanopore MinION CLC Genomics Workbench 12 B.1.1.33
GR
hCoV-19/Brazil/PB-FIOCRUZ-16610/2020 EPI_ISL_792594 2020-05-19 South America / Brazil / Paraiba / Joao Pessoa Human Original
Illumina Miseq CLC Genomics Workbench 12 B.1.1.33
GR
hCoV-19/Brazil/PB-FIOCRUZ-16615/2020 EPI_ISL_792595 2020-07-01 South America / Brazil / Paraiba / Pilar Human Original
Nanopore MinION CLC Genomics Workbench 12 Gap of 1 nucleotides when compared to the reference sequence. info B.1.1 GR
hCoV-19/Brazil/PB-FIOCRUZ-16616/2020 EPI_ISL_792596 2020-07-01 South America / Brazil / Paraiba / Joao Pessoa Human Original
Illumina Miseq CLC Genomics Workbench 12 B.1.1.33
GR
hCoV-19/Brazil/PB-FIOCRUZ-16617/2020 EPI_ISL_792597 2020-06-30 South America / Brazil / Paraiba / Cacimba de Dentro Human Original
Illumina Miseq CLC Genomics Workbench 12 B.1.1.29
GR
hCoV-19/Brazil/PB-FIOCRUZ-29604/2020 EPI_ISL_792602 2020-09-28 South America / Brazil / Paraiba Human Original Illumina Miseq
CLC Genomics Workbench 12 B.1.1.33 GR

hCoV-19/Brazil/PB-FIOCRUZ-29610/2020 EPI_ISL_792604 2020-08-06 South America / Brazil / Paraiba Human Original Illumina Miseq CLC Genomics Workbench 12 B.1.1.29 GR
hCoV-19/Brazil/PB-FIOCRUZ-29611/2020 EPI_ISL_792605 2020-08-10 South America / Brazil / Paraiba Human Original Illumina Miseq CLC Genomics Workbench 12 B.1.1.28 GR
hCoV-19/Brazil/PB-FIOCRUZ-29614/2020 EPI_ISL_792606 2020-08-11 South America / Brazil / Paraiba Human Original Illumina Miseq CLC Genomics Workbench 12 B.1.1.29 GR
hCoV-19/Brazil/PB-FIOCRUZ-29616/2020 EPI_ISL_792607 2020-08-12 South America / Brazil / Paraiba Human Original Illumina Miseq CLC Genomics Workbench 12 B.1.1.29 GR
hCoV-19/Brazil/PB-FIOCRUZ-29617/2020 EPI_ISL_792608 2020-08-19 South America / Brazil / Paraiba Human Original Illumina Miseq CLC Genomics Workbench 12 Gap of 9 nucleotides when compared to the reference sequence. info B.1.1.33 GR
hCoV-19/Brazil/PB-FIOCRUZ-29618/2020 EPI_ISL_792609 2020-08-20 South America / Brazil / Paraiba Human Original Illumina Miseq CLC Genomics Workbench 12 Gap of 3 nucleotides when compared to the reference sequence. info B.1.1.29 GR
hCoV-19/Brazil/PB-FIOCRUZ-29619/2020 EPI_ISL_792610 2020-08-20 South America / Brazil / Paraiba Human Original Illumina Miseq CLC Genomics Workbench 12 B.1.1.33 GR
hCoV-19/Brazil/PB-FIOCRUZ-29620/2020 EPI_ISL_792611 2020-08-31 South America / Brazil / Paraiba Human Original Illumina Miseq CLC Genomics Workbench 12 B.1.1.29 GR
hCoV-19/Brazil/PB-FIOCRUZ-29622/2020 EPI_ISL_792612 2020-09-14 South America / Brazil / Paraiba Human Original Illumina Miseq CLC Genomics Workbench 12 B.1.1.33 GR
hCoV-19/Brazil/PB-FIOCRUZ-29623/2020 EPI_ISL_792613 2020-09-16 South America / Brazil / Paraiba Human Original Illumina Miseq CLC Genomics Workbench 12 B.1.1.29 GR
hCoV-19/Brazil/PB-FIOCRUZ-29624/2020 EPI_ISL_792614 2020-09-21 South America / Brazil / Paraiba Human Original Illumina Miseq CLC Genomics Workbench 12 B.1.1.29 GR
hCoV-19/Brazil/PB-FIOCRUZ-29625/2020 EPI_ISL_792615 2020-09-21 South America / Brazil / Paraiba Human Original Illumina Miseq CLC Genomics Workbench 12 B.1.1.29 GR
hCoV-19/Brazil/PB-FIOCRUZ-29626/2020 EPI_ISL_792616 2020-09-23 South America / Brazil / Paraiba Human Original Illumina Miseq CLC Genomics Workbench 12 B.1.1.29 GR
hCoV-19/Brazil/PB-FIOCRUZ-29627/2020 EPI_ISL_792617 2020-09-24 South America / Brazil / Paraiba Human Original Illumina Miseq CLC Genomics Workbench 12 B.1.1.29 GR
hCoV-19/Brazil/PB-FIOCRUZ-29628/2020 EPI_ISL_792618 2020-09-24 South America / Brazil / Paraiba Human Original Illumina Miseq CLC Genomics Workbench 12 B.1.1.29 GR
hCoV-19/Brazil/PB-FIOCRUZ-29629/2020 EPI_ISL_792619 2020-09-24 South America / Brazil / Paraiba Human Original Illumina Miseq CLC Genomics Workbench 12 B.1.1.29 GR
hCoV-19/Brazil/PB-FIOCRUZ-29630/2020 EPI_ISL_792620 2020-09-30 South America / Brazil / Paraiba Human Original Illumina Miseq CLC Genomics Workbench 12 B.1.1.29 GR
hCoV-19/Brazil/PB-FIOCRUZ-29631/2020 EPI_ISL_792621 2020-09-30 South America / Brazil / Paraiba Human Original Illumina Miseq CLC Genomics Workbench 12 B.1.1.141 GR

hCoV-19/Brazil/AL-FIOCRUZ-28258/2020 EPI_ISL_792640 2020-05-06 South America / Brazil / Alagoas Human Original Illumina Miseq CLC Genomics Workbench 12 B.1.1.33 GR
hCoV-19/Brazil/AL-FIOCRUZ-30273/2020 EPI_ISL_792644 2020-05-13 South America / Brazil / Alagoas Human Original Illumina Miseq CLC Genomics Workbench 12 B.1.1.33 GR
hCoV-19/Brazil/AM-20141882LT/2020 EPI_ISL_801386 2020-09-24 South America / Brazil / Amazonas / Manaus Human Original Oropharyngeal swab Illumina MiSeq BBMap 37.25 embedded in Geneious 10.2.6 B.1.1.28 GR
hCoV-19/Brazil/AM-20141937RS/2020 EPI_ISL_801387 2020-09-12 South America / Brazil / Amazonas / Labrea Human Original Oropharyngeal swab Illumina MiSeq BBMap 37.25 embedded in Geneious 10.2.6 Gap of 3 nucleotides when compared to the reference sequence. info B.1.1.28 GR
hCoV-19/Brazil/AM-20141978DR/2020 EPI_ISL_801388 2020-09-12 South America / Brazil / Amazonas / Tapaua Human Original Oropharyngeal swab Illumina MiSeq BBMap 37.25 embedded in Geneious 10.2.6 Gap of 3 nucleotides when compared to the reference sequence. info B.1.1.28 GR
hCoV-19/Brazil/AM-20890012JJ/2020 EPI_ISL_801397 2020-08-24 South America / Brazil / Amazonas / Caapiranga Human Original Oropharyngeal swab Illumina MiSeq BBMap 37.25 embedded in Geneious 10.2.6 B.1.1.28 GR
hCoV-19/Brazil/AM-20890013MB/2020 EPI_ISL_801398 2020-08-24 South America / Brazil / Amazonas / Caapiranga Human Original Oropharyngeal swab Illumina MiSeq BBMap 37.25 embedded in Geneious 10.2.6 B.1.1.28 GR
hCoV-19/Brazil/AM-20890022AB/2020 EPI_ISL_801399 2020-06-09 South America / Brazil / Amazonas / Manaquiri Human Original Oropharyngeal swab Illumina MiSeq BBMap 37.25 embedded in Geneious 10.2.6 Gap of 1 nucleotide(s) found at refpos 5017 (FRAMESHIFT confirmed by submitter).info B.1.1.28 GR
hCoV-19/Brazil/AM-20890114ED/2020 EPI_ISL_801400 2020-09-14 South America / Brazil / Amazonas / Carauari Human Original Oropharyngeal swab Illumina MiSeq BBMap 37.25 embedded in Geneious 10.2.6 B.1.1.28 GR
hCoV-19/Brazil/AM-20890117JP/2020 EPI_ISL_801401 2020-09-15 South America / Brazil / Amazonas / Carauari Human Original Oropharyngeal swab Illumina MiSeq BBMap 37.25 embedded in Geneious 10.2.6 B.1.1.28 GR
hCoV-19/Brazil/AM-20890261MV/2020 EPI_ISL_801402 2020-05-03 South America / Brazil / Amazonas / Iranduba Human Original Oropharyngeal swab Illumina MiSeq BBMap 37.25 embedded in Geneious 10.2.6 B.1.1.28 GR
hCoV-19/Brazil/AM-20892948LS/2020 EPI_ISL_801403 2020-05-12 South America / Brazil / Amazonas / Manacapuru Human Original Oropharyngeal swab Illumina MiSeq BBMap 37.25 embedded in Geneious 10.2.6 B.1.1.28 GR
hCoV-19/Brazil/RS-11069/2020EPI_ISL_831474 2020-06-12 South America / Brazil / Rio Grande do Sul / Esteio Human Original Nasopharyngeal swab Illumina Miseq BWA, bcftools, bedtools, Nextclade v0.8.1, CoV-GLUE B.1.1.33 GR
hCoV-19/Brazil/RS-11262/2020EPI_ISL_831645 2020-06-15 South America / Brazil / Rio Grande do Sul / Esteio Human Original Nasopharyngeal swab Illumina Miseq BWA, bcftools, bedtools, Nextclade v0.8.1, CoV-GLUE B.1.1.28 GR

hCoV-19/Brazil/RS-11569/2020EPI_ISL_831646 2020-06-17 South America / Brazil / Rio Grande do Sul / Esteio Human Original
Illumina Miseq BWA, bcftools, bedtools, Nextclade v0.8.1, CoV-GLUE B.1.1.33 GR
hCoV-19/Brazil/RS-11574/2020EPI_ISL_831660 2020-06-17 South America / Brazil / Rio Grande do Sul / Esteio Human Original
Illumina Miseq BWA, bcftools, bedtools, Nextclade v0.8.1, CoV-GLUE B.1.1.28 GR
hCoV-19/Brazil/RS-9881/2020 EPI_ISL_831678 2020-05-31 South America / Brazil / Rio Grande do Sul / Esteio Human Original Nasopharyngeal swab Illumina Miseq BWA, bcftools, bedtools, Nextclade v0.8.1, CoV-GLUE B.1.1.33 GR
hCoV-19/Brazil/RS-13367/2020EPI_ISL_831681 2020-07-02 South America / Brazil / Rio Grande do Sul / Esteio Human Original Nasopharyngeal swab Illumina Miseq BWA, bcftools, bedtools, Nextclade v0.8.1, CoV-GLUE B.1.1.161 O
hCoV-19/Brazil/RS-13368/2020EPI_ISL_831683 2020-07-01 South America / Brazil / Rio Grande do Sul / Esteio Human Original Nasopharyngeal swab Illumina Miseq BWA, bcftools, bedtools, Nextclade v0.8.1, CoV-GLUE B.1.1.33 GR
hCoV-19/Brazil/RS-13979/2020EPI_ISL_831685 2020-07-06 South America / Brazil / Rio Grande do Sul / Esteio Human Original Nasopharyngeal swab Illumina Miseq BWA, bcftools, bedtools, Nextclade v0.8.1, CoV-GLUE B.1.1.33 GR
hCoV-19/Brazil/RS-15371/2020EPI_ISL_831688 2020-07-15 South America / Brazil / Rio Grande do Sul / Esteio Human Original Nasopharyngeal swab Illumina Miseq BWA, bcftools, bedtools, Nextclade v0.8.1, CoV-GLUE B.1.1.28 GR
hCoV-19/Brazil/RS-19337/2020EPI_ISL_831689 2020-08-03 South America / Brazil / Rio Grande do Sul / Esteio Human Original Nasopharyngeal swab Illumina Miseq BWA, bcftools, bedtools, Nextclade v0.8.1, CoV-GLUE B.1.1.28 GR
hCoV-19/Brazil/RS-22838/2020EPI_ISL_831892 2020-08-19 South America / Brazil / Rio Grande do Sul / Esteio Human Original Nasopharyngeal swab Illumina Miseq "BWA, bcftools, bedtools, Nextclade v0.8.1, CoV-GLUE " B.1.1.33 GR
hCoV-19/Brazil/RS-24277/2020EPI_ISL_831898 2020-08-26 South America / Brazil / Rio Grande do Sul / Esteio Human Original Nasopharyngeal swab Illumina Miseq BWA, bcftools, bedtools, Nextclade v0.8.1, CoV-GLUE B.1.1.33 GR
hCoV-19/Brazil/RS-24285/2020EPI_ISL_831913 2020-08-24 South America / Brazil / Rio Grande do Sul / Esteio Human Original Nasopharyngeal swab Illumina Miseq BWA, bcftools, bedtools, Nextclade v0.8.1, CoV-GLUE N.4 GR
hCoV-19/Brazil/RS-24565/2020EPI_ISL_831938 2020-08-27 South America / Brazil / Rio Grande do Sul / Esteio Human Original Nasopharyngeal swab Illumina Miseq BWA, bcftools, bedtools, Nextclade v0.8.1, CoV-GLUE B.1.1.28 GR
hCoV-19/Brazil/RS-25833/2020EPI_ISL_831939 2020-09-03 South America / Brazil / Rio Grande do Sul / Esteio Human Original Nasopharyngeal swab Illumina Miseq BWA, bcftools, bedtools, Nextclade v0.8.1, CoV-GLUE B.1.1.29 GR
hCoV-19/Brazil/RS-26977/2020EPI_ISL_831940 2020-09-10 South America / Brazil / Rio Grande do Sul / Esteio Human Original Nasopharyngeal swab Illumina Miseq BWA, bcftools, bedtools, Nextclade v0.8.1, CoV-GLUE B.1.1.33 GR
hCoV-19/Brazil/RS-27623/2020EPI_ISL_832009 2020-09-16 South America / Brazil / Rio Grande do Sul / Esteio Human Original Nasopharyngeal

swab	Illumina Miseq	BWA, bcftools, bedtools, Nextclade v0.8.1, CoV-GLUE		B.1.1.28	GR
hCoV-19/Brazil/SP-802/2020	EPI_ISL_833156	2020-07-02	South America / Brazil / Sao Paulo / Sorocaba	Human	Original	Nasopharyngeal swab	Ion Torrent S5	IRMA		B.1.1.28	GR
hCoV-19/Brazil/PA-IEC176228/2020	EPI_ISL_848608	2020-08-11	South America / Brazil / Para	Human	Original	Oropharyngeal swab	Ion Torrent S5	MEGAHIT v1.2.9 / Geneious Prime		P.2	GR
hCoV-19/Brazil/PA-IEC173156/2020	EPI_ISL_848609	2020-07-13	South America / Brazil / Para	Human	Original	Oropharyngeal swab	Illumina NextSeq	MEGAHIT v1.2.9 / Geneious Prime		B.1.1.33	GR
hCoV-19/Brazil/RR-IEC176364/2020	EPI_ISL_848610	2020-06-24	South America / Brazil / Roraima	Human	Original	Oropharyngeal swab	Ion Torrent S5	MEGAHIT v1.2.9 / Geneious Prime		B.1.1.33	GR
hCoV-19/Brazil/PA-IEC173145/2020	EPI_ISL_848611	2020-06-11	South America / Brazil / Para	Human	Original	Nasopharyngeal aspirate	Illumina NextSeq	MEGAHIT v1.2.9 / Geneious Prime		B.1.1.28	GR
hCoV-19/Brazil/AC-IEC169737/2020	EPI_ISL_848612	2020-06-10	South America / Brazil / Acre	Human	Original	Oropharyngeal swab	Illumina NextSeq	MEGAHIT v1.2.9 / Geneious Prime		B.1.1.33	GR
hCoV-19/Brazil/AC-IEC169749/2020	EPI_ISL_848613	2020-06-04	South America / Brazil / Acre	Human	Original	Oropharyngeal swab	Illumina NextSeq	MEGAHIT v1.2.9 / Geneious Prime		B.1.1.33	GR
hCoV-19/Brazil/PA-IEC173152/2020	EPI_ISL_848614	2020-06-01	South America / Brazil / Para	Human	Original	Oropharyngeal swab	Illumina NextSeq	MEGAHIT v1.2.9 / Geneious Prime		B.1.1.33	GR
hCoV-19/Brazil/PA-IEC173137/2020	EPI_ISL_848615	2020-05-05	South America / Brazil / Para	Human	Original	Oropharyngeal swab	Illumina NextSeq	MEGAHIT v1.2.9 / Geneious Prime		B.1.1.28	GR
hCoV-19/Brazil/SP-950/2020	EPI_ISL_861644	2020-05-05	South America / Brazil / Sao Paulo / Sao Paulo	Human	Original	Nasopharyngeal swab	Ion Torrent S9	IRMA		B.1.1.28	GR
hCoV-19/Brazil/SP-951/2020	EPI_ISL_861645	2020-05-07	South America / Brazil / Sao Paulo / Sao Paulo	Human	Original	Nasopharyngeal swab	Ion Torrent S10	IRMA		B.1.1.28	GR
hCoV-19/Brazil/SP-952/2020	EPI_ISL_861646	2020-05-21	South America / Brazil / Sao Paulo / Sao Paulo	Human	Original	Nasopharyngeal swab	Ion Torrent S11	IRMA		B.1.1.28	GR
hCoV-19/Brazil/SP-953/2020	EPI_ISL_861647	2020-05-25	South America / Brazil / Sao Paulo / Sao Paulo	Human	Original	Nasopharyngeal swab	Ion Torrent S12	IRMA		B.1.1.28	GR
hCoV-19/Brazil/SP-954/2020	EPI_ISL_861648	2020-05-28	South America / Brazil / Sao Paulo / Sao Paulo	Human	Original	Nasopharyngeal swab	Ion Torrent S13	IRMA		B.1.1.28	GR
hCoV-19/Brazil/SP-955/2020	EPI_ISL_861649	2020-05-28	South America / Brazil / Sao Paulo / Campinas	Human	Original	Nasopharyngeal swab	Ion Torrent S14	IRMA		B.1.1.28	GR
hCoV-19/Brazil/SP-956/2020	EPI_ISL_861650	2020-05-08	South America / Brazil / Sao Paulo / Sao Paulo	Human	Original	Nasopharyngeal swab

Ion Torrent S15 IRMA NS7a_E95stop results in 22.3% truncation of the protein sequence. info B.1.1.28 GR
hCoV-19/Brazil/SP-957/2020 EPI_ISL_861651 2020-05-10 South America / Brazil / Sao Paulo / Sao Paulo Human Original Nasopharyngeal swab Ion Torrent S16 IRMA B.1.1.33 GR
hCoV-19/Brazil/SP-958/2020 EPI_ISL_861652 2020-05-09 South America / Brazil / Sao Paulo / Sao Paulo Human Original Nasopharyngeal swab Ion Torrent S17 IRMA B.1.1.28 GR
hCoV-19/Brazil/SP-960/2020 EPI_ISL_861653 2020-05-13 South America / Brazil / Sao Paulo / Sao Paulo Human Original Nasopharyngeal swab Ion Torrent S18 IRMA B.1.1.33 GR
hCoV-19/Brazil/SP-961/2020 EPI_ISL_861654 2020-05-12 South America / Brazil / Sao Paulo / Sao Paulo Human Original Nasopharyngeal swab Ion Torrent S19 IRMA B.1.1.28 GR
hCoV-19/Brazil/SP-962/2020 EPI_ISL_861655 2020-05-12 South America / Brazil / Sao Paulo / Sao Paulo Human Original Nasopharyngeal swab Ion Torrent S20 IRMA B.1.1.28 GR
hCoV-19/Brazil/SP-963/2020 EPI_ISL_861656 2020-05-15 South America / Brazil / Sao Paulo / Jandira Human Original Nasopharyngeal swab Ion Torrent S21 IRMA B.1.1.28 GR
hCoV-19/Brazil/SP-964/2020 EPI_ISL_861657 2020-05-17 South America / Brazil / Sao Paulo / Osasco Human Original Nasopharyngeal swab Ion Torrent S22 IRMA B.1.1.28 GR
hCoV-19/Brazil/SP-965/2020 EPI_ISL_861658 2020-05-18 South America / Brazil / Sao Paulo / Osasco Human Original Nasopharyngeal swab Ion Torrent S23 IRMA B.1.1.28 GR
hCoV-19/Brazil/SP-966/2020 EPI_ISL_861659 2020-05-15 South America / Brazil / Sao Paulo / Barueri Human Original Nasopharyngeal swab Ion Torrent S24 IRMA B.1.1.296 GR
hCoV-19/Brazil/SP-967/2020 EPI_ISL_861660 2020-05-16 South America / Brazil / Sao Paulo / Barueri Human Original Nasopharyngeal swab Ion Torrent S25 IRMA B.1.1.28 GR
hCoV-19/Brazil/SP-968/2020 EPI_ISL_861661 2020-05-15 South America / Brazil / Sao Paulo / Barueri Human Original Nasopharyngeal swab Ion Torrent S26 IRMA B.1.1.28 GR
hCoV-19/Brazil/SP-969/2020 EPI_ISL_861662 2020-05-18 South America / Brazil / Sao Paulo / Presidente Venceslau Human Original Nasopharyngeal swab Ion Torrent S27 IRMA B.1.1.33 GR
hCoV-19/Brazil/SP-973/2020 EPI_ISL_861664 2020-05-27 South America / Brazil / Sao Paulo / Mogi-Guacu Human Original Nasopharyngeal swab Ion Torrent S29 IRMA B.1.1.33 GR
hCoV-19/Brazil/SP-781/2020 EPI_ISL_861665 2020-07-02 South America / Brazil / Sao Paulo / Sao Jose dos Campos Human Original Nasopharyngeal swab Ion Torrent S30 IRMA B.1.1.28 GR
hCoV-19/Brazil/SP-803/2020 EPI_ISL_861666 2020-05-30 South America / Brazil / Sao Paulo / Porto Feliz Human Original Nasopharyngeal swab Ion Torrent S31 IRMA B.1.1.28 GR
hCoV-19/Brazil/SP-806/2020 EPI_ISL_861667 2020-06-29 South America / Brazil / Sao Paulo / Rio Claro Human Original Nasopharyngeal swab Ion Torrent S32 IRMA B.1.1.28 GR
hCoV-19/Brazil/SP-HIAE-ID05/2020 EPI_ISL_861871 2020-08-01 South America / Brazil / São Paulo Human Original Illumina NextSeq B.1.1.33 GR
hCoV-19/Brazil/SP-HIAE-ID10/2020 EPI_ISL_861875 2020-07-16 South America / Brazil / São Paulo Human Original Illumina NextSeq B.1.1.28 O

hCoV-19/Brazil/SP-HIAE-ID19/2020 EPI_ISL_861884 2020-08-01 South America / Brazil / São PauloHuman Original Illumina NextSeq Gap of 3 nucleotides when compared to the reference sequence. info B.1.1.33 GR
hCoV-19/Brazil/SP-HIAE-ID20/2020 EPI_ISL_861885 2020-09-09 South America / Brazil / São PauloHuman Original Illumina NextSeq Gap of 12 nucleotides when compared to the reference sequence. info B.1.1.10 GR
hCoV-19/Brazil/SP-HIAE-ID26/2020 EPI_ISL_861891 2020-07-31 South America / Brazil / São PauloHuman Original Illumina NextSeq B.1.1.33 GR
hCoV-19/Brazil/SP-HIAE-ID31/2020 EPI_ISL_861895 2020-07-31 South America / Brazil / São PauloHuman Original Illumina NextSeq B.1.1.28 GR
hCoV-19/Brazil/SP-HIAE-ID33/2020 EPI_ISL_861897 2020-07-31 South America / Brazil / São PauloHuman Original Illumina NextSeq B.1.1.33 GR
hCoV-19/Brazil/SP-HIAE-ID37/2020 EPI_ISL_861901 2020-07-31 South America / Brazil / São PauloHuman Original Illumina NextSeq B.1.1.28 GR
hCoV-19/Brazil/SP-HIAE-ID46/2020 EPI_ISL_861910 2020-08-21 South America / Brazil / São PauloHuman Original Illumina NextSeq B.1.1.33 GR
hCoV-19/Brazil/SP-BT6815/2020 EPI_ISL_875540 2020-07-09 South America / Brazil / Sao Paulo / Sao Jose dos Campos Human Original Oropharyngeal swab Illumina MiSeq Geneious 10.2.4 B.1.1.28 GR
hCoV-19/Brazil/SP-BT6810/2020 EPI_ISL_875542 2020-08-09 South America / Brazil / Sao Paulo / Sao Jose dos Campos Human Original Oropharyngeal swab Illumina MiSeq Geneious 10.2.4 B.1.1.28 GR
hCoV-19/Brazil/SP-BT6798/2020 EPI_ISL_875543 2020-05-09 South America / Brazil / Sao Paulo / Sao Jose dos Campos Human Original Oropharyngeal swab Illumina MiSeq Geneious 10.2.4 Gap of 6 nucleotides when compared to the reference sequence. info B.1.1.28 GR
hCoV-19/Brazil/SP-BT6808/2020 EPI_ISL_875546 2020-08-09 South America / Brazil / Sao Paulo / Sao Jose dos Campos Human Original Oropharyngeal swab Illumina MiSeq Geneious 10.2.4 B.1.1.143 GR
hCoV-19/Brazil/SP-BT6816/2020 EPI_ISL_875547 2020-08-09 South America / Brazil / Sao Paulo / Sao Jose dos Campos Human Original Oropharyngeal swab Illumina MiSeq Geneious 10.2.4 B.1.1.28 GR
hCoV-19/Brazil/SP-BT6674/2020 EPI_ISL_875548 2020-05-09 South America / Brazil / Sao Paulo / Sao Jose dos Campos Human Original Oropharyngeal swab Illumina MiSeq Geneious 10.2.4 B.1.1.28 GR
hCoV-19/Brazil/SP-BT6799/2020 EPI_ISL_875549 2020-05-09 South America / Brazil / Sao Paulo / Sao Jose dos Campos Human Original Oropharyngeal swab Illumina MiSeq Geneious 10.2.4 B.1.1.28 GR
hCoV-19/Brazil/DF-1028R1/2020 EPI_ISL_882657 2020-07-23 South America / Brazil / Distrito Federal Human Original Nasopharyngeal swab Ion Torrent S5 IRMA P.2 GR
hCoV-19/Brazil/SP-BT6824/2020 EPI_ISL_888672 2020-07-09 South America / Brazil / Sao Paulo / Sao Jose dos Campos Human Original Oropharyngeal swab Illumina MiSeq Geneious 10.2.4 Gap

of 1 nucleotide(s) found at refpos 28254 (frameshift confirmed by the submitter).info B.1.1.28 GR
hCoV-19/Brazil/SP-864/2020 EPI_ISL_906067 2020-05-11 South America / Brazil / Sao Paulo / Barueri Human Original Nasopharyngeal swab Ion Torrent S5 IRMA B.1.1.28 GR
hCoV-19/Brazil/RR-IEC-176346/2020 EPI_ISL_918513 2020-06-01 South America / Brazil / Roraima Human Original Oropharyngeal swab Ion Torrent S5 MEGAHIT v1.2.9 / Geneious Prime B.1.1.33 GR
hCoV-19/Brazil/RS-32830/2020EPI_ISL_930855 2020-07-22 South America / Brazil / Rio Grande do Sul / Canoas Human Original Nanopore MinION B.1.1.33 GR
hCoV-19/Brazil/RS-33589/2020EPI_ISL_930856 2020-07-23 South America / Brazil / Rio Grande do Sul / Ibiruba Human Original Nanopore MinION B.1.1.28 GR
hCoV-19/Brazil/RS-33704/2020EPI_ISL_930857 2020-07-23 South America / Brazil / Rio Grande do Sul / Marau Human Original Nanopore MinION B.1.1.28 GR
hCoV-19/Brazil/RS-34046/2020EPI_ISL_930858 2020-07-24 South America / Brazil / Rio Grande do Sul / Canoas Human Original Nanopore MinION B.1.1.33 GR
hCoV-19/Brazil/SP-801/2020 EPI_ISL_940608 2020-06-26 South America / Brazil / Sao Paulo / Rio Claro Human Original Nasopharyngeal swab Ion Torrent S5 IRMA B.1.1.28 GR
hCoV-19/Brazil/RS-35834/2020EPI_ISL_942375 2020-07-31 South America / Brazil / Rio Grande do Sul / Cachoeira do Sul Human Original Nanopore MinION B.1.1.33 GR
hCoV-19/Brazil/RS-38171/2020EPI_ISL_942897 2020-08-06 South America / Brazil / Rio Grande do Sul / Teutonia Human Original Nanopore MinION B.1.1.33 GR
hCoV-19/Brazil/RS-38343/2020EPI_ISL_942898 2020-08-07 South America / Brazil / Rio Grande do Sul / Guaiba Human Original Nanopore MinION B.1.1.28 GR
hCoV-19/Brazil/RS-40591/2020EPI_ISL_942930 2020-08-13 South America / Brazil / Rio Grande do Sul / Viamao Human Original Nanopore MinION B.1.1.33 GR
hCoV-19/Brazil/RS-41964/2020EPI_ISL_942931 2020-08-14 South America / Brazil / Rio Grande do Sul / Lajeado Human Original Nanopore MinION B.1.1.33 GR
hCoV-19/Brazil/RS-42436/2020EPI_ISL_943574 2020-08-18 South America / Brazil / Rio Grande do Sul / Viamao Human Original Nanopore MinION B.1.1.33 GR
hCoV-19/Brazil/RS-42751/2020EPI_ISL_943575 2020-08-18 South America / Brazil / Rio Grande do Sul / Vera Cruz Human Original Nanopore MinION B.1.1.33 GR
hCoV-19/Brazil/RS-32833/2020EPI_ISL_943576 2020-07-16 South America / Brazil / Rio Grande do Sul / Tapes Human Original Nanopore MinION B.1.1.33 GR
hCoV-19/Brazil/RS-32831/2020EPI_ISL_943577 2020-07-22 South America / Brazil / Rio Grande do Sul / Canoas Human Original Nanopore MinION B.1.1.33 GR
hCoV-19/Brazil/RS-32082/2020EPI_ISL_943580 2020-07-16 South America / Brazil / Rio Grande do Sul / Santo Antonio da Patrulha Human Original Nanopore MinION B.1.91 G
hCoV-19/Brazil/RS-36765/2020EPI_ISL_943581 2020-08-03 South America / Brazil / Rio Grande do Sul / Rio Grande Human Original Nanopore MinION B.1.1.28 GR

hCoV-19/Brazil/RS-36776/2020EPI_ISL_943582 2020-08-04 South America / Brazil / Rio Grande do Sul / Torres Human Original
Nanopore MinION B.1.1.33 GR
hCoV-19/Brazil/RS-36850/2020EPI_ISL_943584 2020-08-04 South America / Brazil / Rio Grande do Sul / Rio Grande Human Original
Nanopore MinION B.1.1.28 GR
hCoV-19/Brazil/RS-39340/2020EPI_ISL_943586 2020-08-07 South America / Brazil / Rio Grande do Sul / Canoas Human Original
Nanopore MinION B.1.1.29 GR
hCoV-19/Brazil/RS-40476/2020EPI_ISL_943587 2020-08-12 South America / Brazil / Rio Grande do Sul / Vista Gaucha Human Original
Nanopore MinION B.1.1.29 GR
hCoV-19/Brazil/RS-38094/2020EPI_ISL_943588 2020-08-06 South America / Brazil / Rio Grande do Sul / Cachoeirnha Human Original
Nanopore MinION N.4 GR
hCoV-19/Brazil/RS-12423/2020EPI_ISL_943589 2020-05-18 South America / Brazil / Rio Grande do Sul / Passo Fundo Human Original
Nanopore MinION Gap of 6 nucleotides when compared to the reference sequence. info B.1.1.29 GR
hCoV-19/Brazil/RS-15237/2020EPI_ISL_943590 2020-06-02 South America / Brazil / Rio Grande do Sul / Tiradentes Human Original
Nanopore MinION B.1.1.33 GR
hCoV-19/Brazil/RS-16150/2020EPI_ISL_943591 2020-06-04 South America / Brazil / Rio Grande do Sul / Santo Angelo Human Original
Nanopore MinION B.1.1.33 GR
hCoV-19/Brazil/RS-18256/2020EPI_ISL_943593 2020-06-15 South America / Brazil / Rio Grande do Sul / Imbe Human Original Nanopore MinION B.1.1.33 GR
hCoV-19/Brazil/RS-11918/2020EPI_ISL_943595 2020-05-18 South America / Brazil / Rio Grande do Sul / Tramandai Human Original
Nanopore MinION N.4 GR
hCoV-19/Brazil/RS-12256/2020EPI_ISL_943596 2020-05-20 South America / Brazil / Rio Grande do Sul / Osorio Human Original
Nanopore MinION B.1.91 G
hCoV-19/Brazil/TO-1171/2020 EPI_ISL_943973 2020-09-24 South America / Brazil / Tocantins / Palmas Human Original Nasopharyngeal swab
Ion Torrent S5 IRMA B.1.1.29 GR
hCoV-19/Brazil/TO-1172/2020 EPI_ISL_943974 2020-09-25 South America / Brazil / Tocantins / Palmas Human Original Nasopharyngeal swab
Ion Torrent S5 IRMA B.1.1.28 GR
hCoV-19/Brazil/TO-1173/2020 EPI_ISL_943975 2020-09-25 South America / Brazil / Tocantins / Palmas Human Original Nasopharyngeal swab
Ion Torrent S5 IRMA B.1.1.28 GR
hCoV-19/Brazil/TO-1174/2020 EPI_ISL_943976 2020-09-29 South America / Brazil / Tocantins / Presidente Kennedy Human Original Nasopharyngeal swab Ion Torrent S5 IRMA B.1.1.28 GR
hCoV-19/Brazil/TO-1175/2020 EPI_ISL_943977 2020-09-29 South America / Brazil / Tocantins / Araguaiana Human Original Nasopharyngeal swab
Ion Torrent S5 IRMA B.1.1.28 GR
hCoV-19/Brazil/GO-1190R1/2020 EPI_ISL_943988 2020-06-24 South America / Brazil / Goias / Ceres Human Original Nasopharyngeal swab
Ion Torrent S5 IRMA B.1.1.28 GR
hCoV-19/Brazil/SP-1090/2020 EPI_ISL_977471 2020-08-10 South America / Brazil / Sao Paulo / Presidente Prudente Human Original
Nasopharyngeal swab Ion Torrent S5 IRMA
B.1.1.28 GR

hCoV-19/Brazil/SP-1091/2020 EPI_ISL_977472 2020-08-08 South America / Brazil / Sao Paulo / Sete Barras Human Original Nasopharyngeal swab Ion Torrent S5 IRMA B.1.1.28 GR
hCoV-19/Brazil/SP-1092/2020 EPI_ISL_977473 2020-08-14 South America / Brazil / Sao Paulo / Barueri Human Original Nasopharyngeal swab Ion Torrent S5 IRMA B.1.1.28 GR
hCoV-19/Brazil/SP-1093/2020 EPI_ISL_977474 2020-08-14 South America / Brazil / Sao Paulo / Sao Paulo Human Original Nasopharyngeal swab Ion Torrent S5 IRMA B.1.1.28 GR
hCoV-19/Brazil/SP-1094/2020 EPI_ISL_977475 2020-08-13 South America / Brazil / Sao Paulo / Presidente Prudente Human Original Nasopharyngeal swab Ion Torrent S5 IRMA B.1.1.28 GR
hCoV-19/Brazil/SP-1107/2020 EPI_ISL_977477 2020-09-29 South America / Brazil / Sao Paulo / Barueri Human Original Nasopharyngeal swab Ion Torrent S5 IRMA B.1.1.143 GR
hCoV-19/Brazil/SP-1109/2020 EPI_ISL_977478 2020-08-10 South America / Brazil / Sao Paulo / Presidente Prudente Human Original Nasopharyngeal swab Ion Torrent S5 IRMA B.1.1.28 GR
hCoV-19/Brazil/SP-1111/2020 EPI_ISL_977479 2020-08-09 South America / Brazil / Sao Paulo / Sao Paulo Human Original Nasopharyngeal swab Ion Torrent S5 IRMA B.1.1.33 GR
hCoV-19/Brazil/SP-1156/2020 EPI_ISL_977480 2020-08-18 South America / Brazil / Sao Paulo / Presidente Prudente Human Original Nasopharyngeal swab Ion Torrent S5 IRMA B.1.1.28 GR
hCoV-19/Brazil/SP-1158/2020 EPI_ISL_977481 2020-08-18 South America / Brazil / Sao Paulo / Ouro Verde Human Original Nasopharyngeal swab Ion Torrent S5 IRMA B.1.1.28 GR
hCoV-19/Brazil/SP-1162/2020 EPI_ISL_977483 2020-08-08 South America / Brazil / Sao Paulo / Sao Paulo Human Original Nasopharyngeal swab Ion Torrent S5 IRMA B.1.1.28 GR
hCoV-19/Brazil/SP-1163/2020 EPI_ISL_977484 2020-08-18 South America / Brazil / Sao Paulo / Santana de Parnaiba Human Original Nasopharyngeal swab Ion Torrent S5 IRMA B.1.1.28 GR
hCoV-19/Brazil/SP-1166/2020 EPI_ISL_977485 2020-08-13 South America / Brazil / Sao Paulo / Presidente Prudente Human Original Nasopharyngeal swab Ion Torrent S5 IRMA B.1.1.28 GR
hCoV-19/Brazil/SP-1167/2020 EPI_ISL_977486 2020-08-11 South America / Brazil / Sao Paulo / Diadema Human Original Nasopharyngeal swab Ion Torrent S5 IRMA B.1.1.33 GR
hCoV-19/Brazil/SP-1170/2020 EPI_ISL_977488 2020-08-13 South America / Brazil / Sao Paulo / Presidente Prudente Human Original Nasopharyngeal swab Ion Torrent S5 IRMA B.1.1.28 GR
hCoV-19/Brazil/BA-3/2020 EPI_ISL_978488 2020-06-28 South America / Brazil / Bahia Human Original Ion Torrent Genome Detective B.1.1.33 GR
hCoV-19/Brazil/BA-5/2020 EPI_ISL_978490 2020-05-21 South America / Brazil / Bahia Human Original Ion Torrent Genome Detective B.1.1.94 GR
hCoV-19/Brazil/BA-8/2020 EPI_ISL_978493 2020-05-30 South America / Brazil / Bahia Human Original Ion Torrent Genome Detective B.1.1.162 GR

hCoV-19/Brazil/BA-10/2020 EPI_ISL_978495 2020-08-19 South America / Brazil / Bahia Human Original Ion Torrent Genome Detective B.1.1.33 GR
hCoV-19/Brazil/BA-11/2020 EPI_ISL_978496 2020-06-28 South America / Brazil / Bahia Human Original Ion Torrent Genome Detective B.1 G
hCoV-19/Brazil/BA-12/2020 EPI_ISL_978497 2020-07-30 South America / Brazil / Bahia Human Original Ion Torrent Genome Detective B.1.1.33 GR
hCoV-19/Brazil/BA-13/2020 EPI_ISL_978498 2020-07-23 South America / Brazil / Bahia Human Original Ion Torrent Genome Detective B.1.1.28 GR
hCoV-19/Brazil/BA-14/2020 EPI_ISL_978499 2020-08-03 South America / Brazil / Bahia Human Original Ion Torrent Genome Detective B.1.1.33 GR
hCoV-19/Brazil/BA-15/2020 EPI_ISL_978500 2020-08-18 South America / Brazil / Bahia Human Original Ion Torrent Genome Detective N.4 GR
hCoV-19/Brazil/SP-1115/2020 EPI_ISL_984242 2020-08-13 South America / Brazil / Sao Paulo / Sao Paulo Human Original Nasopharyngeal swab Ion Torrent S5 IRMA B.1.1.28 GR
hCoV-19/Brazil/SP-1117/2020 EPI_ISL_984243 2020-08-21 South America / Brazil / Sao Paulo / Ourinhos Human Original Nasopharyngeal swab Ion Torrent S5 IRMA B.1.1.28 GR
hCoV-19/Brazil/SP-1118/2020 EPI_ISL_984244 2020-08-24 South America / Brazil / Sao Paulo / Taruma Human Original Nasopharyngeal swab Ion Torrent S5 IRMA B.1.1.28 GR
hCoV-19/Brazil/SP-1120/2020 EPI_ISL_984246 2020-08-14 South America / Brazil / Sao Paulo / Pariquera-Acu Human Original Nasopharyngeal swab Ion Torrent S5 IRMA B.1.1.28 GR
hCoV-19/Brazil/SP-1413R1/2020 EPI_ISL_984263 2020-09-29 South America / Brazil / Sao Paulo / Jau Human Original Nasopharyngeal swab Ion Torrent S7 IRMA B.1.1.28 GR
hCoV-19/Brazil/SP-1096/2020 EPI_ISL_985170 2020-08-28 South America / Brazil / Sao Paulo / Estrela do Norte Human Original Nasopharyngeal swab Ion Torrent S5 IRMA B.1.1.28 GR
hCoV-19/Brazil/SP-1103/2020 EPI_ISL_985171 2020-08-21 South America / Brazil / Sao Paulo / Sao Sebastiao Human Original Nasopharyngeal swab Ion Torrent S5 IRMA B.1.1.28 GR
hCoV-19/Brazil/SP-1097/2020 EPI_ISL_985172 2020-08-19 South America / Brazil / Sao Paulo / Lorena Human Original Nasopharyngeal swab Ion Torrent S5 IRMA B.1.1.28 GR
hCoV-19/Brazil/SP-1098/2020 EPI_ISL_985173 2020-08-19 South America / Brazil / Sao Paulo / Lorena Human Original Nasopharyngeal swab Ion Torrent S5 IRMA B.1.1.28 GR
hCoV-19/Brazil/SP-1099/2020 EPI_ISL_985174 2020-08-19 South America / Brazil / Sao Paulo / Cachoeira Paulista Human Original Nasopharyngeal swab Ion Torrent S5 IRMA B.1.1.33 GR
hCoV-19/Brazil/SP-1102/2020 EPI_ISL_985175 2020-08-17 South America / Brazil / Sao Paulo / Sao Paulo Human Original Nasopharyngeal swab Ion Torrent S5 IRMA B.1.1.28 GR
hCoV-19/Brazil/SP-1123/2020 EPI_ISL_985176 2020-08-17 South America / Brazil / Sao Paulo / Sao Paulo Human Original Nasopharyngeal swab Ion Torrent S5 IRMA B.1.1.33 GR
hCoV-19/Brazil/SP-1124/2020 EPI_ISL_985177 2020-08-17 South America / Brazil / Sao Paulo / Sao Paulo Human Original Nasopharyngeal swab Ion Torrent S5 IRMA B.1.1.143 GR

hCoV-19/Brazil/SP-1100/2020 EPI_ISL_985178 2020-08-15 South America / Brazil / Sao Paulo / Taboao da Serra Human Original Nasopharyngeal swab Ion Torrent S5 IRMA B.1.1.28 GR

Supplementary table 4: Acknowledgment to the authors from the laboratories responsible for obtaining the specimens, for submitting genetic sequence data and for sharing via the GISAID initiative, on which this research is based.

We gratefully acknowledge the following Authors from the Originating laboratories responsible for obtaining the specimens, as well as the Submitting laboratories where the genome data were generated and shared via GISAID, on which this research is based.

All Submitters of data may be contacted directly via www.gisaid.org

Authors are sorted alphabetically.

Accession ID	Originating Laboratory	Submitting Laboratory	Authors
EPI_ISL_1039696	Instituto Adolfo Lutz - Regional de Presidente Prudente	Instituto Adolfo Lutz, Interdiciplinary Procedures Center, Strategic Laboratory	Claudio Tavares Sacchi, Claudia Regina Gonçalves, Erica Valessa Ramos Gomes, Karoline Rodrigues Campos
EPI_ISL_1039697	Instituto Adolfo Lutz Central	Instituto Adolfo Lutz, Interdiciplinary Procedures Center, Strategic Laboratory	Claudio Tavares Sacchi, Claudia Regina Gonçalves, Erica Valessa Ramos Gomes, Karoline Rodrigues Campos
EPI_ISL_1039698	Lab Loc - Itapecerica da Serra	Instituto Adolfo Lutz, Interdiciplinary Procedures Center, Strategic Laboratory	Claudio Tavares Sacchi, Claudia Regina Gonçalves, Erica Valessa Ramos Gomes, Karoline Rodrigues Campos
EPI_ISL_1039699	Instituto Adolfo Lutz - Regional de Taubate	Instituto Adolfo Lutz, Interdiciplinary Procedures Center, Strategic Laboratory	Claudio Tavares Sacchi, Claudia Regina Gonçalves, Erica Valessa Ramos Gomes, Karoline Rodrigues Campos
EPI_ISL_1039700, EPI_ISL_1039701	Instituto Adolfo Lutz Central	Instituto Adolfo Lutz, Interdiciplinary Procedures Center, Strategic Laboratory	Claudio Tavares Sacchi, Claudia Regina Gonçalves, Erica Valessa Ramos Gomes, Karoline Rodrigues Campos
EPI_ISL_1039702	Instituto Adolfo Lutz - Regional de Aracatuba	Instituto Adolfo Lutz, Interdiciplinary Procedures Center, Strategic Laboratory	Claudio Tavares Sacchi, Claudia Regina Gonçalves, Erica Valessa Ramos Gomes, Karoline Rodrigues Campos
EPI_ISL_1040833, EPI_ISL_1040834, EPI_ISL_1040835, EPI_ISL_1040836, EPI_ISL_1040837, EPI_ISL_1040838, EPI_ISL_1040839, EPI_ISL_1040840, EPI_ISL_1040841, EPI_ISL_1040842, EPI_ISL_1040843, EPI_ISL_1040844, EPI_ISL_1040845, EPI_ISL_1040846, EPI_ISL_1040847, EPI_ISL_1040848, EPI_ISL_1040849, EPI_ISL_1040850, EPI_ISL_1040851			
see above	LACEN do Mato Grosso do Sul	Instituto Adolfo Lutz, Interdiciplinary Procedures Center, Strategic Laboratory	Claudio Tavares Sacchi, Claudia Regina Gonçalves, Erica Valessa Ramos Gomes, Karoline Rodrigues Campos
EPI_ISL_1061031	CDL Laboratorio Santos e Vidal LTDA.	Instituto de Medicina Tropical de Sao Paulo	Brazil-UK Centre for Arbovirus Discovery Diagnosis Genomics and Epidemiology (CADDE) Genomic Network - Instituto de Medicina Tropical
EPI_ISL_1063789	Evandro Chagas Institute	Evandro Chagas Institute Virology	Santos, M.C.; Silva, A.M.; Junior, W.D.C.; Barbagelata, L.S.; Ferreira, J.A.; Sousa, E.M.A.; da Silva, P.S.; Pinheiro, K.C.; L.C.; Sousa Junior, E.C.
EPI_ISL_1068089, EPI_ISL_1068090, EPI_ISL_1068091, EPI_ISL_1068092, EPI_ISL_1068093, EPI_ISL_1068094, EPI_ISL_1068095, EPI_ISL_1068096, EPI_ISL_1068097, EPI_ISL_1068098, EPI_ISL_1068099, EPI_ISL_1068100, EPI_ISL_1068120, EPI_ISL_1068121, EPI_ISL_1068122, EPI_ISL_1068168, EPI_ISL_1068170, EPI_ISL_1068171, EPI_ISL_1068172, EPI_ISL_1068173, EPI_ISL_1068174, EPI_ISL_1068175, EPI_ISL_1068177, EPI_ISL_1068180, EPI_ISL_1068182, EPI_ISL_1068187, EPI_ISL_1068188, EPI_ISL_1068189, EPI_ISL_1068190, EPI_ISL_1068197, EPI_ISL_1068201, EPI_ISL_1068203, EPI_ISL_1068204, EPI_ISL_1068205, EPI_ISL_1068206, EPI_ISL_1068207, EPI_ISL_1068208, EPI_ISL_1068209, EPI_ISL_1068211, EPI_ISL_1068212, EPI_ISL_1068213, EPI_ISL_1068214, EPI_ISL_1068216, EPI_ISL_1068217, EPI_ISL_1068218, EPI_ISL_1068224, EPI_ISL_1068227, EPI_ISL_1068228, EPI_ISL_1068229, EPI_ISL_1068230, EPI_ISL_1068231, EPI_ISL_1068232, EPI_ISL_1068233, EPI_ISL_1068234, EPI_ISL_1068235, EPI_ISL_1068236, EPI_ISL_1068237, EPI_ISL_1068238, EPI_ISL_1068239, EPI_ISL_1068240, EPI_ISL_1068241, EPI_ISL_1068242, EPI_ISL_1068244, EPI_ISL_1068245, EPI_ISL_1068246, EPI_ISL_1068247, EPI_ISL_1068250, EPI_ISL_1068251, EPI_ISL_1068252, EPI_ISL_1068253, EPI_ISL_1068254			
see above	Laboratorio de Ecologia de Doencas Transmissiveis na Amazonia, Instituto Leonidas e Maria Deane - Fiocruz Amazonia	Laboratorio de Ecologia de Doencas Transmissiveis na Amazonia, Instituto Leonidas e Maria Deane - Fiocruz Amazonia	Valdinete Nascimento, Victor Souza, André Corado, Fernanda Nascimento, George Silva, Ágatha Costa, Debora Duarte, Karina Pessoa, Matilde Mejía, Luciana Gonçalves, Maria Júlia Brandão, Michele Jesus, Felipe Naveca
EPI_ISL_1068317, EPI_ISL_1068319, EPI_ISL_1068322, EPI_ISL_1068324, EPI_ISL_1068325, EPI_ISL_1068326, EPI_ISL_1068327, EPI_ISL_1068328, EPI_ISL_1068329, EPI_ISL_1068363, EPI_ISL_1068364, EPI_ISL_1068366, EPI_ISL_1068367, EPI_ISL_1068369, EPI_ISL_1068370, EPI_ISL_1068371, EPI_ISL_1068372, EPI_ISL_1068373, EPI_ISL_1068374, EPI_ISL_1068375, EPI_ISL_1068376, EPI_ISL_1068377, EPI_ISL_1068378			
see above	Central Public Health Laboratory - LACEN -Bahia, Salvador, Brazil	Central Public Health Laboratory - LACEN -Bahia, Salvador, Brazil	Stephane Tosta, Luciana Oliveira, Vanessa Nardy,Patrícia Cajado,Marcela Gómez, Breno Dominguez, Jaqueline Gomes, Vagner Fonseca,Marta Giovanetti,Luiz Alcantara, Felicidade Pereira, Arabela Leal
EPI_ISL_1117379, EPI_ISL_1117380, EPI_ISL_1117381, EPI_ISL_1117382, EPI_ISL_1117383, EPI_ISL_1117384, EPI_ISL_1117385, EPI_ISL_1117386, EPI_ISL_1117387, EPI_ISL_1117388, EPI_ISL_1117389, EPI_ISL_1117390, EPI_ISL_1117391, EPI_ISL_1117392, EPI_ISL_1117393, EPI_ISL_1117394, EPI_ISL_1117395, EPI_ISL_1117396, EPI_ISL_1117397, EPI_ISL_1117398, EPI_ISL_1117399, EPI_ISL_1117400, EPI_ISL_1117401, EPI_ISL_1117402, EPI_ISL_1117403, EPI_ISL_1117404, EPI_ISL_1117405, EPI_ISL_1117406, EPI_ISL_1117407, EPI_ISL_1117408, EPI_ISL_1117409, EPI_ISL_1117410, EPI_ISL_1117411, EPI_ISL_1117412, EPI_ISL_1117413, EPI_ISL_1117414, EPI_ISL_1117415, EPI_ISL_1117416, EPI_ISL_1117417, EPI_ISL_1117418, EPI_ISL_1117419, EPI_ISL_1117420, EPI_ISL_1117421, EPI_ISL_1117422, EPI_ISL_1117423, EPI_ISL_1117424, EPI_ISL_1117425, EPI_ISL_1117426, EPI_ISL_1117427, EPI_ISL_1117428, EPI_ISL_1117429, EPI_ISL_1117430, EPI_ISL_1117431, EPI_ISL_1117432, EPI_ISL_1117433, EPI_ISL_1117434, EPI_ISL_1117435, EPI_ISL_1117436, EPI_ISL_1117437, EPI_ISL_1117438, EPI_ISL_1117439, EPI_ISL_1117440, EPI_ISL_1117441, EPI_ISL_1117442, EPI_ISL_1117443, EPI_ISL_1117444, EPI_ISL_1117445, EPI_ISL_1117446, EPI_ISL_1117447, EPI_ISL_1117448, EPI_ISL_1117449			
see above	Nucleo de Pesquisa em Inovacao Terapeutica - UFPE	LABBE, Federal University of Pernambuco	Wilson Jose da Silva Junior, Marcos da Silveira Regueira Neto, Heidi Lacerda Alves da Cruz, Bruno Sampaio, Reginaldo Goncalves de Lima Neto, Maira Galdino da Rocha Pitta, Michelly Cristiny Pereira, Marco Katzenberger, Valdir de Queiroz Balbino
EPI_ISL_1121326	IAL Regional de Bauru	Instituto Adolfo Lutz, Interdiciplinary Procedures Center, Strategic Laboratory	Claudio Tavares Sacchi, Claudia Regina Gonçalves, Erica Valessa Ramos Gomes, Karoline Rodrigues Campos, Caio Vinicius Dias Lopes
EPI_ISL_1121327, EPI_ISL_1121328, EPI_ISL_1121329, EPI_ISL_1121330, EPI_ISL_1139052, EPI_ISL_1139053, EPI_ISL_1139054, EPI_ISL_1139055, EPI_ISL_1139056, EPI_ISL_1139057	LACEN do Mato Grosso do Sul	Instituto Adolfo Lutz, Interdiciplinary Procedures Center, Strategic Laboratory	Claudio Tavares Sacchi, Claudia Regina Gonçalves, Erica Valessa Ramos Gomes, Karoline Rodrigues Campos, Caio Vinicius Dias Lopes
EPI_ISL_1163701, EPI_ISL_1163702, EPI_ISL_1163703, EPI_ISL_1163704, EPI_ISL_1163705, EPI_ISL_1163706, EPI_ISL_1163707, EPI_ISL_1163708, EPI_ISL_1163709, EPI_ISL_1163710, EPI_ISL_1163711			
see above	LABCOVID_HCPA	LABRESIS_HCPA	Martins AF, Wink PL, Volpato F, Rosset C, de Paris F, Monteiro F, Zavascki AP, Barth AL
EPI_ISL_1171620	Instituto Adolfo Lutz Central	Instituto Adolfo Lutz, Interdiciplinary Procedures Center, Strategic Laboratory	Claudio Tavares Sacchi, Claudia Regina Gonçalves, Erica Valessa Ramos Gomes, Karoline Rodrigues Campos, Caio Vinicius Dias Lopes
EPI_ISL_1182550	Fundação Ezequiel Dias (FUNED)	Coordenação Geral de Laboratórios de Saúde Pública (CGLAB/DAEVS/SVS/MS)	Vagner Fonseca, et al.
EPI_ISL_492035	Instituto de Biologia do Exército	Laboratório Metabolismo Macromolecular FirminoTorres de Castro, Instituto de Biofísica Carlos Chagas Filho, Universidade Federal do Rio de Janeiro	Bianca Catarina Azevedo Cabral, Aline Rosa Vianna de Souza, Tatiana LS Nogueira, Nádia Vaez Gonçalves da Cruz, Caleb GM Santos, Marcos Dornelas-Ribeiro, Elizabeth Valentin, Marcio da Costa Cipitelli, Virginia Sara Grancieri do Amaral, Rodrigo Soares de Moura Neto, Clarissa Damaso, Rosane Silva
EPI_ISL_492036	Instituto de Biologia do Exército	Laboratório Metabolismo Macromolecular FirminoTorres de Castro, Instituto de Biofísica Carlos Chagas Filho, Universidade Federal do Rio de Janeiro	Bianca Catarina Azevedo Cabral, Aline Rosa Vianna de Souza , Marcos Dornelas-Ribeiro, Tatiana LS Nogueira, Nádia Vaez Gonçalves da Cruz, Caleb GM Santos, Elizabeth Valentin, Marcio da Costa Cipitelli, Virginia Sara Grancieri do Amaral, Rodrigo Soares de Moura Neto, Clarissa Damaso, Rosane Silva
EPI_ISL_492043	Instituto de Biologia do Exército	Laboratório Metabolismo Macromolecular FirminoTorres de Castro, Instituto de Biofísica Carlos Chagas Filho, Universidade Federal do Rio de Janeiro	Bianca Catarina Azevedo Cabral, Aline Rosa Vianna de Souza, Tatiana LS Nogueira, Nádia Vaez Gonçalves da Cruz, Caleb GM Santos, Marcos Dornelas-Ribeiro, Elizabeth Valentin, Marcio da Costa Cipitelli, Virginia Sara Grancieri do Amaral, Rodrigo Soares de Moura Neto, Clarissa Damaso, Rosane Silva
EPI_ISL_492044	Instituto de Biologia do Exército	Laboratório Metabolismo Macromolecular FirminoTorres de Castro, Instituto de Biofísica Carlos Chagas Filho, Universidade Federal do Rio de Janeiro	Bianca Catarina Azevedo Cabral, Aline Rosa Vianna de Souza , Marcos Dornelas-Ribeiro, Tatiana LS Nogueira, Nádia Vaez Gonçalves da Cruz, Caleb GM Santos, Elizabeth Valentin, Marcio da Costa Cipitelli, Virginia Sara Grancieri do Amaral, Rodrigo Soares de Moura Neto, Clarissa Damaso, Rosane Silva

EPI_ISL_492045	Instituto de Biologia do Exército	Laboratório Metabolismo Macromolecular FirminoTorres de Castro, Instituto de Biofísica Carlos Chagas Filho, Universidade Federal do Rio de Janeiro	Bianca Catarina Azevedo Cabral, Aline Rosa Vianna de Souza, Caleb GM Santos, Marcos Dornelas-Ribeiro, Tatiana LS Nogueira, Nádia Vaez Gonçalves da Cruz, Elizabeth Valentin, Marcio da Costa Cipitelli, Virginia Sara Grancieri do Amaral, Rodrigo Soares de Moura Neto, Clarissa Damaso, Rosane Silva
EPI_ISL_492046	Instituto de Biologia do Exército	Laboratório Metabolismo Macromolecular FirminoTorres de Castro, Instituto de Biofísica Carlos Chagas Filho, Universidade Federal do Rio de Janeiro	Bianca Catarina Azevedo Cabral, Aline Rosa Vianna de Souza, Nádia Vaez Gonçalves da Cruz, Caleb GM Santos, Marcos Dornelas-Ribeiro, Tatiana LS Nogueira, Elizabeth Valentin, Marcio da Costa Cipitelli, Virginia Sara Grancieri do Amaral, Rodrigo Soares de Moura Neto, Clarissa Damaso, Rosane Silva
EPI_ISL_492047	Instituto de Biologia do Exército	Laboratório Metabolismo Macromolecular FirminoTorres de Castro, Instituto de Biofísica Carlos Chagas Filho, Universidade Federal do Rio de Janeiro	Bianca Catarina Azevedo Cabral, Aline Rosa Vianna de Souza, Tatiana LS Nogueira, Nádia Vaez Gonçalves da Cruz, Caleb GM Santos, Marcos Dornelas-Ribeiro, Elizabeth Valentin, Marcio da Costa Cipitelli, Virginia Sara Grancieri do Amaral, Rodrigo Soares de Moura Neto, Clarissa Damaso, Rosane Silva
EPI_ISL_492048	Instituto de Biologia do Exército	Laboratório Metabolismo Macromolecular FirminoTorres de Castro, Instituto de Biofísica Carlos Chagas Filho, Universidade Federal do Rio de Janeiro	Bianca Catarina Azevedo Cabral, Aline Rosa Vianna de Souza , Marcos Dornelas-Ribeiro, Tatiana LS Nogueira, Nádia Vaez Gonçalves da Cruz, Caleb GM Santos, Elizabeth Valentin, Marcio da Costa Cipitelli, Virginia Sara Grancieri do Amaral, Rodrigo Soares de Moura Neto, Clarissa Damaso, Rosane Silva
EPI_ISL_502779	LACEN/PE	LABBE, Federal University of Pernambuco	WILSON JOSE DA SILVA JUNIOR, HEIDI LACERDA ALVES DA CRUZ, MARCOS DA SILVEIRA REGUEIRA NETO, BRUNO SAMPAIO, SERGIO DE SA LEITAO PAIVA JUNIOR, ZILDENE DE SOUSA SILVEIRA, MAIRA GALDINO DA ROCHA PITTA, MICHELLY CRISTINY PEREIRA, MARCOS ANTONIO DE MORAIS JUNIOR, ANTONIO CARLOS DE FREITAS, VALDIR DE QUEIROZ BALBINO.
EPI_ISL_502875	LACEN/PE	LABBE, Federal University of Pernambuco	WILSON JOSE DA SILVA JUNIOR, HEIDI LACERDA ALVES DA CRUZ, MARCOS DA SILVEIRA REGUEIRA NETO, BRUNO SAMPAIO, SERGIO DE SA LEITAO PAIVA JUNIOR, ZILDENE DE SOUSA SILVEIRA, MAIRA GALDINO DA ROCHA PITTA, MICHELLY CRISTINY PEREIRA, REGINALDO GONCALVES DE LIMA NETO, MARCOS ANTONIO DE MORAIS JUNIOR, ANTONIO CARLOS DE FREITAS, VALDIR DE QUEIROZ BALBINO.
EPI_ISL_509430, EPI_ISL_509431, EPI_ISL_509432, EPI_ISL_509433, EPI_ISL_509435	Centro de Desenvolvimento Tecnologico em Saude, Fundacao Oswaldo Cruz	Centro de Desenvolvimento Tecnologico em Saude, Fundacao Oswaldo Cruz	Souza,T.M., Fintelman-Rodrigues,N., De Paula,A.D., Saraiva,F.B., Ferreira,M.A., Sacramento,C.Q., Medeiros,M.A.
EPI_ISL_510535	Molecular Virology, Instituto Carlos Chagas / Fiocruz Paraná	Universidade Federal do Parana (UFPR)	Suzukawa,A., Tscha,M., Zanluca,C., Raboni,S., Duarte dos Santos,C.
EPI_ISL_510536	Centro de Desenvolvimento Tecnologico em Saude, Fundacao Oswaldo Cruz	Centro de Desenvolvimento Tecnologico em Saude, Fundacao Oswaldo Cruz	Souza,T.M., Fintelman-Rodrigues,N., De Paula,A.D., Saraiva,F.B., Ferreira,M.A., Sacramento,C.Q. and Medeiros,M.A.
EPI_ISL_510541	Centro de Desenvolvimento Tecnologico em Saude, Fundacao Oswaldo Cruz	Centro de Desenvolvimento Tecnologico em Saude, Fundacao Oswaldo Cruz	Souza,T.M., Fintelman-Rodrigues,N., De Paula,A.D., Saraiva,F.B., Ferreira,M.A., Sacramento,C.Q. and Medeiros,M.A.
EPI_ISL_513573, EPI_ISL_513574, EPI_ISL_513575, EPI_ISL_513576, EPI_ISL_513577, EPI_ISL_513578, EPI_ISL_513579, EPI_ISL_513580, EPI_ISL_513581, EPI_ISL_513582, EPI_ISL_513583			
see above	Programa de Oncovirologia, Instituto Nacional de Câncer	Programa de Oncovirologia, Instituto Nacional de Câncer	Juliana D. Siqueira, Livia R. Goes, Brunna M. Alves, Claudia Cicala,James Arthos, João P.B. Viola, Andreia C. de Melo, Marcelo A. Soares
EPI_ISL_514131	Rondônia Central Public Health Laboratory (LACEN/RO), vinculated to State Health Secretariat of Rondônia (SESAU/RO)	Molecular Virology Laboratory of Oswaldo Cruz Foundation of Rondônia	Luan Felipo Botelho-Souza, Felipe Souza Nogueira-Lima, Tárcio Peixoto Roca, Alcione de Oliveira dos Santos, Felipe Gomes Naveca, Adriana Cristina Salvador Maia, Cicileia Correia da Silva, Aline Linhares Ferreira de Melo Mendonça, Celina Aparecida Bertoni Lugtenburg, Camila Flávia Gomes Azzi, Juliana Loca Furtado, Suelen Cavalcante, Rita de Cássia Pontello Rampazzo, Caio Henrique Nemeth Santos, Alice Paula Di Sabatino Guimarães, Jansen Fernandes de Medeiros, Fernando Rodrigues Máximo, Juan Miguel Vilallobos-Salcedo and Deusilene Souza Vieira1
EPI_ISL_514132	Rondônia Central Public Health Laboratory (LACEN/RO), vinculated to State Health Secretariat of Rondônia (SESAU/RO)	Molecular Virology Laboratory of Oswaldo Cruz Foundation of Rondônia	Luan Felipo Botelho-Souza, Felipe Souza Nogueira-Lima, Tárcio Peixoto Roca, Alcione de Oliveira dos Santos, Felipe Gomes Naveca, Adriana Cristina Salvador Maia, Cicileia Correia da Silva, Aline Linhares Ferreira de Melo Mendonça, Celina Aparecida Bertoni Lugtenburg, Camila Flávia Gomes Azzi, Juliana Loca Furtado, Suelen Cavalcante, Rita de Cássia Pontello Rampazzo, Caio Henrique Nemeth Santos, Alice Paula Di Sabatino Guimarães, Jansen Fernandes de Medeiros, Fernando Rodrigues Máximo, Juan Miguel Vilallobos-Salcedo and Deusilene Souza Vieira.
EPI_ISL_514133, EPI_ISL_514134, EPI_ISL_514135, EPI_ISL_514136, EPI_ISL_514137, EPI_ISL_514138	Rondônia Central Public Health Laboratory (LACEN/RO), vinculated to State Health Secretariat of Rondônia (SESAU/RO)	Molecular Virology Laboratory of Oswaldo Cruz Foundation of Rondônia	Luan Felipo Botelho-Souza, Felipe Souza Nogueira-Lima, Tárcio Peixoto Roca, Alcione de Oliveira dos Santos, Felipe Gomes Naveca, Adriana Cristina Salvador Maia, Cicileia Correia da Silva, Aline Linhares Ferreira de Melo Mendonça, Celina Aparecida Bertoni Lugtenburg, Camila Flávia Gomes Azzi, Juliana Loca Furtado, Suelen Cavalcante, Rita de Cássia Pontello Rampazzo, Caio Henrique Nemeth Santos, Alice Paula Di Sabatino Guimarães, Jansen Fernandes de Medeiros, Fernando Rodrigues Máximo, Juan Miguel Vilallobos-Salcedo and Deusilene Souza Vieira
EPI_ISL_515525	National Influenza Center - Instituto Adolfo Lutz	Instituto Adolfo Lutz, Interdiciplinary Procedures Center, Strategic Laboratory	Claudio Tavares Sacchi, Claudia Regina Gonçalves, Erica Valessa Ramos Gomes
EPI_ISL_523959	Pronto Socorro Municipal de Perus	Instituto Adolfo Lutz, Interdiciplinary Procedures Center, Strategic Laboratory	Claudio Tavares Sacchi, Claudia Regina Gonçalves, Erica Valessa Ramos Gomes
EPI_ISL_524468	Hospital Municipal Vereador Jose Storopolli	Instituto Adolfo Lutz, Interdiciplinary Procedures Center, Strategic Laboratory	Claudio Tavares Sacchi, Claudia Regina Gonçalves, Erica Valessa Ramos Gomes
EPI_ISL_524469	Santa Casa de Misericordia de Sao Paulo	Instituto Adolfo Lutz, Interdiciplinary Procedures Center, Strategic Laboratory	Claudio Tavares Sacchi, Claudia Regina Gonçalves, Erica Valessa Ramos Gomes
EPI_ISL_524470	Hospital do Servidor Público Estadual Francisco Morato de Oliveira	Instituto Adolfo Lutz, Interdiciplinary Procedures Center, Strategic Laboratory	Claudio Tavares Sacchi, Claudia Regina Gonçalves, Erica Valessa Ramos Gomes
EPI_ISL_529139	Centro de Desenvolvimento Tecnologico em Saude, Fundacao Oswaldo Cruz	Centro de Desenvolvimento Tecnologico em Saude, Fundacao Oswaldo Cruz	Souza,T.M., Fintelman-Rodrigues,N., De Paula,A.D., Saraiva,F.B., Ferreira,M.A., Sacramento,C.Q., Medeiros,M.A.
EPI_ISL_529140	Centro de Desenvolvimento Tecnologico em Saude, Fundacao Oswaldo Cruz	Centro de Desenvolvimento Tecnologico em Saude, Fundacao Oswaldo Cruz	Souza,T.M., Fintelman-Rodrigues,N., De Paula,A.D., Saraiva,F.B., Ferreira,M.A., Sacramento,C.Q., Medeiros,M.A.
EPI_ISL_534312	Distrito Sanitario Sul	Instituto Adolfo Lutz, Interdiciplinary Procedures Center, Strategic Laboratory	Claudio Tavares Sacchi, Claudia Regina Gonçalves, Erica Valessa Ramos Gomes
EPI_ISL_534314	Hospital Universitario da USP de SP	Instituto Adolfo Lutz, Interdiciplinary Procedures Center, Strategic Laboratory	Claudio Tavares Sacchi, Claudia Regina Gonçalves, Erica Valessa Ramos Gomes
EPI_ISL_534315	Serviço de Verificação de Óbitos SVO Guaruulhos	Instituto Adolfo Lutz, Interdiciplinary Procedures Center, Strategic Laboratory	Claudio Tavares Sacchi, Claudia Regina Gonçalves, Erica Valessa Ramos Gomes
EPI_ISL_534316	OS Mun Santana Lauro Ribas Braga	Instituto Adolfo Lutz, Interdiciplinary Procedures Center, Strategic Laboratory	Claudio Tavares Sacchi, Claudia Regina Gonçalves, Erica Valessa Ramos Gomes
EPI_ISL_534317	Hospital Geral de Itapevi	Instituto Adolfo Lutz, Interdiciplinary Procedures Center, Strategic Laboratory	Claudio Tavares Sacchi, Claudia Regina Gonçalves, Erica Valessa Ramos Gomes
EPI_ISL_534318	Hospital Municipal Antonio Giglio	Instituto Adolfo Lutz, Interdiciplinary Procedures Center, Strategic Laboratory	Claudio Tavares Sacchi, Claudia Regina Gonçalves, Erica Valessa Ramos Gomes
EPI_ISL_534319, EPI_ISL_534320	Hospital do Serv Pub ESTAFCO Morato de Oliveira	Instituto Adolfo Lutz, Interdiciplinary Procedures Center, Strategic Laboratory	Claudio Tavares Sacchi, Claudia Regina Gonçalves, Erica Valessa Ramos Gomes
EPI_ISL_534321	PS e Maternidade Nair Fonseca Leitao Arantes	Instituto Adolfo Lutz, Interdiciplinary Procedures Center, Strategic Laboratory	Claudio Tavares Sacchi, Claudia Regina Gonçalves, Erica Valessa Ramos Gomes
EPI_ISL_534326	Notre Dame Intermedica Saude AS	Instituto Adolfo Lutz, Interdiciplinary Procedures Center, Strategic Laboratory	Claudio Tavares Sacchi, Claudia Regina Gonçalves, Erica Valessa Ramos Gomes

EPI_ISL_547571	Hospital Municipal Antônio Giglio	Instituto Adolfo Lutz, Interdiciplinary Procedures Center, Strategic Laboratory	Claudio Tavares Sacchi, Claudia Regina Gonçalves, Erica Valessa Ramos Gomes, Karoline Rodrigues Campos
EPI_ISL_547573	Vigilância em Saúde de Cajamar	Instituto Adolfo Lutz, Interdiciplinary Procedures Center, Strategic Laboratory	Claudio Tavares Sacchi, Claudia Regina Gonçalves, Erica Valessa Ramos Gomes, Karoline Rodrigues Campos
EPI_ISL_547574	Hospital Universitario da USP	Instituto Adolfo Lutz, Interdiciplinary Procedures Center, Strategic Laboratory	Claudio Tavares Sacchi, Claudia Regina Gonçalves, Erica Valessa Ramos Gomes, Karoline Rodrigues Campos
EPI_ISL_547575	SVO Jundiaí	Instituto Adolfo Lutz, Interdiciplinary Procedures Center, Strategic Laboratory	Claudio Tavares Sacchi, Claudia Regina Gonçalves, Erica Valessa Ramos Gomes, Karoline Rodrigues Campos
EPI_ISL_547576	Secretaria Municipal de Saúde	Instituto Adolfo Lutz, Interdiciplinary Procedures Center, Strategic Laboratory	Claudio Tavares Sacchi, Claudia Regina Gonçalves, Erica Valessa Ramos Gomes, Karoline Rodrigues Campos
EPI_ISL_547577	Hospital e Maternidade Nossa Senhora das Graças	Instituto Adolfo Lutz, Interdiciplinary Procedures Center, Strategic Laboratory	Claudio Tavares Sacchi, Claudia Regina Gonçalves, Erica Valessa Ramos Gomes, Karoline Rodrigues Campos
EPI_ISL_547578	Hospital Doutor Domingos Leonardo Cerávolo	Instituto Adolfo Lutz, Interdiciplinary Procedures Center, Strategic Laboratory	Claudio Tavares Sacchi, Claudia Regina Gonçalves, Erica Valessa Ramos Gomes, Karoline Rodrigues Campos
EPI_ISL_547579	Santa Casa de Misericórdia de Araçatuba	Instituto Adolfo Lutz, Interdiciplinary Procedures Center, Strategic Laboratory	Claudio Tavares Sacchi, Claudia Regina Gonçalves, Erica Valessa Ramos Gomes, Karoline Rodrigues Campos
EPI_ISL_547580	Santa Casa da Misericórdia de Presidente Prudente	Instituto Adolfo Lutz, Interdiciplinary Procedures Center, Strategic Laboratory	Claudio Tavares Sacchi, Claudia Regina Gonçalves, Erica Valessa Ramos Gomes, Karoline Rodrigues Campos
EPI_ISL_572366, EPI_ISL_572371, EPI_ISL_572386	LACEN/PE	WallauLab, Aggeu Magalhaes Institute	Marcelo Henrique Santos Paiva, Duschinka Ribeiro Duarte Guedes, Cássia Docena, Matheus Filgueira Bezerra, Filipe Zimmer Dezordi, Laís Ceschini Machado, Larissa Krokovsky, Elisama Helvecio, Alexandre Freitas da Silva, Luydson Richardson Silva Vasconcelos, Antonio Mauro Rezende, Severino Jefferson Ribeiro da Silva, Kamila Gaudêncio da Silva Sales, Bruna Santos Lima Figueiredo de Sá, Derciliano Lopes da Cruz, Claudio Eduardo Cavalcanti, Armando de Menezes Neto, Caroline Targino Alves da Silva, Renata Pessôa Germano Mendes, Maria Almerice Lopes da Silva, Tiago Gräf, Paola Cristina Resende, Gonzalo Bello0, Michelle da Silva Barros, Wheverton Ricardo Correia do Nascimento,, Rodrigo Moraes Loyo Arcoverde, Luciane Caroline Albuquerque Bezerra, Sinval Pinto Brandão Filho, Constância Flávia Junqueira Ayres, Gabriel Luz Wallau
EPI_ISL_574594	Hospital Escola da Universidade de Taubate	Instituto Adolfo Lutz, Interdiciplinary Procedures Center, Strategic Laboratory	Claudio Tavares Sacchi, Claudia Regina Gonçalves, Erica Valessa Ramos Gomes, Karoline Rodrigues Campos
EPI_ISL_574596	CS II Dr. Antonio Vicoso Moreira de Rezende Sumare	Instituto Adolfo Lutz, Interdiciplinary Procedures Center, Strategic Laboratory	Claudio Tavares Sacchi, Claudia Regina Gonçalves, Erica Valessa Ramos Gomes, Karoline Rodrigues Campos
EPI_ISL_574597	Secretaria Municipal de Saude de Jarinu	Instituto Adolfo Lutz, Interdiciplinary Procedures Center, Strategic Laboratory	Claudio Tavares Sacchi, Claudia Regina Gonçalves, Erica Valessa Ramos Gomes, Karoline Rodrigues Campos
EPI_ISL_574598	Servico de Verificacao de Obito SVO	Instituto Adolfo Lutz, Interdiciplinary Procedures Center, Strategic Laboratory	Claudio Tavares Sacchi, Claudia Regina Gonçalves, Erica Valessa Ramos Gomes, Karoline Rodrigues Campos
EPI_ISL_583495	Serviço de Verificação de Óbitos SVO Guaruulhos	Instituto Adolfo Lutz, Interdiciplinary Procedures Center, Strategic Laboratory	Claudio Tavares Sacchi, Claudia Regina Gonçalves, Erica Valessa Ramos Gomes, Karoline Rodrigues Campos
EPI_ISL_583496	UPA Jandira	Instituto Adolfo Lutz, Interdiciplinary Procedures Center, Strategic Laboratory	Claudio Tavares Sacchi, Claudia Regina Gonçalves, Erica Valessa Ramos Gomes, Karoline Rodrigues Campos
EPI_ISL_583497	Complexo Hospitalar Ouro Verde de Campinas	Instituto Adolfo Lutz, Interdiciplinary Procedures Center, Strategic Laboratory	Claudio Tavares Sacchi, Claudia Regina Gonçalves, Erica Valessa Ramos Gomes, Karoline Rodrigues Campos
EPI_ISL_583498	Hospital Municipal Dr. Waldemar Tebaldi	Instituto Adolfo Lutz, Interdiciplinary Procedures Center, Strategic Laboratory	Claudio Tavares Sacchi, Claudia Regina Gonçalves, Erica Valessa Ramos Gomes, Karoline Rodrigues Campos
EPI_ISL_583499	Distrito Sanitario Sul Campinas	Instituto Adolfo Lutz, Interdiciplinary Procedures Center, Strategic Laboratory	Claudio Tavares Sacchi, Claudia Regina Gonçalves, Erica Valessa Ramos Gomes, Karoline Rodrigues Campos
EPI_ISL_583500	Centro de Saude I Tacito Leite de Carvalho e Silva	Instituto Adolfo Lutz, Interdiciplinary Procedures Center, Strategic Laboratory	Claudio Tavares Sacchi, Claudia Regina Gonçalves, Erica Valessa Ramos Gomes, Karoline Rodrigues Campos
EPI_ISL_583501	Hospital Estadual de CampanhaCOVID 19 Barradas	Instituto Adolfo Lutz, Interdiciplinary Procedures Center, Strategic Laboratory	Claudio Tavares Sacchi, Claudia Regina Gonçalves, Erica Valessa Ramos Gomes, Karoline Rodrigues Campos
EPI_ISL_583502	Serv de Vig Sanitaria Epidemio e CTRL de Zoonoses Guaruja	Instituto Adolfo Lutz, Interdiciplinary Procedures Center, Strategic Laboratory	Claudio Tavares Sacchi, Claudia Regina Gonçalves, Erica Valessa Ramos Gomes, Karoline Rodrigues Campos
EPI_ISL_583503	CTA Centro de Testagem e Aconselhamento	Instituto Adolfo Lutz, Interdiciplinary Procedures Center, Strategic Laboratory	Claudio Tavares Sacchi, Claudia Regina Gonçalves, Erica Valessa Ramos Gomes, Karoline Rodrigues Campos
EPI_ISL_583504, EPI_ISL_583505	Casa de Saude Stella Maris	Instituto Adolfo Lutz, Interdiciplinary Procedures Center, Strategic Laboratory	Claudio Tavares Sacchi, Claudia Regina Gonçalves, Erica Valessa Ramos Gomes, Karoline Rodrigues Campos
EPI_ISL_603021	Pronto Socorro Dr. Conrado Cesarino Nuvolini	Instituto Adolfo Lutz, Interdiciplinary Procedures Center, Strategic Laboratory	Claudio Tavares Sacchi, Claudia Regina Gonçalves, Erica Valessa Ramos Gomes, Karoline Rodrigues Campos
EPI_ISL_603022	Departamento de Vigilância à Saúde	Instituto Adolfo Lutz, Interdiciplinary Procedures Center, Strategic Laboratory	Claudio Tavares Sacchi, Claudia Regina Gonçalves, Erica Valessa Ramos Gomes, Karoline Rodrigues Campos
EPI_ISL_603023	Vigilância em Saúde Visa Sul	Instituto Adolfo Lutz, Interdiciplinary Procedures Center, Strategic Laboratory	Claudio Tavares Sacchi, Claudia Regina Gonçalves, Erica Valessa Ramos Gomes, Karoline Rodrigues Campos
EPI_ISL_603024	Santa Casa de Misericordia de Araçatuba	Instituto Adolfo Lutz, Interdiciplinary Procedures Center, Strategic Laboratory	Claudio Tavares Sacchi, Claudia Regina Gonçalves, Erica Valessa Ramos Gomes, Karoline Rodrigues Campos
EPI_ISL_603025	UPA Central de Caraguatatuba	Instituto Adolfo Lutz, Interdiciplinary Procedures Center, Strategic Laboratory	Claudio Tavares Sacchi, Claudia Regina Gonçalves, Erica Valessa Ramos Gomes, Karoline Rodrigues Campos
EPI_ISL_603026	Santa Casa da Misericórdia de Presidente Prudente	Instituto Adolfo Lutz, Interdiciplinary Procedures Center, Strategic Laboratory	Claudio Tavares Sacchi, Claudia Regina Gonçalves, Erica Valessa Ramos Gomes, Karoline Rodrigues Campos
EPI_ISL_603027	Santa Casa de Misericordia de Araçatuba	Instituto Adolfo Lutz, Interdiciplinary Procedures Center, Strategic Laboratory	Claudio Tavares Sacchi, Claudia Regina Gonçalves, Erica Valessa Ramos Gomes, Karoline Rodrigues Campos
EPI_ISL_603028	Hospital Municipal Santa Ana	Instituto Adolfo Lutz, Interdiciplinary Procedures Center, Strategic Laboratory	Claudio Tavares Sacchi, Claudia Regina Gonçalves, Erica Valessa Ramos Gomes, Karoline Rodrigues Campos
EPI_ISL_603029	Hospital Municipal Mário Gatti	Instituto Adolfo Lutz, Interdiciplinary Procedures Center, Strategic Laboratory	Claudio Tavares Sacchi, Claudia Regina Gonçalves, Erica Valessa Ramos Gomes, Karoline Rodrigues Campos
EPI_ISL_603030	Hospital Domingos Leonardo Ceravolo Presidente Prudente	Instituto Adolfo Lutz, Interdiciplinary Procedures Center,	Claudio Tavares Sacchi, Claudia Regina Gonçalves, Erica Valessa Ramos Gomes, Karoline Rodrigues Campos

		Strategic Laboratory	
EPI_ISL_603031	Santa Casa de Presidente Epitácio	Instituto Adolfo Lutz, Interdiciplinary Procedures Center, Strategic Laboratory	Claudio Tavares Sacchi, Claudia Regina Gonçalves, Erica Valessa Ramos Gomes, Karoline Rodrigues Campos
EPI_ISL_603032	Santa Casa da Misericórdia de Presidente Prudente	Instituto Adolfo Lutz, Interdiciplinary Procedures Center, Strategic Laboratory	Claudio Tavares Sacchi, Claudia Regina Gonçalves, Erica Valessa Ramos Gomes, Karoline Rodrigues Campos
EPI_ISL_603033	Vigilancia Epidemiologica de São Bernardo do Campo	Instituto Adolfo Lutz, Interdiciplinary Procedures Center, Strategic Laboratory	Claudio Tavares Sacchi, Claudia Regina Gonçalves, Erica Valessa Ramos Gomes, Karoline Rodrigues Campos
EPI_ISL_603034	Departamento de Vigilância à Saúde	Instituto Adolfo Lutz, Interdiciplinary Procedures Center, Strategic Laboratory	Claudio Tavares Sacchi, Claudia Regina Gonçalves, Erica Valessa Ramos Gomes, Karoline Rodrigues Campos
EPI_ISL_603035	Secretaria Municipal de Saúde	Instituto Adolfo Lutz, Interdiciplinary Procedures Center, Strategic Laboratory	Claudio Tavares Sacchi, Claudia Regina Gonçalves, Erica Valessa Ramos Gomes, Karoline Rodrigues Campos
EPI_ISL_603036	Hospital Santa Ana	Instituto Adolfo Lutz, Interdiciplinary Procedures Center, Strategic Laboratory	Claudio Tavares Sacchi, Claudia Regina Gonçalves, Erica Valessa Ramos Gomes, Karoline Rodrigues Campos
EPI_ISL_603037	Hospital Geral de Pedreira	Instituto Adolfo Lutz, Interdiciplinary Procedures Center, Strategic Laboratory	Claudio Tavares Sacchi, Claudia Regina Gonçalves, Erica Valessa Ramos Gomes, Karoline Rodrigues Campos
EPI_ISL_603038	Santa Casa de Misericordia de Araçatuba	Instituto Adolfo Lutz, Interdiciplinary Procedures Center, Strategic Laboratory	Claudio Tavares Sacchi, Claudia Regina Gonçalves, Erica Valessa Ramos Gomes, Karoline Rodrigues Campos
EPI_ISL_603039	Hospital Municipal Mário Gatti	Instituto Adolfo Lutz, Interdiciplinary Procedures Center, Strategic Laboratory	Claudio Tavares Sacchi, Claudia Regina Gonçalves, Erica Valessa Ramos Gomes, Karoline Rodrigues Campos
EPI_ISL_623119, EPI_ISL_623143, EPI_ISL_623144, EPI_ISL_623145, EPI_ISL_623147, EPI_ISL_623149, EPI_ISL_623158, EPI_ISL_623161, EPI_ISL_623165	Laboratorio de Virologia Molecular / UFRJ	Bioinformatics Laboratory / LNCC	Carolina M Voloch, Ronaldo S Francisco Jr, Luiz G P de Almeida, Otavio J. Brustolini, Cynthia C Cardoso, Alexandra L Gerber, Ana Paula de C Guimarães, Diana Mariani, Covid19-UFRJ Workgroup, Luís Cristóvão Pôrto, Renato S Aguiar, Terezinha M P P Castiñeiras, Orlando C. Ferreira, Amilcar Tanuri, Ana Tereza R de Vasconcelos
EPI_ISL_636737, EPI_ISL_636835, EPI_ISL_636837	Laboratório de Imunofarmacologia - Instituto Oswaldo Cruz	Laboratório de Imunofarmacologia - Instituto Oswaldo Cruz	Souza,T.M., Fintelman-Rodrigues,N., De Paula,A.D., Saraiva,F.B., Ferreira,M.A. and Sacramento,C.Q.
EPI_ISL_693196	Hospital Santa Clara	Instituto Adolfo Lutz, Interdiciplinary Procedures Center, Strategic Laboratory	Claudio Tavares Sacchi, Claudia Regina Gonçalves, Erica Valessa Ramos Gomes, Karoline Rodrigues Campos
EPI_ISL_693198	Santa Casa de Misericordia de Sao Paulo - Hospital Central	Instituto Adolfo Lutz, Interdiciplinary Procedures Center, Strategic Laboratory	Claudio Tavares Sacchi, Claudia Regina Gonçalves, Erica Valessa Ramos Gomes, Karoline Rodrigues Campos
EPI_ISL_693199	Hospital do Servidor Publico Estadual Francisco Morato de Oliveira	Instituto Adolfo Lutz, Interdiciplinary Procedures Center, Strategic Laboratory	Claudio Tavares Sacchi, Claudia Regina Gonçalves, Erica Valessa Ramos Gomes, Karoline Rodrigues Campos
EPI_ISL_693204	Pronto Socorro Dr. Conrado Cesarino Nuvolini	Instituto Adolfo Lutz, Interdiciplinary Procedures Center, Strategic Laboratory	Claudio Tavares Sacchi, Claudia Regina Gonçalves, Erica Valessa Ramos Gomes, Karoline Rodrigues Campos
EPI_ISL_693205	Hospital de Campanha Covid-19 Assis	Instituto Adolfo Lutz, Interdiciplinary Procedures Center, Strategic Laboratory	Claudio Tavares Sacchi, Claudia Regina Gonçalves, Erica Valessa Ramos Gomes, Karoline Rodrigues Campos
EPI_ISL_693206	Hospital Municipal Mario Gatti	Instituto Adolfo Lutz, Interdiciplinary Procedures Center, Strategic Laboratory	Claudio Tavares Sacchi, Claudia Regina Gonçalves, Erica Valessa Ramos Gomes, Karoline Rodrigues Campos
EPI_ISL_693207	Cs II Doutor Antonio Vicoso Moreira de Rezende	Instituto Adolfo Lutz, Interdiciplinary Procedures Center, Strategic Laboratory	Claudio Tavares Sacchi, Claudia Regina Gonçalves, Erica Valessa Ramos Gomes, Karoline Rodrigues Campos
EPI_ISL_693208, EPI_ISL_693209	Hospital Municipal Antonio Giglio	Instituto Adolfo Lutz, Interdiciplinary Procedures Center, Strategic Laboratory	Claudio Tavares Sacchi, Claudia Regina Gonçalves, Erica Valessa Ramos Gomes, Karoline Rodrigues Campos
EPI_ISL_693210	Pronto-Socorro Dr. Osmar Mesquita	Instituto Adolfo Lutz, Interdiciplinary Procedures Center, Strategic Laboratory	Claudio Tavares Sacchi, Claudia Regina Gonçalves, Erica Valessa Ramos Gomes, Karoline Rodrigues Campos
EPI_ISL_693211	Santa Casa de Misericordia e Maternidade	Instituto Adolfo Lutz, Interdiciplinary Procedures Center, Strategic Laboratory	Claudio Tavares Sacchi, Claudia Regina Gonçalves, Erica Valessa Ramos Gomes, Karoline Rodrigues Campos
EPI_ISL_693212	Santa Casa de Misericordia de Braganca Paulista	Instituto Adolfo Lutz, Interdiciplinary Procedures Center, Strategic Laboratory	Claudio Tavares Sacchi, Claudia Regina Gonçalves, Erica Valessa Ramos Gomes, Karoline Rodrigues Campos
EPI_ISL_693213	Hospital E Maternidade Municipal Governador Mario Covas	Instituto Adolfo Lutz, Interdiciplinary Procedures Center, Strategic Laboratory	Claudio Tavares Sacchi, Claudia Regina Gonçalves, Erica Valessa Ramos Gomes, Karoline Rodrigues Campos
EPI_ISL_693214	Unidade de Pronto Atendimento Central de Caraguatatuba	Instituto Adolfo Lutz, Interdiciplinary Procedures Center, Strategic Laboratory	Claudio Tavares Sacchi, Claudia Regina Gonçalves, Erica Valessa Ramos Gomes, Karoline Rodrigues Campos
EPI_ISL_693215	Secretaria Municipal de Saúde de Iracemapolis	Instituto Adolfo Lutz, Interdiciplinary Procedures Center, Strategic Laboratory	Claudio Tavares Sacchi, Claudia Regina Gonçalves, Erica Valessa Ramos Gomes, Karoline Rodrigues Campos
EPI_ISL_693216, EPI_ISL_693217	Unidade de Vigilância Epiudemiológica de Araras	Instituto Adolfo Lutz, Interdiciplinary Procedures Center, Strategic Laboratory	Claudio Tavares Sacchi, Claudia Regina Gonçalves, Erica Valessa Ramos Gomes, Karoline Rodrigues Campos
EPI_ISL_693218	Hospital Domingos Leonardo Ceravolo Presidente Prudente	Instituto Adolfo Lutz, Interdiciplinary Procedures Center, Strategic Laboratory	Claudio Tavares Sacchi, Claudia Regina Gonçalves, Erica Valessa Ramos Gomes, Karoline Rodrigues Campos
EPI_ISL_693219	Santa Casa da Misericórdia de Presidente Prudente	Instituto Adolfo Lutz, Interdiciplinary Procedures Center, Strategic Laboratory	Claudio Tavares Sacchi, Claudia Regina Gonçalves, Erica Valessa Ramos Gomes, Karoline Rodrigues Campos
EPI_ISL_693220	Laboratório Municipal de Piracicaba	Instituto Adolfo Lutz, Interdiciplinary Procedures Center, Strategic Laboratory	Claudio Tavares Sacchi, Claudia Regina Gonçalves, Erica Valessa Ramos Gomes, Karoline Rodrigues Campos
EPI_ISL_693221, EPI_ISL_693222	Secretaria Municipal de Saúde de Birigui	Instituto Adolfo Lutz, Interdiciplinary Procedures Center, Strategic Laboratory	Claudio Tavares Sacchi, Claudia Regina Gonçalves, Erica Valessa Ramos Gomes, Karoline Rodrigues Campos
EPI_ISL_693223, EPI_ISL_693224	Laboratório Municipal de Piracicaba	Instituto Adolfo Lutz, Interdiciplinary Procedures Center, Strategic Laboratory	Claudio Tavares Sacchi, Claudia Regina Gonçalves, Erica Valessa Ramos Gomes, Karoline Rodrigues Campos
EPI_ISL_693225	Ubs Vila Rosa - Olímpia Gomes De Almeida	Instituto Adolfo Lutz, Interdiciplinary Procedures Center, Strategic Laboratory	Claudio Tavares Sacchi, Claudia Regina Gonçalves, Erica Valessa Ramos Gomes, Karoline Rodrigues Campos
EPI_ISL_693226	Unidade de Pronto Atendimento Sao José	Instituto Adolfo Lutz, Interdiciplinary Procedures Center, Strategic Laboratory	Claudio Tavares Sacchi, Claudia Regina Gonçalves, Erica Valessa Ramos Gomes, Karoline Rodrigues Campos
EPI_ISL_693227	UBS Vila Marchi	Instituto Adolfo Lutz, Interdiciplinary Procedures Center,	Claudio Tavares Sacchi, Claudia Regina Gonçalves, Erica Valessa Ramos Gomes, Karoline Rodrigues Campos

		Strategic Laboratory	
EPI_ISL_693228	Secretaria Municipal de Sorocaba	Instituto Adolfo Lutz, Interdiciplinary Procedures Center, Strategic Laboratory	Claudio Tavares Sacchi, Claudia Regina Gonçalves, Erica Valessa Ramos Gomes, Karoline Rodrigues Campos
EPI_ISL_693229	Hospital 8 de Maio	Instituto Adolfo Lutz, Interdiciplinary Procedures Center, Strategic Laboratory	Claudio Tavares Sacchi, Claudia Regina Gonçalves, Erica Valessa Ramos Gomes, Karoline Rodrigues Campos
EPI_ISL_693230	Hospital e Pronto Socorro Portinari	Instituto Adolfo Lutz, Interdiciplinary Procedures Center, Strategic Laboratory	Claudio Tavares Sacchi, Claudia Regina Gonçalves, Erica Valessa Ramos Gomes, Karoline Rodrigues Campos
EPI_ISL_693231	Pronto Socorro Municipal de Santa Branca	Instituto Adolfo Lutz, Interdiciplinary Procedures Center, Strategic Laboratory	Claudio Tavares Sacchi, Claudia Regina Gonçalves, Erica Valessa Ramos Gomes, Karoline Rodrigues Campos
EPI_ISL_693232	Hospital e Pronto Socorro Portinari	Instituto Adolfo Lutz, Interdiciplinary Procedures Center, Strategic Laboratory	Claudio Tavares Sacchi, Claudia Regina Gonçalves, Erica Valessa Ramos Gomes, Karoline Rodrigues Campos
EPI_ISL_693233	Hospital Santa Cruz	Instituto Adolfo Lutz, Interdiciplinary Procedures Center, Strategic Laboratory	Claudio Tavares Sacchi, Claudia Regina Gonçalves, Erica Valessa Ramos Gomes, Karoline Rodrigues Campos
EPI_ISL_693234	Upa Vereador Jose Da Rocha Goncalves	Instituto Adolfo Lutz, Interdiciplinary Procedures Center, Strategic Laboratory	Claudio Tavares Sacchi, Claudia Regina Gonçalves, Erica Valessa Ramos Gomes, Karoline Rodrigues Campos
EPI_ISL_693235	Casmi Centro Atendimento Saude da Mulher e Infancia	Instituto Adolfo Lutz, Interdiciplinary Procedures Center, Strategic Laboratory	Claudio Tavares Sacchi, Claudia Regina Gonçalves, Erica Valessa Ramos Gomes, Karoline Rodrigues Campos
EPI_ISL_693236	Hospital Santa Marcelina Sao Paulo	Instituto Adolfo Lutz, Interdiciplinary Procedures Center, Strategic Laboratory	Claudio Tavares Sacchi, Claudia Regina Gonçalves, Erica Valessa Ramos Gomes, Karoline Rodrigues Campos
EPI_ISL_693237	UPA Santa Isabel	Instituto Adolfo Lutz, Interdiciplinary Procedures Center, Strategic Laboratory	Claudio Tavares Sacchi, Claudia Regina Gonçalves, Erica Valessa Ramos Gomes, Karoline Rodrigues Campos
EPI_ISL_693238, EPI_ISL_693239	Secao Centro de Diagnostico Secedi	Instituto Adolfo Lutz, Interdiciplinary Procedures Center, Strategic Laboratory	Claudio Tavares Sacchi, Claudia Regina Gonçalves, Erica Valessa Ramos Gomes, Karoline Rodrigues Campos
EPI_ISL_693240	Centro de Vigilância a Saude de Diadema	Instituto Adolfo Lutz, Interdiciplinary Procedures Center, Strategic Laboratory	Claudio Tavares Sacchi, Claudia Regina Gonçalves, Erica Valessa Ramos Gomes, Karoline Rodrigues Campos
EPI_ISL_693241	Hospital e Maternidade Sao Lucas	Instituto Adolfo Lutz, Interdiciplinary Procedures Center, Strategic Laboratory	Claudio Tavares Sacchi, Claudia Regina Gonçalves, Erica Valessa Ramos Gomes, Karoline Rodrigues Campos
EPI_ISL_693242	Centro de Vigilância a Saude de Diadema	Instituto Adolfo Lutz, Interdiciplinary Procedures Center, Strategic Laboratory	Claudio Tavares Sacchi, Claudia Regina Gonçalves, Erica Valessa Ramos Gomes, Karoline Rodrigues Campos
EPI_ISL_693243	Laboratório Municipal de Piracicaba	Instituto Adolfo Lutz, Interdiciplinary Procedures Center, Strategic Laboratory	Claudio Tavares Sacchi, Claudia Regina Gonçalves, Erica Valessa Ramos Gomes, Karoline Rodrigues Campos
EPI_ISL_693244	Centro Médico da Polícia Militar do Estado de Sao Paulo	Instituto Adolfo Lutz, Interdiciplinary Procedures Center, Strategic Laboratory	Claudio Tavares Sacchi, Claudia Regina Gonçalves, Erica Valessa Ramos Gomes, Karoline Rodrigues Campos
EPI_ISL_693245	UPA Santa Isabel	Instituto Adolfo Lutz, Interdiciplinary Procedures Center, Strategic Laboratory	Claudio Tavares Sacchi, Claudia Regina Gonçalves, Erica Valessa Ramos Gomes, Karoline Rodrigues Campos
EPI_ISL_693246	Laboratorio Municipal de Rio Grande da Serra	Instituto Adolfo Lutz, Interdiciplinary Procedures Center, Strategic Laboratory	Claudio Tavares Sacchi, Claudia Regina Gonçalves, Erica Valessa Ramos Gomes, Karoline Rodrigues Campos
EPI_ISL_693247	Secao Centro de Diagnostico Secedi	Instituto Adolfo Lutz, Interdiciplinary Procedures Center, Strategic Laboratory	Claudio Tavares Sacchi, Claudia Regina Gonçalves, Erica Valessa Ramos Gomes, Karoline Rodrigues Campos
EPI_ISL_693248	Centro Municipal de Epidemiologia e Imunizações	Instituto Adolfo Lutz, Interdiciplinary Procedures Center, Strategic Laboratory	Claudio Tavares Sacchi, Claudia Regina Gonçalves, Erica Valessa Ramos Gomes, Karoline Rodrigues Campos
EPI_ISL_708529	Secretária Municipal de Saude de Fernandópolis	Instituto Adolfo Lutz, Interdiciplinary Procedures Center, Strategic Laboratory	Claudio Tavares Sacchi, Claudia Regina Gonçalves, Erica Valessa Ramos Gomes, Carlos Henrique Camargo, Karoline Rodrigues Campos, Fernanda Modesto Tolentino Binhardi, Maricelia Navarro Pinheiro Flores, Marcia Maria Costa Nunes Soares, Janaina Other Martins Montanha
EPI_ISL_717806	Laboratorio de Virologia Molecular / UFRJ	Bioinformatics Laboratory / LNCC	Carolina M Voloch, Ronaldo da Silva F Jr, Luiz G P de Almeida, Cynthia C Cardoso, Otavio Bustrolini, Alexandra L Gerber, Ana Paula de C Guimarães, Diana Mariani, Andréa Cony Cavalcanti, Claudia dos Santos Rodrigues, Terezinha M P P Castiñeira, Amilcar Tanuri, Ana Tereza R de Vasconcelos
EPI_ISL_717809	LACEN Dr. Francisco Rimolo Neto	Bioinformatics Laboratory / LNCC	Carolina M Voloch, Ronaldo da Silva F Jr, Luiz G P de Almeida, Cynthia C Cardoso, Otavio Bustrolini, Alexandra L Gerber, Ana Paula de C Guimarães, Diana Mariani, Andréa Cony Cavalcanti, Claudia dos Santos Rodrigues, Terezinha M P P Castiñeira, Amilcar Tanuri, Ana Tereza R de Vasconcelos
EPI_ISL_717812, EPI_ISL_717813, EPI_ISL_717814, EPI_ISL_717815, EPI_ISL_717816, EPI_ISL_717817	Laboratorio de Virologia Molecular / UFRJ	Bioinformatics Laboratory / LNCC	Carolina M Voloch, Ronaldo da Silva F Jr, Luiz G P de Almeida, Cynthia C Cardoso, Otavio Bustrolini, Alexandra L Gerber, Ana Paula de C Guimarães, Diana Mariani, Andréa Cony Cavalcanti, Claudia dos Santos Rodrigues, Terezinha M P P Castiñeira, Amilcar Tanuri, Ana Tereza R de Vasconcelos
EPI_ISL_717832, EPI_ISL_717833, EPI_ISL_717834, EPI_ISL_717835, EPI_ISL_717836, EPI_ISL_717841	LACEN Dr. Francisco Rimolo Neto	Bioinformatics Laboratory / LNCC	Carolina M Voloch, Ronaldo da Silva F Jr, Luiz G P de Almeida, Cynthia C Cardoso, Otavio Bustrolini, Alexandra L Gerber, Ana Paula de C Guimarães, Diana Mariani, Andréa Cony Cavalcanti, Claudia dos Santos Rodrigues, Terezinha M P P Castiñeira, Amilcar Tanuri, Ana Tereza R de Vasconcelos
EPI_ISL_717845, EPI_ISL_717846, EPI_ISL_717847, EPI_ISL_717848, EPI_ISL_717849, EPI_ISL_717850, EPI_ISL_717851, EPI_ISL_717852, EPI_ISL_717853, EPI_ISL_717854, EPI_ISL_717855, EPI_ISL_717856, EPI_ISL_717857, EPI_ISL_717858, EPI_ISL_717859, EPI_ISL_717860, EPI_ISL_717861, EPI_ISL_717862, EPI_ISL_717863, EPI_ISL_717864, EPI_ISL_717865, EPI_ISL_717866, EPI_ISL_717867, EPI_ISL_717868, EPI_ISL_717869, EPI_ISL_717870, EPI_ISL_717871, EPI_ISL_717872, EPI_ISL_717873			
see above	Laboratorio de Virologia Molecular / UFRJ	Bioinformatics Laboratory / LNCC	Carolina M Voloch, Ronaldo da Silva F Jr, Luiz G P de Almeida, Cynthia C Cardoso, Otavio Bustrolini, Alexandra L Gerber, Ana Paula de C Guimarães, Diana Mariani, Andréa Cony Cavalcanti, Claudia dos Santos Rodrigues, Terezinha M P P Castiñeira, Amilcar Tanuri, Ana Tereza R de Vasconcelos
EPI_ISL_717899, EPI_ISL_717900, EPI_ISL_717901, EPI_ISL_717902, EPI_ISL_717903, EPI_ISL_717904, EPI_ISL_717905, EPI_ISL_717906, EPI_ISL_717907, EPI_ISL_717908, EPI_ISL_717909			
see above	LACEN RJ - Noel Nutels	Bioinformatics Laboratory / LNCC	Carolina M Voloch, Ronaldo da Silva F Jr, Luiz G P de Almeida, Cynthia C Cardoso, Otavio Bustrolini, Alexandra L Gerber, Ana Paula de C Guimarães, Diana Mariani, Andréa Cony Cavalcanti, Claudia dos Santos Rodrigues, Terezinha M P P Castiñeira, Amilcar Tanuri, Ana Tereza R de Vasconcelos
EPI_ISL_717910, EPI_ISL_717911, EPI_ISL_717912, EPI_ISL_717913, EPI_ISL_717914, EPI_ISL_717915, EPI_ISL_717916, EPI_ISL_717917, EPI_ISL_717918, EPI_ISL_717919, EPI_ISL_717958			
see above	LACEN Dr. Francisco Rimolo Neto	Bioinformatics Laboratory / LNCC	Carolina M Voloch, Ronaldo da Silva F Jr, Luiz G P de Almeida, Cynthia C Cardoso, Otavio Bustrolini, Alexandra L Gerber, Ana Paula de C Guimarães, Diana Mariani, Andréa Cony Cavalcanti, Claudia dos Santos Rodrigues, Terezinha M P P Castiñeira, Amilcar Tanuri, Ana Tereza R de Vasconcelos
EPI_ISL_717959	Laboratorio de Virologia Molecular / UFRJ	Bioinformatics Laboratory / LNCC	Carolina M Voloch, Ronaldo da Silva F Jr, Luiz G P de Almeida, Cynthia C Cardoso, Otavio Bustrolini, Alexandra L Gerber, Ana Paula de C Guimarães, Diana Mariani, Andréa Cony Cavalcanti, Claudia dos Santos Rodrigues, Terezinha M P P Castiñeira, Amilcar Tanuri, Ana Tereza R de Vasconcelos
EPI_ISL_717962	LACEN RJ - Noel Nutels	Bioinformatics Laboratory / LNCC	Carolina M Voloch, Ronaldo da Silva F Jr, Luiz G P de Almeida, Cynthia C Cardoso, Otavio Bustrolini, Alexandra L Gerber, Ana Paula de C Guimarães, Diana Mariani, Andréa Cony Cavalcanti, Claudia dos Santos Rodrigues, Terezinha M P P Castiñeira, Amilcar Tanuri, Ana Tereza R de Vasconcelos
EPI_ISL_717963, EPI_ISL_717964	LACEN Dr. Francisco Rimolo Neto	Bioinformatics Laboratory / LNCC	Carolina M Voloch, Ronaldo da Silva F Jr, Luiz G P de Almeida, Cynthia C Cardoso, Otavio Bustrolini, Alexandra L Gerber, Ana Paula de C Guimarães, Diana Mariani, Andréa Cony Cavalcanti, Claudia dos Santos Rodrigues, Terezinha M P P Castiñeira, Amilcar Tanuri, Ana Tereza R de Vasconcelos
EPI_ISL_729794, EPI_ISL_729800, EPI_ISL_729801, EPI_ISL_729802, EPI_ISL_729803, EPI_ISL_729820, EPI_ISL_729821, EPI_ISL_729822, EPI_ISL_729823, EPI_ISL_729824, EPI_ISL_729825, EPI_ISL_729826, EPI_ISL_729827, EPI_ISL_729828, EPI_ISL_729829, EPI_ISL_729830, EPI_ISL_729831, EPI_ISL_729832,			

EPI_ISL_729833, EPI_ISL_729835, EPI_ISL_729836, EPI_ISL_729837, EPI_ISL_729838, EPI_ISL_729839, EPI_ISL_729840, EPI_ISL_729841, EPI_ISL_729842, EPI_ISL_729843, EPI_ISL_729844, EPI_ISL_729845, EPI_ISL_729846, EPI_ISL_729847, EPI_ISL_729848, EPI_ISL_729849, EPI_ISL_729850, EPI_ISL_729851, EPI_ISL_729852, EPI_ISL_729853, EPI_ISL_729854, EPI_ISL_729855, EPI_ISL_729856, EPI_ISL_729857, EPI_ISL_729858, EPI_ISL_729859, EPI_ISL_729860, EPI_ISL_729861

see above	Laboratório Central de Saúde Pública do Estado do Rio Grande do Sul (LACEN-RS)	Laboratory of Respiratory Viruses and Measles, Oswaldo Cruz Institute, FIOCRUZ	Paola Resende, Luciana Appolinario, Fernando Motta, Anna Carolina Paixão, Ana Carolina Mendonça, Tatiana Schaffer Gregianini, Marilda Tereza Mar da Rosa, Marilda Siqueira on behalf of the Fiocruz COVID-19 Genomic Surveillance Network
EPI_ISL_735396	Hospital de Camplanha COVID 19 SER	Instituto Adolfo Lutz, Interdiciplinary Procedures Center, Strategic Laboratory	Claudio Tavares Sacchi, Claudia Regina Gonçalves, Erica Valessa Ramos Gomes, Karoline Rodrigues Campos
EPI_ISL_735397	Unidade Respiratória Nova Hortolandia	Instituto Adolfo Lutz, Interdiciplinary Procedures Center, Strategic Laboratory	Claudio Tavares Sacchi, Claudia Regina Gonçalves, Erica Valessa Ramos Gomes, Karoline Rodrigues Campos
EPI_ISL_735398	Laboratorio Fleury	Instituto Adolfo Lutz, Interdiciplinary Procedures Center, Strategic Laboratory	Claudio Tavares Sacchi, Claudia Regina Gonçalves, Erica Valessa Ramos Gomes, Karoline Rodrigues Campos
EPI_ISL_735400	Instituto Adolfo Lutz - Regional de Santos	Instituto Adolfo Lutz, Interdiciplinary Procedures Center, Strategic Laboratory	Claudio Tavares Sacchi, Claudia Regina Gonçalves, Erica Valessa Ramos Gomes, Karoline Rodrigues Campos
EPI_ISL_735401, EPI_ISL_735402, EPI_ISL_735403, EPI_ISL_735404	Instituto Adolfo Lutz - Regional de Rio Claro	Instituto Adolfo Lutz, Interdiciplinary Procedures Center, Strategic Laboratory	Claudio Tavares Sacchi, Claudia Regina Gonçalves, Erica Valessa Ramos Gomes, Karoline Rodrigues Campos
EPI_ISL_735405	Secretaria Minucipal de Saude de Birigui	Instituto Adolfo Lutz, Interdiciplinary Procedures Center, Strategic Laboratory	Claudio Tavares Sacchi, Claudia Regina Gonçalves, Erica Valessa Ramos Gomes, Karoline Rodrigues Campos
EPI_ISL_735406	Unidade de Pronto Atendimento UPA I Sta Isabel	Instituto Adolfo Lutz, Interdiciplinary Procedures Center, Strategic Laboratory	Claudio Tavares Sacchi, Claudia Regina Gonçalves, Erica Valessa Ramos Gomes, Karoline Rodrigues Campos
EPI_ISL_735407	Santa Casa de Marilia	Instituto Adolfo Lutz, Interdiciplinary Procedures Center, Strategic Laboratory	Claudio Tavares Sacchi, Claudia Regina Gonçalves, Erica Valessa Ramos Gomes, Karoline Rodrigues Campos
EPI_ISL_735408	COVID 19 Centro de Combate ao Coronavirus CCC Jandira	Instituto Adolfo Lutz, Interdiciplinary Procedures Center, Strategic Laboratory	Claudio Tavares Sacchi, Claudia Regina Gonçalves, Erica Valessa Ramos Gomes, Karoline Rodrigues Campos
EPI_ISL_735409	Unidade de Pronto Atendimento Carlos Lourenco	Instituto Adolfo Lutz, Interdiciplinary Procedures Center, Strategic Laboratory	Claudio Tavares Sacchi, Claudia Regina Gonçalves, Erica Valessa Ramos Gomes, Karoline Rodrigues Campos
EPI_ISL_735410	Instituto Adolfo Lutz - Regional de Rio Claro	Instituto Adolfo Lutz, Interdiciplinary Procedures Center, Strategic Laboratory	Claudio Tavares Sacchi, Claudia Regina Gonçalves, Erica Valessa Ramos Gomes, Karoline Rodrigues Campos
EPI_ISL_735411	Centro de Vigilancia a Saude de Diadema	Instituto Adolfo Lutz, Interdiciplinary Procedures Center, Strategic Laboratory	Claudio Tavares Sacchi, Claudia Regina Gonçalves, Erica Valessa Ramos Gomes, Karoline Rodrigues Campos
EPI_ISL_735412	Hospital e Pronto Socorro Portinari	Instituto Adolfo Lutz, Interdiciplinary Procedures Center, Strategic Laboratory	Claudio Tavares Sacchi, Claudia Regina Gonçalves, Erica Valessa Ramos Gomes, Karoline Rodrigues Campos
EPI_ISL_735413	Militello Centro de Diagnosticos e Biopesquisa Clinica	Instituto Adolfo Lutz, Interdiciplinary Procedures Center, Strategic Laboratory	Claudio Tavares Sacchi, Claudia Regina Gonçalves, Erica Valessa Ramos Gomes, Karoline Rodrigues Campos
EPI_ISL_735414, EPI_ISL_735415	Unidade de Pronto Atendimento de Agenor de Campos	Instituto Adolfo Lutz, Interdiciplinary Procedures Center, Strategic Laboratory	Claudio Tavares Sacchi, Claudia Regina Gonçalves, Erica Valessa Ramos Gomes, Karoline Rodrigues Campos
EPI_ISL_735416	Centro de Saude II Dr Jose Paione Mococa	Instituto Adolfo Lutz, Interdiciplinary Procedures Center, Strategic Laboratory	Claudio Tavares Sacchi, Claudia Regina Gonçalves, Erica Valessa Ramos Gomes, Karoline Rodrigues Campos
EPI_ISL_735417	Unidade de Pronto Atendimento de Agenor de Campos	Instituto Adolfo Lutz, Interdiciplinary Procedures Center, Strategic Laboratory	Claudio Tavares Sacchi, Claudia Regina Gonçalves, Erica Valessa Ramos Gomes, Karoline Rodrigues Campos
EPI_ISL_735418	Hospital Regional do Vale do Paraiba	Instituto Adolfo Lutz, Interdiciplinary Procedures Center, Strategic Laboratory	Claudio Tavares Sacchi, Claudia Regina Gonçalves, Erica Valessa Ramos Gomes, Karoline Rodrigues Campos
EPI_ISL_735419	UBS Alvarenga	Instituto Adolfo Lutz, Interdiciplinary Procedures Center, Strategic Laboratory	Claudio Tavares Sacchi, Claudia Regina Gonçalves, Erica Valessa Ramos Gomes, Karoline Rodrigues Campos
EPI_ISL_735420	UBS Riacho Grande	Instituto Adolfo Lutz, Interdiciplinary Procedures Center, Strategic Laboratory	Claudio Tavares Sacchi, Claudia Regina Gonçalves, Erica Valessa Ramos Gomes, Karoline Rodrigues Campos
EPI_ISL_735421	UBS Sta Terezinha	Instituto Adolfo Lutz, Interdiciplinary Procedures Center, Strategic Laboratory	Claudio Tavares Sacchi, Claudia Regina Gonçalves, Erica Valessa Ramos Gomes, Karoline Rodrigues Campos
EPI_ISL_735422	UBS Dematchi	Instituto Adolfo Lutz, Interdiciplinary Procedures Center, Strategic Laboratory	Claudio Tavares Sacchi, Claudia Regina Gonçalves, Erica Valessa Ramos Gomes, Karoline Rodrigues Campos
EPI_ISL_735423, EPI_ISL_735424	Centro de Vigilancia a Saude de Diadema	Instituto Adolfo Lutz, Interdiciplinary Procedures Center, Strategic Laboratory	Claudio Tavares Sacchi, Claudia Regina Gonçalves, Erica Valessa Ramos Gomes, Karoline Rodrigues Campos
EPI_ISL_735425	Hospital e Maternidade Sao Lucas	Instituto Adolfo Lutz, Interdiciplinary Procedures Center, Strategic Laboratory	Claudio Tavares Sacchi, Claudia Regina Gonçalves, Erica Valessa Ramos Gomes, Karoline Rodrigues Campos
EPI_ISL_735426	Centro de Vigilancia a Saude de Diadema	Instituto Adolfo Lutz, Interdiciplinary Procedures Center, Strategic Laboratory	Claudio Tavares Sacchi, Claudia Regina Gonçalves, Erica Valessa Ramos Gomes, Karoline Rodrigues Campos
EPI_ISL_735427	Instituto Adolfo Lutz - Regional de Santos	Instituto Adolfo Lutz, Interdiciplinary Procedures Center, Strategic Laboratory	Claudio Tavares Sacchi, Claudia Regina Gonçalves, Erica Valessa Ramos Gomes, Karoline Rodrigues Campos
EPI_ISL_735428, EPI_ISL_735429	Hospital Nipo Brasileiro	Instituto Adolfo Lutz, Interdiciplinary Procedures Center, Strategic Laboratory	Claudio Tavares Sacchi, Claudia Regina Gonçalves, Erica Valessa Ramos Gomes, Karoline Rodrigues Campos
EPI_ISL_735430	Instituto Adolfo Lutz - Regional de Santos	Instituto Adolfo Lutz, Interdiciplinary Procedures Center, Strategic Laboratory	Claudio Tavares Sacchi, Claudia Regina Gonçalves, Erica Valessa Ramos Gomes, Karoline Rodrigues Campos
EPI_ISL_735431, EPI_ISL_735432	Hospital Nipo Brasileiro	Instituto Adolfo Lutz, Interdiciplinary Procedures Center, Strategic Laboratory	Claudio Tavares Sacchi, Claudia Regina Gonçalves, Erica Valessa Ramos Gomes, Karoline Rodrigues Campos
EPI_ISL_735433	Posto de Atendimento Saude Cidade Pasc Cajati	Instituto Adolfo Lutz, Interdiciplinary Procedures Center, Strategic Laboratory	Claudio Tavares Sacchi, Claudia Regina Gonçalves, Erica Valessa Ramos Gomes, Karoline Rodrigues Campos
EPI_ISL_756293	Center for Biotechnology and Cell Therapy, São Rafael Hospital, Salvador, Brazil	Center for Biotechnology and Cell Therapy, São Rafael Hospital, Salvador, Brazil	Carolina Kymie Vasques Nonaka, Marília Miranda Franco, Tiago Gräf, Ana Verena Almeida Mendes, Renato Santana de Aguiar, Marta Giovanetti, Bruno Solano de Freitas Souza
EPI_ISL_776764, EPI_ISL_776765	Instituto Adolfo Lutz - Regional de Santo Andre	Instituto Adolfo Lutz, Interdiciplinary Procedures Center, Strategic Laboratory	Claudio Tavares Sacchi, Claudia Regina Gonçalves, Erica Valessa Ramos Gomes, Karoline Rodrigues Campos
EPI_ISL_792114	Instituto Adolfo Lutz - Central	Instituto Adolfo Lutz, Interdiciplinary Procedures Center, Strategic Laboratory	Claudio Tavares Sacchi, Claudia Regina Gonçalves, Erica Valessa Ramos Gomes, Karoline Rodrigues Campos

EPI_ISL_792115, EPI_ISL_792116	Instituto Adolfo Lutz - Regional de Taubate	Instituto Adolfo Lutz, Interdiciplinary Procedures Center, Strategic Laboratory	Claudio Tavares Sacchi, Claudia Regina Gonçalves, Erica Valessa Ramos Gomes, Karoline Rodrigues Campos
EPI_ISL_792561, EPI_ISL_792563, EPI_ISL_792564, EPI_ISL_792565, EPI_ISL_792566, EPI_ISL_792567, EPI_ISL_792568, EPI_ISL_792569, EPI_ISL_792593, EPI_ISL_792594, EPI_ISL_792595, EPI_ISL_792596, EPI_ISL_792597, EPI_ISL_792602, EPI_ISL_792604, EPI_ISL_792605, EPI_ISL_792606, EPI_ISL_792607, EPI_ISL_792608, EPI_ISL_792609, EPI_ISL_792610, EPI_ISL_792611, EPI_ISL_792612, EPI_ISL_792613, EPI_ISL_792614, EPI_ISL_792615, EPI_ISL_792616, EPI_ISL_792617, EPI_ISL_792618, EPI_ISL_792619, EPI_ISL_792620, EPI_ISL_792621			
see above	Laboratório Central de Saúde Pública do Estado da Paraíba (LACEN-PB)	Laboratory of Respiratory Viruses and Measles, Oswaldo Cruz Institute, FIOCRUZ	Paola Resende, Luciana Appolinario, Fernando Motta, Anna Carolina Paixao, Ana Carolina Mendonca, João Felipe Bezerra, Romero Henrique Teixeira de Vasconcelos, Dalane Loudal Florentino Teixeira, Thiago Franco de Oliveira Carneiro, Marilda Siqueira on behalf of the Fiocruz COVID-19 Genomic Surveillance Network
EPI_ISL_792640, EPI_ISL_792644	Laboratório Central de Saúde Pública do Estado de Alagoas (LACEN-AL)	Laboratory of Respiratory Viruses and Measles, Oswaldo Cruz Institute, FIOCRUZ	Paola Resende, Luciana Appolinario, Fernando Motta, Anna Carolina Paixao, Ana Carolina Mendonca, Anderson Brandao Leite, Marilda Siqueira on behalf of the Fiocruz COVID-19 Genomic Surveillance Network
EPI_ISL_801386, EPI_ISL_801387, EPI_ISL_801388	Laboratorio de Ecologia de Doencas Transmissiveis na Amazonia, Instituto Leonidas e Maria Deane - Fiocruz Amazonia	Laboratorio de Ecologia de Doencas Transmissiveis na Amazonia, Instituto Leonidas e Maria Deane - Fiocruz Amazonia	Valdinete Nascimento, Victor Souza, André Corado, Fernanda Nascimento, George Silva, Ágatha Costa, Debora Duarte, Luciana Gonçalves, Maria Júlia Brandão, Michele Jesus, Felipe Naveca
EPI_ISL_801397, EPI_ISL_801398, EPI_ISL_801399, EPI_ISL_801400, EPI_ISL_801401, EPI_ISL_801402, EPI_ISL_801403	Laboratório Central de Saúde Pública do Amazonas - LACEN-AM	Laboratorio de Ecologia de Doencas Transmissiveis na Amazonia, Instituto Leonidas e Maria Deane - Fiocruz Amazonia	Valdinete Nascimento, Victor Souza, André Corado, Fernanda Nascimento, George Silva, Ágatha Costa, Debora Duarte, Luciana Gonçalves, Maria Júlia Brandão, Michele Jesus, Felipe Naveca
EPI_ISL_831474, EPI_ISL_831645, EPI_ISL_831646, EPI_ISL_831660, EPI_ISL_831678, EPI_ISL_831681, EPI_ISL_831683, EPI_ISL_831685, EPI_ISL_831688, EPI_ISL_831689, EPI_ISL_831892, EPI_ISL_831898, EPI_ISL_831913, EPI_ISL_831938, EPI_ISL_831939, EPI_ISL_831940, EPI_ISL_832009			
see above	Laboratório de Microbiologia Molecular - Universidade FEEVALE	Universidade Federal de Ciências da Saúde de Porto Alegre	Vinícius Bonetti Franceschi, Amanda de Menezes Mayer, Gabriel Dickin Caldana, Carla Andretta Moreira Neves, Patrícia Aline Gröhs Ferrareze, Gabriela Bettella Cybis, Ricardo Ariel Zimerman, Lívia Kmetzsch, Fernando Rosado Spilki, Claudia Elizabeth Thompson
EPI_ISL_833156	Instituto Adolfo Lutz - Regional de Sorocaba	Instituto Adolfo Lutz, Interdiciplinary Procedures Center, Strategic Laboratory	Claudio Tavares Sacchi, Claudia Regina Gonçalves, Erica Valessa Ramos Gomes, Karoline Rodrigues Campos
EPI_ISL_848608, EPI_ISL_848609, EPI_ISL_848610, EPI_ISL_848611, EPI_ISL_848612, EPI_ISL_848613, EPI_ISL_848614, EPI_ISL_848615	Evandro Chagas Institute	Evandro Chagas Institute	Santos, M.C.; Silva, A.M.; Junior, W.D.C.; Barbagelata, L.S.; Ferreira, J.A.; Sousa, E.M.A.; da Silva, P.S.; Pinheiro, K.C.; L.C.; Sousa Junior, E.C.
EPI_ISL_861644	Hospital Santa Virginia	Instituto Adolfo Lutz, Interdiciplinary Procedures Center, Strategic Laboratory	Claudio Tavares Sacchi, Claudia Regina Gonçalves, Erica Valessa Ramos Gomes, Karoline Rodrigues Campos
EPI_ISL_861645	Hospital e Pronto Socorro Comunitario Vila Iolanda	Instituto Adolfo Lutz, Interdiciplinary Procedures Center, Strategic Laboratory	Claudio Tavares Sacchi, Claudia Regina Gonçalves, Erica Valessa Ramos Gomes, Karoline Rodrigues Campos
EPI_ISL_861646, EPI_ISL_861647	Hospital Santa Marcelina Sao Paulo	Instituto Adolfo Lutz, Interdiciplinary Procedures Center, Strategic Laboratory	Claudio Tavares Sacchi, Claudia Regina Gonçalves, Erica Valessa Ramos Gomes, Karoline Rodrigues Campos
EPI_ISL_861648	Hospital e Pronto Socorro Portinari	Instituto Adolfo Lutz, Interdiciplinary Procedures Center, Strategic Laboratory	Claudio Tavares Sacchi, Claudia Regina Gonçalves, Erica Valessa Ramos Gomes, Karoline Rodrigues Campos
EPI_ISL_861649	Hospital Renascença Campinas	Instituto Adolfo Lutz, Interdiciplinary Procedures Center, Strategic Laboratory	Claudio Tavares Sacchi, Claudia Regina Gonçalves, Erica Valessa Ramos Gomes, Karoline Rodrigues Campos
EPI_ISL_861650	Hospital Santa Marcelina Sao Paulo	Instituto Adolfo Lutz, Interdiciplinary Procedures Center, Strategic Laboratory	Claudio Tavares Sacchi, Claudia Regina Gonçalves, Erica Valessa Ramos Gomes, Karoline Rodrigues Campos
EPI_ISL_861651	AMA Jardim Brasil	Instituto Adolfo Lutz, Interdiciplinary Procedures Center, Strategic Laboratory	Claudio Tavares Sacchi, Claudia Regina Gonçalves, Erica Valessa Ramos Gomes, Karoline Rodrigues Campos
EPI_ISL_861652	AMA Wamberto Dias da Costa	Instituto Adolfo Lutz, Interdiciplinary Procedures Center, Strategic Laboratory	Claudio Tavares Sacchi, Claudia Regina Gonçalves, Erica Valessa Ramos Gomes, Karoline Rodrigues Campos
EPI_ISL_861653	Hospital Santa Virgínia	Instituto Adolfo Lutz, Interdiciplinary Procedures Center, Strategic Laboratory	Claudio Tavares Sacchi, Claudia Regina Gonçalves, Erica Valessa Ramos Gomes, Karoline Rodrigues Campos
EPI_ISL_861654, EPI_ISL_861655	Hospital Santa Marcelina Sao Paulo	Instituto Adolfo Lutz, Interdiciplinary Procedures Center, Strategic Laboratory	Claudio Tavares Sacchi, Claudia Regina Gonçalves, Erica Valessa Ramos Gomes, Karoline Rodrigues Campos
EPI_ISL_861656	UPA de Jandira	Instituto Adolfo Lutz, Interdiciplinary Procedures Center, Strategic Laboratory	Claudio Tavares Sacchi, Claudia Regina Gonçalves, Erica Valessa Ramos Gomes, Karoline Rodrigues Campos
EPI_ISL_861657	Hospital e Maternidade Sino Brasileiro	Instituto Adolfo Lutz, Interdiciplinary Procedures Center, Strategic Laboratory	Claudio Tavares Sacchi, Claudia Regina Gonçalves, Erica Valessa Ramos Gomes, Karoline Rodrigues Campos
EPI_ISL_861658	Hospital Municipal Antônio Giglio	Instituto Adolfo Lutz, Interdiciplinary Procedures Center, Strategic Laboratory	Claudio Tavares Sacchi, Claudia Regina Gonçalves, Erica Valessa Ramos Gomes, Karoline Rodrigues Campos
EPI_ISL_861659, EPI_ISL_861660, EPI_ISL_861661	PS e Maternidade Nair Fonseca Leitao Arantes	Instituto Adolfo Lutz, Interdiciplinary Procedures Center, Strategic Laboratory	Claudio Tavares Sacchi, Claudia Regina Gonçalves, Erica Valessa Ramos Gomes, Karoline Rodrigues Campos
EPI_ISL_861662	CS I Tacito Leite de Carvalho e Silva	Instituto Adolfo Lutz, Interdiciplinary Procedures Center, Strategic Laboratory	Claudio Tavares Sacchi, Claudia Regina Gonçalves, Erica Valessa Ramos Gomes, Karoline Rodrigues Campos
EPI_ISL_861664	Instituto Adolfo Lutz - Regional de Campinas	Instituto Adolfo Lutz, Interdiciplinary Procedures Center, Strategic Laboratory	Claudio Tavares Sacchi, Claudia Regina Gonçalves, Erica Valessa Ramos Gomes, Karoline Rodrigues Campos
EPI_ISL_861665	Instituto Adolfo Lutz - Regional de Taubate	Instituto Adolfo Lutz, Interdiciplinary Procedures Center, Strategic Laboratory	Claudio Tavares Sacchi, Claudia Regina Gonçalves, Erica Valessa Ramos Gomes, Karoline Rodrigues Campos
EPI_ISL_861666	PSF Dr. Antonio Pires de Almeida	Instituto Adolfo Lutz, Interdiciplinary Procedures Center, Strategic Laboratory	Claudio Tavares Sacchi, Claudia Regina Gonçalves, Erica Valessa Ramos Gomes, Karoline Rodrigues Campos
EPI_ISL_861667	Instituto Adolfo Lutz - Regional de Rio Claro	Instituto Adolfo Lutz, Interdiciplinary Procedures Center, Strategic Laboratory	Claudio Tavares Sacchi, Claudia Regina Gonçalves, Erica Valessa Ramos Gomes, Karoline Rodrigues Campos
EPI_ISL_861871, EPI_ISL_861875, EPI_ISL_861884, EPI_ISL_861885, EPI_ISL_861891, EPI_ISL_861895, EPI_ISL_861897, EPI_ISL_861901, EPI_ISL_861910	LATE - Laboratório de Técnicas Especiais - Hospital Israelita Albert Einstein	LATE - Laboratório de Técnicas Especiais - Hospital Israelita Albert Einstein	Deyvid Amgarten, Fernanda de Mello Malta, Raquel Riyuzo, Ana Paula Moreira Salles, Pedro Henrique Sebe Rodrigues, João Renato Rebello Pinho
EPI_ISL_875540, EPI_ISL_875542, EPI_ISL_875543, EPI_ISL_875546, EPI_ISL_875547, EPI_ISL_875548, EPI_ISL_875549	Instituto de Biotecnologia - UNESP-Botucatu-SP	Instituto de Biotecnologia - UNESP-Botucatu-SP	Leila Sabrina Ullmann; Fábio Sossai Possebon, Camila Dantas Malossi, Paula Rahal, Paulo Inacio da Costa, João Pessoa Araújo Jr.

EPI_ISL_882657	LACEN do Estado do Piaui, Dr. Costa Alvarenga	Instituto Adolfo Lutz, Interdiciplinary Procedures Center, Strategic Laboratory	Claudio Tavares Sacchi, Claudia Regina Gonçalves, Erica Valessa Ramos Gomes, Karoline Rodrigues Campos
EPI_ISL_888672	Instituto de Biotecnologia - UNESP-Botucatu-SP	Instituto de Biotecnologia - UNESP-Botucatu-SP	Leila Sabrina Ullmann; Fábio Sossai Possebon, Camila Dantas Malossi, Paula Rahal, Paulo Inacio da Costa, João Pessoa Araújo Jr.
EPI_ISL_906067	PS e Maternidade Nair Fonseca Leitao Arantes	Instituto Adolfo Lutz, Interdiciplinary Procedures Center, Strategic Laboratory	Claudio Tavares Sacchi, Claudia Regina Gonçalves, Erica Valessa Ramos Gomes, Karoline Rodrigues Campos
EPI_ISL_918513	LACEN - Laboratório Central de Saúde Pública do Roraima	Evandro Chagas Institute	Santos, M.C.; Silva, A.M.; Junior, W.D.C.; Barbagelata, L.S.; Ferreira, J.A.; Sousa, E.M.A.; da Silva, P.S.; Pinheiro, K.C.; L.C.; Sousa Junior, E.C.
EPI_ISL_930855, EPI_ISL_930856, EPI_ISL_930857, EPI_ISL_930858	Central Laboratory of Public Health of Rio Grande do Sul (Lacen-RS)	State Center for Health Surveillance of the Health Department of the State of Rio Grande do Sul (CEVS/SES-RS)	Barcellos R, Campos A, Dornelles C, Godinho F, Gonzalez A, Gregianini T, Molina C, Salvato R, Schaurich A,
EPI_ISL_940608	Laboratório Sao Lucas	Instituto Adolfo Lutz, Interdiciplinary Procedures Center, Strategic Laboratory	Claudio Tavares Sacchi, Claudia Regina Gonçalves, Erica Valessa Ramos Gomes, Karoline Rodrigues Campos
EPI_ISL_942375, EPI_ISL_942897, EPI_ISL_942898, EPI_ISL_942930, EPI_ISL_942931	Central Laboratory of Public Health of Rio Grande do Sul (Lacen-RS)	State Center for Health Surveillance of the Health Department of the State of Rio Grande do Sul (CEVS/SES-RS)	Barcellos R, Campos A, Crescente L, Da Silva A, Dornelles C, Fonseca V, Garay L, Godinho F, Gonzalez A, Gregianini T, Molina C, Salvato R, Schaurich A
EPI_ISL_943574, EPI_ISL_943575, EPI_ISL_943576, EPI_ISL_943577, EPI_ISL_943580, EPI_ISL_943581, EPI_ISL_943582, EPI_ISL_943584, EPI_ISL_943586, EPI_ISL_943587, EPI_ISL_943588, EPI_ISL_943589, EPI_ISL_943590, EPI_ISL_943591, EPI_ISL_943593, EPI_ISL_943595, EPI_ISL_943596			
see above	Central Laboratory of Public Health of Rio Grande do Sul (Lacen-RS)	State Center for Health Surveillance of the Health Department of the State of Rio Grande do Sul (CEVS/SES-RS)	Aline Campos, Amanda da Silva, Anelise Schaurich, Claudia Dornelles, Cynthia Molina, Fernanda Godinho, Lara Crescente, Leticia Garay, Regina Barcellos, Richard Salvato, Tatiana Gregianini, Vagner Fonseca
EPI_ISL_943973, EPI_ISL_943974, EPI_ISL_943975, EPI_ISL_943976, EPI_ISL_943977	LACEN do Estado de Tocantins	Instituto Adolfo Lutz, Interdiciplinary Procedures Center, Strategic Laboratory	Claudio Tavares Sacchi, Claudia Regina Gonçalves, Erica Valessa Ramos Gomes, Karoline Rodrigues Campos
EPI_ISL_943988	LACEN do Estado de Goias	Instituto Adolfo Lutz, Interdiciplinary Procedures Center, Strategic Laboratory	Claudio Tavares Sacchi, Claudia Regina Gonçalves, Erica Valessa Ramos Gomes, Karoline Rodrigues Campos
EPI_ISL_977471	Instituto Adolfo Lutz - Regional de Presidente Prudente	Instituto Adolfo Lutz, Interdiciplinary Procedures Center, Strategic Laboratory	Claudio Tavares Sacchi, Claudia Regina Gonçalves, Erica Valessa Ramos Gomes, Karoline Rodrigues Campos
EPI_ISL_977472, EPI_ISL_977473, EPI_ISL_977474	Instituto Adolfo Lutz Central	Instituto Adolfo Lutz, Interdiciplinary Procedures Center, Strategic Laboratory	Claudio Tavares Sacchi, Claudia Regina Gonçalves, Erica Valessa Ramos Gomes, Karoline Rodrigues Campos
EPI_ISL_977475	Instituto Adolfo Lutz - Regional de Presidente Prudente	Instituto Adolfo Lutz, Interdiciplinary Procedures Center, Strategic Laboratory	Claudio Tavares Sacchi, Claudia Regina Gonçalves, Erica Valessa Ramos Gomes, Karoline Rodrigues Campos
EPI_ISL_977477	Instituto Adolfo Lutz Central	Instituto Adolfo Lutz, Interdiciplinary Procedures Center, Strategic Laboratory	Claudio Tavares Sacchi, Claudia Regina Gonçalves, Erica Valessa Ramos Gomes, Karoline Rodrigues Campos
EPI_ISL_977478	Instituto Adolfo Lutz - Regional de Presidente Prudente	Instituto Adolfo Lutz, Interdiciplinary Procedures Center, Strategic Laboratory	Claudio Tavares Sacchi, Claudia Regina Gonçalves, Erica Valessa Ramos Gomes, Karoline Rodrigues Campos
EPI_ISL_977479	Lab Loc - Itapecerica da Serra	Instituto Adolfo Lutz, Interdiciplinary Procedures Center, Strategic Laboratory	Claudio Tavares Sacchi, Claudia Regina Gonçalves, Erica Valessa Ramos Gomes, Karoline Rodrigues Campos
EPI_ISL_977480, EPI_ISL_977481	Instituto Adolfo Lutz - Regional de Presidente Prudente	Instituto Adolfo Lutz, Interdiciplinary Procedures Center, Strategic Laboratory	Claudio Tavares Sacchi, Claudia Regina Gonçalves, Erica Valessa Ramos Gomes, Karoline Rodrigues Campos
EPI_ISL_977483, EPI_ISL_977484	Instituto Adolfo Lutz Central	Instituto Adolfo Lutz, Interdiciplinary Procedures Center, Strategic Laboratory	Claudio Tavares Sacchi, Claudia Regina Gonçalves, Erica Valessa Ramos Gomes, Karoline Rodrigues Campos
EPI_ISL_977485	Instituto Adolfo Lutz - Regional de Presidente Prudente	Instituto Adolfo Lutz, Interdiciplinary Procedures Center, Strategic Laboratory	Claudio Tavares Sacchi, Claudia Regina Gonçalves, Erica Valessa Ramos Gomes, Karoline Rodrigues Campos
EPI_ISL_977486	Instituto Adolfo Lutz - Regional de Santo Andre	Instituto Adolfo Lutz, Interdiciplinary Procedures Center, Strategic Laboratory	Claudio Tavares Sacchi, Claudia Regina Gonçalves, Erica Valessa Ramos Gomes, Karoline Rodrigues Campos
EPI_ISL_977488	Instituto Adolfo Lutz - Regional de Presidente Prudente	Instituto Adolfo Lutz, Interdiciplinary Procedures Center, Strategic Laboratory	Claudio Tavares Sacchi, Claudia Regina Gonçalves, Erica Valessa Ramos Gomes, Karoline Rodrigues Campos
EPI_ISL_978488, EPI_ISL_978490, EPI_ISL_978493, EPI_ISL_978495, EPI_ISL_978496, EPI_ISL_978497, EPI_ISL_978498, EPI_ISL_978499, EPI_ISL_978500	Central Public Health Laboratory - LACEN -Bahia, Salvador, Brazil	Central Public Health Laboratory - LACEN -Bahia, Salvador, Brazil	Stephane Tosta, Luciana Oliveira, Vanessa Nardy,Patrícia Cajado,Marcela Gómez, Breno Dominguez, Jaqueline Gomes, Vagner Fonseca,Marta Giovanetti,Luiz Alcantara, Felicidade Pereira, Arabela Leal
EPI_ISL_984242	Instituto Adolfo Lutz Central	Instituto Adolfo Lutz, Interdiciplinary Procedures Center, Strategic Laboratory	Claudio Tavares Sacchi, Claudia Regina Gonçalves, Erica Valessa Ramos Gomes, Karoline Rodrigues Campos
EPI_ISL_984243, EPI_ISL_984244	Instituto Adolfo Lutz - Regional de Marilia	Instituto Adolfo Lutz, Interdiciplinary Procedures Center, Strategic Laboratory	Claudio Tavares Sacchi, Claudia Regina Gonçalves, Erica Valessa Ramos Gomes, Karoline Rodrigues Campos
EPI_ISL_984246	Instituto Adolfo Lutz Central	Instituto Adolfo Lutz, Interdiciplinary Procedures Center, Strategic Laboratory	Claudio Tavares Sacchi, Claudia Regina Gonçalves, Erica Valessa Ramos Gomes, Karoline Rodrigues Campos
EPI_ISL_984263	IAL Regional de Bauru	Instituto Adolfo Lutz, Interdiciplinary Procedures Center, Strategic Laboratory	Claudio Tavares Sacchi, Claudia Regina Gonçalves, Erica Valessa Ramos Gomes, Karoline Rodrigues Campos
EPI_ISL_985170	Instituto Adolfo Lutz - Regional de Presidente Prudente	Instituto Adolfo Lutz, Interdiciplinary Procedures Center, Strategic Laboratory	Claudio Tavares Sacchi, Claudia Regina Gonçalves, Erica Valessa Ramos Gomes, Karoline Rodrigues Campos
EPI_ISL_985171, EPI_ISL_985172, EPI_ISL_985173, EPI_ISL_985174	Instituto Adolfo Lutz - Regional de Taubate	Instituto Adolfo Lutz, Interdiciplinary Procedures Center, Strategic Laboratory	Claudio Tavares Sacchi, Claudia Regina Gonçalves, Erica Valessa Ramos Gomes, Karoline Rodrigues Campos
EPI_ISL_985175, EPI_ISL_985176, EPI_ISL_985177	Instituto Adolfo Lutz Central	Instituto Adolfo Lutz, Interdiciplinary Procedures Center, Strategic Laboratory	Claudio Tavares Sacchi, Claudia Regina Gonçalves, Erica Valessa Ramos Gomes, Karoline Rodrigues Campos
EPI_ISL_985178	Lab Loc - Itapecerica da Serra	Instituto Adolfo Lutz, Interdiciplinary Procedures Center, Strategic Laboratory	Claudio Tavares Sacchi, Claudia Regina Gonçalves, Erica Valessa Ramos Gomes, Karoline Rodrigues Campos
